# BET inhibition disrupts the FOXM1-MYC axis to induce BRCAness and enhance PARP inhibitor response

**DOI:** 10.1038/s41698-026-01360-x

**Published:** 2026-03-12

**Authors:** Pingping Fang, Anais Saunders, Kay Minn, Katherine Jane Chua, Rebecca A. Brooks, Danika Bakke, Gary S. Leiserowitz, Neil Johnson, Shaomeng Wang, Jeremy Chien

**Affiliations:** 1 Department of Pharmacology, Toxicology, and Therapeutics, University of Kansas Medical Center, Kansas City, KS USA; 2 Department of Biochemistry and Molecular Medicine, University of California Davis, Sacramento, CA USA; 3 Novogene, Sacramento, CA USA; 4 Department of Obstetrics and Gynecology, University of California Davis, Sacramento, CA USA; 5 Caris Life Sciences, Phoenix, AZ USA; 6 Nuclear Dynamics and Cancer Program, Fox Chase Cancer Center, Philadelphia, PA USA; 7 Department of Internal Medicine, Pharmacology and Medicinal Chemistry, University of Michigan, Ann Arbor, MI USA

**Keywords:** Cancer, Cell biology, Molecular biology, Oncology

## Abstract

Homologous recombination (HR) proficiency underlies intrinsic and acquired resistance to PARP inhibitors (PARPi). We identify a BRD4-dependent FOXM1-MYC transcriptional axis that sustains HR gene expression and limits PARPi response. ENCODE analyses revealed extensive co-occupancy of FOXM1 and MYC at regulatory regions of DNA repair genes, including BRCA1/2 and RAD51 paralogs, suggesting a shared HR program. Functionally, transient knockdown of FOXM1 or MYC reduced BRCA1/RAD51 transcripts, whereas sustained FOXM1 silencing triggered adaptive MYC upregulation that preserved HR output, indicating compensatory control. BET inhibition with (+)-JQ1 diminished FOXM1/MYC promoter occupancy at BRCA1 and RAD51, downregulated HR genes, and synergized with PARPi in viability and clonogenic assays. A BRD4 degrader (ZBC260) achieved potent BRD4 depletion at low nanomolar doses, suppressed FOXM1/MYC and HR gene expression, enhanced PARP1 trapping, and produced strong synergy with olaparib, including in patient-derived cancer cells. Clinically, BRD4 is highly expressed in ovarian cancer and independently predicts poor survival, outperforming FOXM1 and MYC. These data establish BRD4-directed disruption of the FOXM1–MYC axis as a strategy to induce “BRCAness” and broaden PARPi efficacy.

## Introduction

Cells with deleterious *BRCA1/2* mutations are hypersensitive to PARP inhibitor^1,2^. This observation was later extended to breast and ovarian cancer cells with a broader deficiency in HR pathway^3–5^. The expanded clinical activity of PARP inhibitors is due to “BRCAness” or HR-deficient (HRD) phenotypes resulting from defects in HR pathway that phenocopy *BRCA*-mutated tumors and hence sensitivity to PARP inhibitors. In high-grade serous ovarian cancer, approximately 50% of cases have HR defects^6^, which can result from either direct alterations of HR components, such as *BRCA1/2*^2,7^, or indirect alterations, such as *EMSY* amplification^8^. Ovarian cancer patients were among the first individuals to be evaluated for efficacy using PARP inhibitor^9–13^. The activity of PARP inhibitors was seen preferentially in ovarian cancer patients possessing a *BRCA* mutant tumor, leading to the approval of PARP inhibitors as upfront maintenance monotherapy therapy for ovarian cancer patients with mutated *BRCA1/2* (either germline or somatic) and those with a complete or partial response to platinum-based chemotherapy. Besides ovarian cancer, PARP inhibitors have shown activity in other cancer types, including breast cancer, prostate cancer, non-small cell lung cancer, and melanoma^14–18^. Based on successful data from the OlympiAD clinical trial (NCT02000622)^19^, olaparib gained FDA approval for germline BRCA-mutated metastatic breast cancer in early 2018.

In ovarian cancer, a clear survival advantage with PARP inhibitors was seen in patients with *BRCA* mutations and patients who are responsive to platinum-based chemotherapy. Unfortunately, the response rate in patients with no *BRCA* mutations and those with platinum-resistant disease is much less^9,10,20^. This selective activity largely limits the broader application of PARP inhibitors in cancer treatment. Meanwhile, like chemotherapy, resistance to PARP inhibitors eventually develops in patients^21^. One of the major causes of PARP inhibitor (PARPi) resistance in the clinic is the restoration of HR proficiency^22^. Drug combinations have been designed and evaluated in preclinical and early clinical trials to extend the therapeutic effect of PARP inhibitors and/or to overcome PARPi resistance^23^. These combinations were mostly designed with the goal of enhancing the cytotoxic effects, including PARPi in combination with ionizing radiation or chemotherapeutic agents^18,24,25^, such as topotecan, dacarbazine, and temozolomide^26–28^. Combination of PARPi with other agents, such as VEGF^29^, PI3K^30,31^, TGFβ^32^, and immune checkpoint inhibitors^33^ has also been explored. Even though preclinical studies have shown promising therapeutic effects with these combinations, results from clinical trials were disappointing. The dose-limiting severe hematological adverse effects were observed in the combination of olaparib with topoisomerase I inhibitor, Topotecan^34^, or with a potent VEGF inhibitor, Cediranib^29^. Therefore, there is an immediate need to explore new rational drug combinations that maximize clinical benefits and minimize adverse effects. These rational combinations must focus on compromising HR proficiency and restoring or enhancing PARP inhibitor sensitivity in cancer cells.

Previously, we showed that FOXM1 inhibition by thiostrepton disrupts the FOXM1 transcriptional network and enhances the sensitivity to PARP inhibitors (PARPi) in PARPi-resistant cancer cells^35^. Although the in vivo efficacy of thiostrepton has been shown by several groups^36–39^, further studies are needed to validate its clinical activity. Moreover, FOXM1 inhibition is only one of the documented activities of thiostrepton, and it was shown to function as a proteasome inhibitor as well^40,41^. In this regard, a more specific targeted drug may be desirable to combine with olaparib.

BET inhibitors are a class of drugs that were designed to reversibly bind the bromodomains of BET (bromodomain and extra-terminal motif) proteins (BRD2, BRD3, BRD4, and BRDT) to prevent their interactions with acetylated histones and transcription factors^42–44^ to decrease the transcription of target genes. Since BRD4 was shown to drive pathogenesis in various types of cancers^45^, BET inhibitors have been studied in cancers^46–50^. Importantly, BET inhibitors have been shown to block the expression of c-MYC (henceforth MYC)^51,52^, which was long considered undruggable oncogenic transcription factor^53^. In addition, in vitro and preclinical studies show that BET inhibitors are active in overcoming resistance to other targeted therapies when used in combination therapies^54,55^. Interestingly, Zhang et al. show that BET inhibitor downregulates FOXM1 pathway, but the molecular mechanisms associated with FOXM1 downregulation by BET inhibitors are not fully investigated^56^.

Here, we showed that FOXM1 inhibition leads to a compensatory increase in MYC expression that blunts the effect of FOXM1 inhibition, prompting us to address dual targeting of FOXM1 and MYC pathways with a BRD4 inhibitor or degrader. Our studies uncover the FOXM1-MYC nexus in regulating DNA repair genes and demonstrate that dual targeting of FOXM1 and MYC via BRD4 inhibition contributes to the drug synergies between PARP inhibitors and BET inhibitors. Our results show that dual targeting of FOXM1 and MYC with BET inhibitor (+)-JQ1 or BRD4 degrader ZBC260 effectively attenuates FOXM1 and MYC expression, induces “BRCAness,” and enhances sensitivity to PARP inhibitors.

## Results

### FOXM1 knockdown triggers compensatory MYC upregulation in ovarian cancer cells

**Fig. 1:**
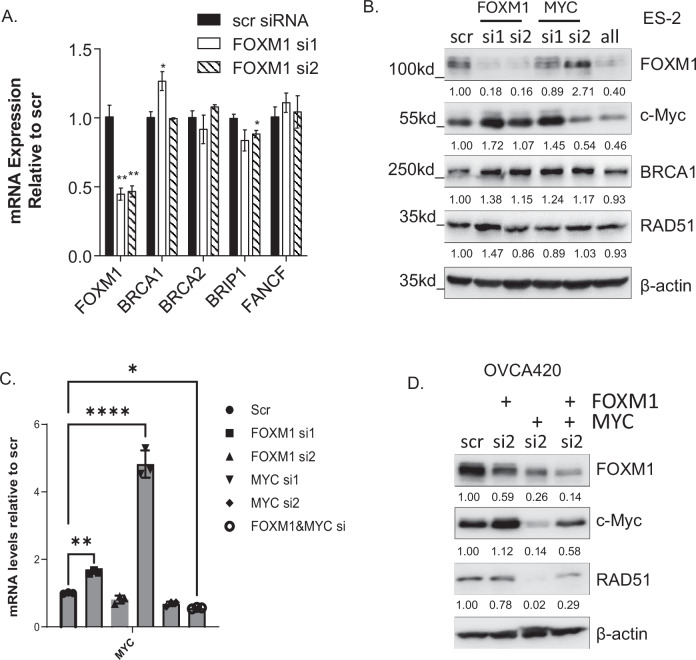
FOXM1 knockdown triggers compensatory MYC upregulation and jointly governs HR gene expression. **A** FOXM1 depletion, by siRNA transfection for 72 h, does not significantly reduce BRCA1/2 or FANCF mRNA but modestly decreases BRIP1, suggesting a limited effect on HR gene expression. **B, C** FOXM1 knockdown induces MYC, indicating an adaptive transcriptional response. Combined FOXM1 and MYC siRNAs attenuate both factors and reduce BRCA1 and RAD51 expression, revealing a compensatory FOXM1-MYC axis that affects HR gene expression. **D** OVCA420 cells exhibit similar MYC induction after FOXM1 knockdown and RAD51 suppression when both FOXM1 and MYC are targeted. Immunoblot signals were quantified by normalizing with β-actin and represented as fold change relative to scr siRNA. mRNA levels are normalized to scr control and expressed as mean ± S.E.M. in ES-2 cells transfected with siRNAs for 72 h. Unpaired t-tests between scr siRNA control and other groups were used to calculate *p*-values for (**A**). One-way ANOVA with Dunnett’s multiple comparisons test, with a single pooled variance, was used for panel C. Significant comparisons were indicated by asterisks: *, *p* < 0.05; **, *p* < 0.01; ****, *p* < 0.0001. all: all four siRNAs against FOXM1 and MYC.

FOXM1 has been shown to regulate DNA repair genes^57^. Therefore, we tested the effects of FOXM1 knockdown by siRNAs on the expression of known target genes of FOXM1 in the DNA repair pathway, including *BRCA1, BRCA2, BRIP1*, and *FANCF*. In previous studies, we have shown that ES-2 and OVCA420 are less sensitive to olaparib, but FOXM1 inhibitor thiostrepton enhances olaparib sensitivity in both cell lines^35^. Therefore, we selected these cell lines to further investigate the effect of FOXM1 knockdown. In ovarian cancer ES-2 cells at 72 h after siRNA transfection, two independent *FOXM1* siRNAs caused almost 50% reduction of *FOXM1* mRNA levels (Fig. 1A) and a more dramatic decrease in protein levels (Fig. 1B). However, we did not observe a significant decrease in mRNA levels of *BRCA1*, *BRCA2*, and *FANCF* after *FOXM1* knockdown in these cells. This finding suggests that FOXM1 is not the main transcription factor that regulates the expression of these genes, which is consistent with previous reports^58^. Meanwhile, we observed a small decrease in *BRIP1* mRNAs with *FOXM1* siRNAs, which indicates the direct regulation of *BRIP1* by FOXM1 and is consistent with a previous report^59^. The slight downregulation also suggests that FOXM1 is one of many transcription factors regulating the expression of *BRIP1*. Taken together, we conclude that FOXM1 downregulation is not sufficient to downregulate the expression of these target genes in the DNA repair pathway. Interestingly, we observed an increase in the levels of MYC protein after FOXM1 knockdown by siRNAs in ES-2 cells, particularly by si1 which led to 72% increase in MYC expression (Fig. 1B). Concordantly, we observed an increase in MYC mRNA by qRT-PCR following FOXM1 knockdown by si1 (Fig. 1C). When cells were transfected with all siRNAs simultaneously targeting both FOXM1 and MYC, we observed attenuation of both FOXM1 and MYC expression (Fig. 1B). Concordance with the reduction in FOXM1 and MYC expression, we observed a decrease in BRCA1 and RAD51 expression. In another cancer cell line OVCA420, we also observed the upregulation of MYC after FOXM1 knockdown (Fig. 1D) and a decrease in RAD51 expression following suppression of both FOXM1 and MYC expression by siRNAs. These data suggest that FOXM1 knockdown results in an increase in MYC expression and that both FOXM1 and MYC must be co-targeted to affect the expression of specific DNA repair genes.

### FOXM1 and MYC co-regulate HR genes and adaptive compensation

**Fig. 2:**
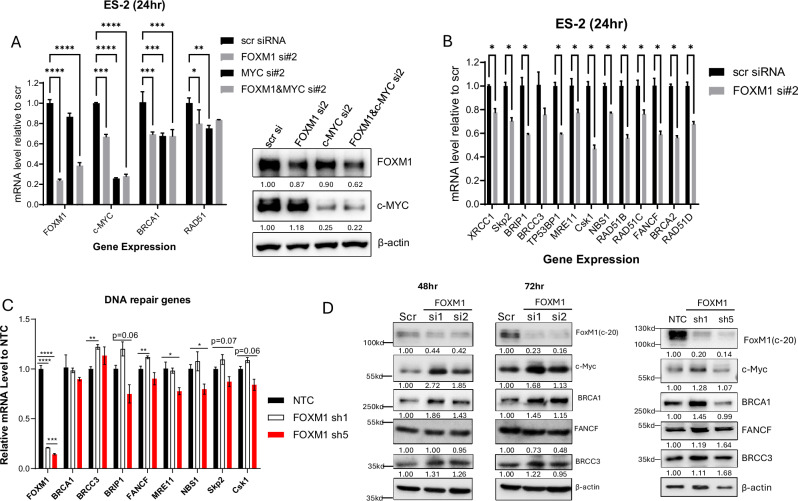
FOXM1 depletion reduces select DNA repair gene transcripts and reveals adaptive MYC compensation. **A** Transient knockdown of FOXM1 or MYC by siRNAs significantly decreases BRCA1 and RAD51 mRNA, indicating dual control of HR genes before adaptive rewiring occurs. Two-way ANOVA and Dunnett’s multiple comparisons test with simple effects within rows were used to calculate *p*-values. **B** Transient FOXM1 knockdown also lowers multiple DNA repair genes transcripts, reflecting an acute disruption of HR transcriptional programs. **C** In contrast, stable FOXM1 silencing (shRNA) shows selective reduction (e.g., MRE11, NSB1), consistent with compensatory rewiring that restores some HR gene expression over time. Student t-test. Significant comparisons were indicated by asterisks: *, *p* < 0.05; **, *p* < 0.01; ***, *p* < 0.001; ****, *p* < 0.0001. **D** MYC protein increases following FOXM1 knockdown, reinforcing a compensatory nexus that preserves HR gene expression unless both factors are suppressed. These findings highlight the temporal dynamics of FOXM1-MYC nexus and the need for dual targeting to overcome adaptive resistance.

ENCODE ChIP-seq analyses revealed extensive co-occupancy of FOXM1 and MYC across the genome, with 6453 regions showing ≥70% overlap; 62% of these overlapped sites localized to regulatory regions near genes (Supplementary Fig. S1), including multiple DNA repair genes (*ATM*, *ATR*, *BRIP1*, *RAD51B*, *RAD51D*, *RAD50*, *XPC*, and *XRCC3*) (Supplementary Table S1). Disease Ontology enrichment highlighted “hereditary breast ovarian cancer syndrome” (Supplementary Fig. S1). These data point to FOXM1-MYC transcriptional axis controlling DNA repair gene expression and provide a rationale for dual targeting to induce “BRCAness” and potentiate PARP inhibition. Functional perturbation corroborated this model. In ES-2 cells, transient knockdown of FOXM1 or MYC reduced BRCA1 and RAD51 transcripts (Fig. 2A), and FOXM1 depletion suppressed additional DNA repair genes (Fig. 2B). By contrast, stable FOXM1 silencing diminished MRE11 and NBS1 (Fig. 2C) only when FOXM1 silencing was not compensated by MYC upregulation (Fig. 2D), an adaptive response that can preserve HR proficiency. These findings indicate that effective disruption of HR transcriptional control requires concomitant suppression of FOXM1 and MYC.

### *FOXM1, MYC*, and *BRD4* transcripts are elevated in ovarian cancer and BRD4 expression stratifies patient survival

Bromodomain-containing protein 4 (BRD4) is a member of the BET (bromodomain and extra-terminal domain) protein family and has been implicated in promoting gene transcription through interaction with the transcription elongation factor P-TEFb and RNA polymerase II^60^. *MYC* expression is positively regulated by BRD4^51^. Studies have shown that BRD4 also regulates *FOXM1* expression^56^. We therefore analyzed the expression of *BRD4* along with *FOXM1* and *MYC* in TCGA transcriptomic data. Ovarian carcinomas exhibit among the highest expression levels of *FOXM1* and *MYC* across cancer types, and the highest levels of BRD4 (Supplementary Fig. S2), which is consistent with the observation from Zhang et al.^56^. *FOXM1* and *MYC* showed a modest negative correlation, consistent with our observation that *FOXM1* knockdown leads to upregulation of MYC (Figs. 1 and 2). *BRD4* expression is positively correlated with *FOXM1* and, to a lesser extent, with MYC, which is consistent with previous reports^51,56^. A positive association between BRD4 and FOXM1 expression is further reinforced by their significant co-occurrence in BRD4-high and FOXM1-high tumors (Supplementary Table S2). Taken together, these data suggest that BRD4 may act upstream to sustain the FOXM1-MYC transcriptional program and the associated DNA repair network.

**Fig. 3:**
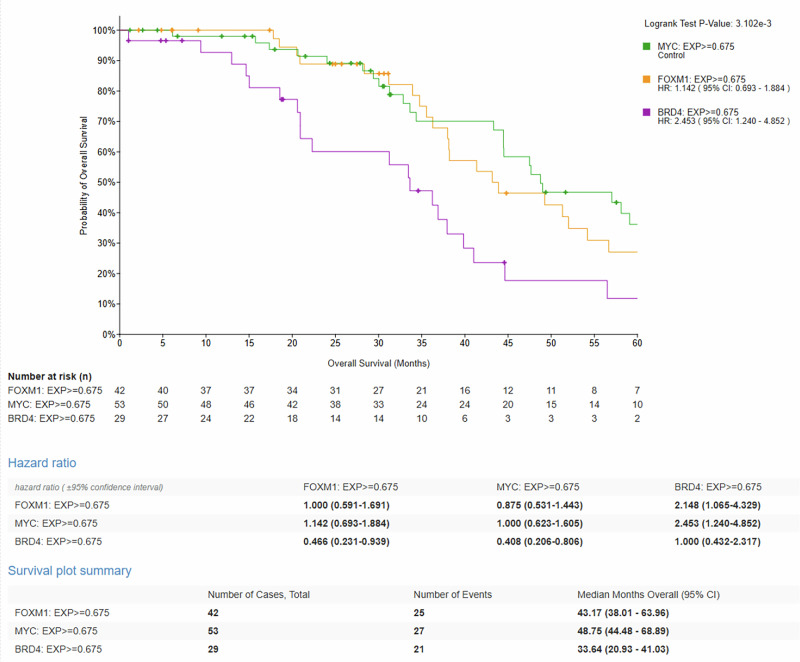
High BRD4 expression is associated with poor overall survival in HGSC and outperforms FOXM1/MYC as an independent prognostic factor. Among the top-quartile subsets, BRD4 high-expression confers worse outcomes than FOXM1 or MYC high-expression, underscoring BRD4 as a clinically actionable node and stronger prognostic indicator than either FOXM1 or MYC alone. Only those patients with BRD4, FOXM1, or MYC expression at the top quartiles are included. Overlapping samples (samples belonging to two or more groups) were excluded. The log-rank test was used to test the null hypothesis that there is no difference in survival between groups. *P*-value = 3.102e-3 (Log-rank test). Hazard ratio in the Kaplan–Meier plot was calculated relative to the MYC expression group. Hazard ratios relative to each group are also included.

To assess clinical relevance, we evaluated *BRD4*, *FOXM1*, and *MYC* expression in high-grade serous ovarian cancer. *BRD4* high-expression tumors (top quartile) were consistently associated with poor overall survival in both TCGA and Caris Life Sciences datasets, whereas FOXM1 and MYC expression alone were not prognostic (Supplementary Fig. S3). Multivariate analysis identified *BRD4* expression as an independent predictor of outcome (Supplementary Table S3), and BRD4-high tumors conferred worse survival than tumors with high *FOXM1* or *MYC* expression (Fig. 3).

### BRD4 expression in the 13p13.12 amplicon is a critical factor associated with poor prognosis

**Fig. 4:**
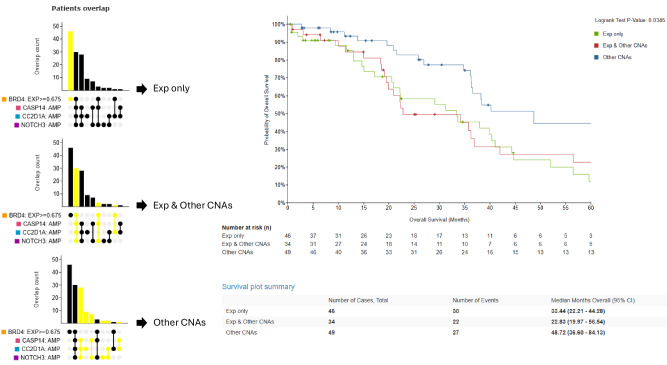
Focal 19p13.12 copy number alterations co-occur with high BRD4 expression, establishing BRD4 as a key amplicon driver linked to poor outcome. Patients harboring other CNAs without high BRD4 expression exhibit significantly better outcomes, underscoring BRD4’s dominant role in shaping clinical behavior and therapeutic response. Yellow bar(s) indicate membership in the group.

The HGSC dataset from TCGA indicates tumors with top quartile expression of *BRD4* have significantly lower mutation counts compared to those with bottom quartile expression (Supplementary Fig. S4A). However, no specific gene mutations are significantly enriched in either cohort (Supplementary Fig. S4B–D). Interestingly, specific copy number alterations (CNAs) are significantly enriched in the samples with high *BRD4* expression (Supplementary Fig. S4E–G). Many of these CNAs reside within the 19p13.12 locus, and there is a significant co-occurrence of these alterations together with high *BRD4* expression in the TCGA dataset (Supplementary Table S4), suggesting these associations are the result of focal amplification. Previous reports indicate that CCD21A and NOTCH3 expressions were associated with poor outcomes in ovarian cancer^61,62^. To test the extent to which the association between *BRD4* expression and poor outcome is the result of co-amplified genes, such as *CC2D1A* and *NOTCH3*, we identified samples with amplifications of these genes with or without *BRD4* high expression and performed Kaplan–Meier analysis. The results indicate that patients with other CNAs without high *BRD4* expression have significantly better outcomes than those with high *BRD4* expression (Fig. 4). There is no significant difference in patient outcomes between those with high *BRD4* expression without other CNAs and those with other CNAs (Fig. 4).

### BET inhibitor (+)-JQ1 induces “BRCAness” in ovarian cancer cells

BET inhibitors are small molecules that bind to the BET bromodomains and block the interaction of BET proteins (BRD2,3,4 and BRDT) with acetylated lysine residues on histone tails to regulate gene transcription^45,63^. They have been shown to block MYC expression in pre-clinical cancer models. BET inhibitors have also been reported to disrupt the FOXM1 pathway and show efficacy in ovarian carcinomas^56^. We propose to use a BET inhibitor to inhibit BRD4 and downregulate both MYC and FOXM1, thereby suppressing the expression of target genes regulated by either or both, especially the HR genes, and inducing “BRCAness” to sensitize cancer cells to a PARP inhibitor.

**Fig. 5:**
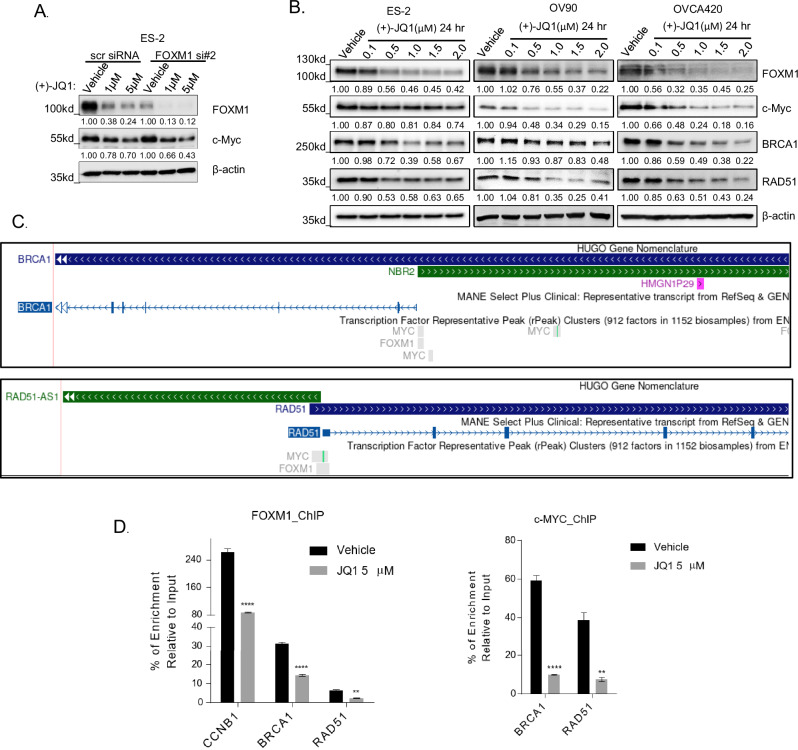
BET inhibition with (+)-JQ1 induces “BRCAness” by suppressing FOXM1/MYC and diminishing promoter occupancy at BRCA1/RAD51. **A** (+)-JQ1 effectively lowers FOXM1 expression while it was less efficient in lowering MYC expression. Forty-eight hours after siRNA transfection, vehicle, 1 μM or 5 μM of (+)-JQ1 were added to ES-2 cells and treated for another 24 h before the Western blot analysis. **B** (+)-JQ1 downregulates FOXM1, MYC and their targets, *BRCA1* and *RAD51*, in a dose-dependent manner across three different cell lines. **C** ENCODE-derived promoter maps confirm FOXM1 and MYC binding sites within BRCA1 and RAD51 regulatory regions. Cognate TF motif sites from previous studies^85^ are highlighted in green when they overlap with the ChIP signal. **D** ChIP-qPCR shows that (+)-JQ1 significantly reduces FOXM1 and MYC occupancy at BRCA1 and RAD51 promoters, directly linking FOXM1 and MYC to impaired HR gene transcription and induction of BRCAness by (+)-JQ1. These findings provide a rationale for combining BET inhibitors with PARP inhibitors to overcome HR-driven resistance. Cells were treated with vehicle or 5 μM (+)-JQ1 for 24 h before ChIP analysis. *CCNB1* is a positive control for FOXM1 binding. Data are shown as % of enrichment relative to input, with mean ± S.E.M. Statistics analysis was done using the student’s t-test. **p < 0.01, ****p < 0.0001. Results are representatives from three independent experiments.

We used one of the pre-clinical BET inhibitors, (+)-JQ1, which has been shown to work in nanomolar concentrations^51^. First, we tested whether (+)-JQ1 can downregulate MYC expression induced by *FOXM1* knockdown. In the ES-2 cell, we observed downregulation of both FOXM1 and MYC with (+)-JQ1 treatment in the control siRNA-transfected group, with FOXM1 being downregulated to a greater extent than MYC (Fig. 5A). (+)-JQ1 treatment also attenuated the MYC expression induced by the *FOXM1* knockdown, and (+)-JQ1 further decreases FOXM1 expression (Fig. 5A). The extent of MYC downregulation is not as robust as FOXM1, suggesting that MYC expression is not primarily dependent on BET proteins in these cells. Consistent with these results, there is a significant co-occurrence between high *BRD4* expression (top quartile) and high *FOXM1* expression, but not between *BRD4* and *MYC*, nor between *FOXM1* and *MYC* (Supplementary Table S4).

The analysis of TCGA dataset indicates that *BRCA1*, *BRCA2*, *RAD51*, and several HR gene transcripts are significantly higher in samples with high *BRD4* or *FOXM1* transcripts (Supplementary Fig. S5A, B), consistent with prior studies reporting a direct regulation of BRCA1 and RAD51 by BRD4 and FOXM1^64–66^ In contrast, expressions of these genes are not significantly higher in samples with high *MYC* expression (Supplementary Fig. S5C). Interestingly, expressions of these genes are significantly higher in tumors with high FOXM1 expression compared to those with high BRD4 or MYC expression (Supplementary Fig. S5D).

Next, we tested if (+)-JQ1 can downregulate HR genes in three cancer cell lines, OVCA420, OV90, and ES-2, that show low sensitivity to olaparib^35,67^. Twenty-four hours after (+)-JQ1 treatment, we saw a consistent dose-dependent downregulation of FOXM1 in all three cell lines, and a consistent efficient downregulation of MYC with higher concentrations of (+)-JQ1 in OVCA420 and OV90 cells (Fig. 5B). At the same time, we examined the expression of two HR genes, *BRCA1* and *RAD51*, whose expression is regulated by both FOXM1 and MYC according to our analysis of ENCODE data (Fig. 5C). We saw a consistent downregulation of both BRCA1 and RAD51 by (+)-JQ1 in all three cell lines (Fig. 5B). We also performed ChIP (chromatin immunoprecipitation) analysis with FOXM1 or MYC pull down after (+)-JQ1 treatment in OVCA420 cells and observed that the binding of both FOXM1 and MYC to the promoters of both *BRCA1* and *RAD51* decreased significantly in cells treated with 5 μM (+)-JQ1 for 24 h compared to vehicle-treated groups (Fig. 5D). Collectively, these data suggest that the downregulation of *BRCA1* and *RAD51* by (+)-JQ1 is in part due to a decrease in transactivation by both FOXM1 and MYC through decreased binding to their promoter regions.

### (+)-JQ1 sensitizes cancer cells to the PARP inhibitor

**Fig. 6:**
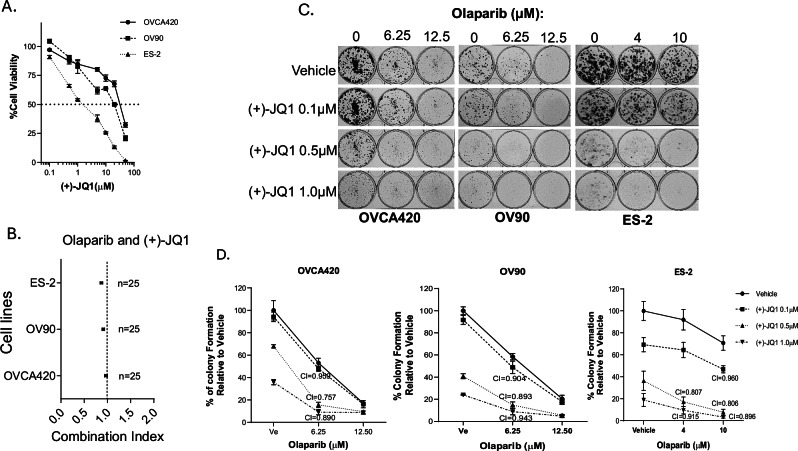
BET inhibition with (+)-JQ1 synergizes with olaparib in PARPi-insensitive ovarian cancer cells. **A** Short-term viability assay shows dose-dependent cytotoxicity of (+)-JQ1 in ES-2, OV90, and OVCA420 cells, indicating intrinsic sensitivity to BET inhibition. Data are shown as % of cell viability relative to the vehicle with mean ± S.E.M. **B** Combination of (+)-JQ1 and olaparib produces additive to weak synergistic effects in SRB assays, reflecting early cytotoxic interactions. **C, D** Long-term colony formation assays reveal stronger synergy: sub-IC50 doses of (+)-JQ1 combined with olaparib substantially decrease colony formation, demonstrating durable suppression of proliferative capacity. Combination index (CI) analysis confirms moderate synergy, supporting BET inhibition as a strategy to overcome HR-driven resistance and enhance PARPi efficacy. Drugs were washed away 3 days later, and cells were fed with fresh media every 3–4 days for two weeks. Colonies were stained with SRB, imaged, and counted. CI was calculated and indicated in the line graph for each combination. Line graph data are shown as % of colony formation relative to the vehicle with mean ± S.E.M.

Next, we tested whether (+)-JQ1 enhances PARP inhibitor sensitivity in cancer cells that are initially less sensitive to PARP inhibitors. First, we performed a short-term cell viability assay to test the effects of (+)-JQ1 in three different cancer cells, and we observed a dose-dependent decrease in cell viability with (+)-JQ1 treatment (Fig. 6A). We also observed that the IC_50_ for (+)-JQ1 in these cells are in micromolar range, and ES-2 cell is more sensitive to (+)-JQ1 compared to the other two cells. Next, we used the SRB assay to test the effects of (+)-JQ1 in combination with the FDA-approved PARP inhibitor olaparib. We observed a weak synergy between (+)-JQ1 and olaparib in ES-2 and OV90 cells, and an additive effect in OVCA420 cells (Fig. 6B). We also performed a long-term colony formation assay (Fig. 6C–E). (+)-JQ1 treatment by itself leads to a reduction of colony formation in all three cells and results in a complete inhibition of colony formation when combined with sub-lethal doses of olaparib. We calculated the combination indexes and observed a moderate synergistic effect between (+)-JQ1 and olaparib. These data suggest that (+)-JQ1 enhanced the cytotoxic effect of olaparib in these cancer cells with primary resistance to the PARP inhibitor.

To test whether the BET inhibitor can resensitize cancer cells with acquired resistance to the PARP inhibitor, we used rucaparib-resistant breast cancer cell lines that were derived from MDA-MB-436 after a continuous exposure to the PARP inhibitor rucaparib^68^, named as RR-1 and RR-2. These cells are about 500-fold less responsive to rucaparib than parental MDA-MB-436 cells^68^ based on colony formation assay. Using Western blotting, we observed that (+)-JQ1 treatment consistently led to decreased FOXM1 expression in two different resistant clones; however, we only saw MYC being downregulated moderately with a high concentration of (+)-JQ1 in RR-1 and no decrease in RR-2 (Supplementary Fig. S6A). RAD51 protein was consistently downregulated by (+)-JQ1 treatment. The resistance to rucaparib was mediated by the stabilization of the mutant BRCA1 (mtBRCA1) protein in these cells, and the resistance can be overcome by HSP90 inhibitor, which leads to the downregulation of stabilized mtBRCA1^68^. Interestingly, we also saw a dramatic decrease in mtBRCA1 protein level with (+)-JQ1 treatment. Next, we performed a colony formation assay to assess the effect of (+)-JQ1 and rucaparib. Similar to PARPi-insensitive cancer cells, we observed a dose-dependent decrease in colony formation in these resistant cells with (+)-JQ1 treatment, with RR-1 being more responsive than RR-2. This is consistent with the Western blot results that demonstrated MYC is not downregulated by (+)-JQ1 treatment in RR-2 but is moderately downregulated in RR-1. Consistent with the downregulation of both FOXM1 and MYC by (+)-JQ1 treatment, RR-1 showed a greater extent of the decrease in colony formation (Supplementary Fig. S6B, C). Strikingly, in combinations of 1 μM rucaparib with 500 nM (+)-JQ1, or 2.5 μM rucaparib with 300 nM (+)-JQ1, the colony formation was completely inhibited in RR-1. In RR-2, combinations of 1 μM (+)-JQ1 and sub-lethal doses of rucaparib also completely inhibited colony formation (Supplementary Fig. S6D, E). Combination indexes indicate an overall moderate synergistic effect between (+)-JQ1 and rucaparib (Supplementary Fig. S6C and Fig. S6E). Taken together, these data suggest that (+)-JQ1 treatment can help restore sensitivity to rucaparib in cancer cells with acquired resistance to rucaparib in part by downregulating FOXM1 and MYC and their downstream target genes *RAD51* and *mtBRCA1*. These results indicate the potential of using BET inhibitors to overcome PARP inhibitor resistance in cancer cells.

### PROTAC-based BRD4 degrader downregulates *BRCA1* and *RAD51 and enhances PARP1 trapping*

**Fig. 7:**
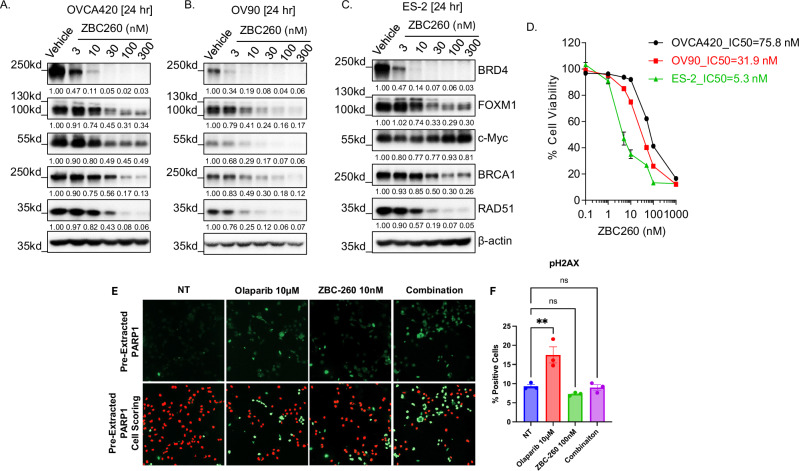
ZBC260 potently depletes BRD4, downregulates FOXM1/MYC and HR genes, and enhances PARP1 trapping. **A**–**C** Western blot analysis shows ZBC260 achieves near-complete BRD4 depletion starting at 10 nM, with subsequent reductions in FOXM1 across all cell lines (OVCA420, OV90, ES-2). MYC downregulation is cell-type dependent, occurring in OVCA420 and OV90 but not ES-2. HR genes BRCA1 and RAD51 are consistently suppressed, indicating disruption of homologous recombination signaling. **D** SRB viability assays reveal a dose-dependent cytotoxic effect of ZBC260, with IC50 values in the low nanomolar range, demonstrating superior potency compared to (+)-JQ1. These findings establish BET degradation as a highly effective strategy to induce BRCAness and sensitize HR-proficient cancer cells to PARP inhibitors. Data are shown as percent (%) of cell viability relative to the vehicle, with mean ± S.E.M. IC_50_s estimated from dose-response curves are shown. **E** Representative immunofluorescence images of pre-extracted PARP1 in OVCA420 cells treated with olaparib, ZBC260, or their combination. Combination treatment markedly increases PARP1 retention on chromatin compared to single agents, indicating enhanced PARP1 trapping—a key mechanism of PARP inhibitor cytotoxicity. **F** High-content image analysis quantifies PARP1-positive cells (>1000 cells/replicate), confirming significant enrichment in the combination group. These findings demonstrate that BET degradation potentiates PARP inhibitor activity by amplifying PARP1 trapping, a critical determinant of therapeutic efficacy. Cell scoring for pre-extracted PARP1 positive (green) and negative (red) cells as determined with the CellReporterXpress from Molecular Devices (v2.9.4.19394). Summary data of pre-extracted PARP1 staining includes analysis of >1000 cells/replicate. All data shown are the mean (SEM). Statistical analysis was performed using an ordinary one-way ANOVA with multiple comparisons to the NT (non-treated) group. ns non-significant, * *p* < 0.05, **p < 0.005, ***p < 0.0005.

BET inhibitor (+)-JQ1 has not been evaluated in the clinic because of its short half-life observed in a preclinical in vivo study^42^. Considering the potential clinical use of the BET inhibitor in combination with a PARP inhibitor, we tested a recently developed PROTAC (proteolysis targeting chimera)-based BRD4 degrader (ZBC260), which has been shown to degrade BRD proteins with a greater potency^69^. We tested the effects of ZBC260 in three different cell lines and observed that ZBC260 efficiently decreases BRD4 levels starting from as low as 3 nM, and BRD4 was completely depleted with concentrations >10 nM (Fig. 7A–C). These initial results suggest that ZBC260 is very efficient in suppressing and inhibiting BRD4 in our cells. In all three cell lines, FOXM1 was consistently downregulated by ZBC260 following a decrease of BRD4 (Fig. 7A–C). However, only in OVCA420 and OV90, we saw downregulation of MYC (Fig. 7A, B). Tested target genes of FOXM1 and MYC, such as *BRCA1* and *RAD51*, were also consistently downregulated following ZBC260 exposure to a greater extent at drug concentrations that are lower than (+)-JQ1 (see Fig. 7B–D). We also tested the drug with short-term SRB assay and observed a dose-dependent reduction of cell viability with much lower concentrations of ZBC260 compared to (+)-JQ1 (see Fig. 6A). Similar to (+)-JQ1, three cells responded to ZBC260 differently, with ES-2 cell being more sensitive than the other two cell lines (Fig. 7D). These data suggest that BET protein degrader is effective in downregulating FOXM1, MYC, and DNA repair genes, such as *BRCA1* and *RAD51* and is more potent than (+)-JQ1.

To test the effect of ZBC260 on PARP1 trapping, we treated OVCA420 cells with either olaparib or ZBC260 alone or together for 24 h, and high-content image analysis was performed on immunofluorescence micrographs stained for trapped PARP1. Results indicate a significant increase in trapped PARP1 in cells treated with both olaparib and ZBC260 (Fig. 7E, F). No increase in PARP1 staining was observed in cells treated with the combination if pre-extraction step is not performed as previously reported^70^ (Supplementary Fig. S7).

### Olaparib increases pH2AX staining in S and G2/M phases of cancer cells

**Fig. 8:**
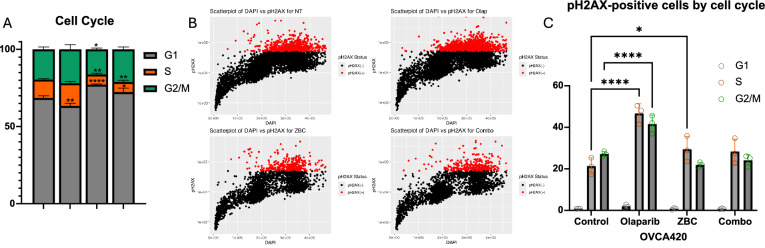
Olaparib induces DNA damage signaling predominantly in S and G2/M phases. **A** Cell-cycle distribution analysis using DAPI intensity shows a decrease in G1 cells and a trend toward increased S-phase following olaparib treatment, consistent with replication-associated stress. **B**–**C** High-content imaging reveals a significant increase in pH2AX-positive cells in S and G2/M phases after olaparib exposure, confirming DNA damage accumulation during replication and mitosis. The combination with ZBC260 does not further elevate pH2AX but markedly reduces total cell counts, indicating strong cytotoxicity driven by BRD4 degradation. Statistical analysis was performed using an ordinary two-way ANOVA (**A**) or one-way ANOVA (**C**) with Dunnett’s multiple comparisons test to the Control group. * *p* < 0.05, **p < 0.005, ***p < 0.0005, and ****p < 0.0001.

High-content image analysis can be used to quantify nuclear DAPI fluorescence intensity, which can be used to identify cell cycle-related mechanisms^70^ (Supplementary Fig. S8). Results of the high-content image analysis indicate a significant decrease in G1 cells and a noticeable but non-significant increase in S phase cells following olaparib treatment (Fig. 8A). Consistent with prior studies, a significant increase in pH2AX-positive cells was observed in cells in S and G2/M phases (Fig. 8B, C). Interestingly, the combination does not increase pH2AX (Fig. 8C and Supplementary Fig. S9). It is important to note that a significant decrease in cell counts was observed in cells treated with either ZBC260 or the combination (Supplementary Fig. S10), indicating the cytotoxic effects of these treatments.

### ZBC260 is synergistic with PARP inhibitors in ovarian cancer cell lines

**Fig. 9:**
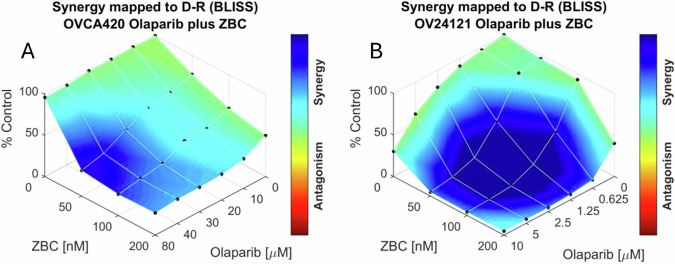
ZBC260 and olaparib exhibit strong synergy in ovarian cancer models, including patient-derived primary cells. **A** Combenefit synergy analysis (BLISS model) for OVCA420 cells demonstrates robust synergistic interactions across multiple dose combinations of ZBC260 and olaparib, indicating enhanced cytotoxicity beyond additive effects. **B** Similar synergy patterns are observed in primary malignant ascites culture (OV24121) from a patient with advanced high-grade serous carcinoma, reinforcing translational relevance. These findings highlight BRD4 degradation as a clinically actionable strategy to potentiate PARP inhibitor efficacy in HR-proficient ovarian cancer.

Given the cytotoxic effect of ZBC260 and its ability to downregulate BRD4, MYC, FOXM1, and specific HR repair genes, we assess its potential to enhance sensitivity to olaparib. We treated OVCA420 and the primary culture of malignant ascites (OV24121) from a patient with advanced high-grade serous carcinomas with varying doses of olaparib and ZBC260. The analysis of potential synergies between two drugs using the Combenefit software indicates strong synergies between these agents in these cells, all three classical Synergy models (Fig. 9 and Supplementary Fig. S11).

## Discussion

Our data establish BET inhibition as a strategy to overcome both intrinsic and acquired resistance to PARP inhibitors by disrupting the FOXM1–MYC transcriptional axis, downregulating key homologous recombination (HR) genes, and inducing a “BRCAness” phenotype. Across ovarian (ES‑2, OV90, OVCA420) and breast (MDA‑MB‑436 derivatives) models, BET blockade ((+)‑JQ1) or BRD4 degradation (ZBC260) suppressed FOXM1-MYC axis, reduced BRCA1/RAD51 expression, enhanced PARP1 trapping, and produced durable combination effects with olaparib or rucaparib in clonogenic assays. These findings provide a mechanistic rationale for combining BET‑targeting agents with PARP inhibitors to extend efficacy in HR‑proficient disease.

Mechanistically, transient FOXM1 knockdown failed to durably suppress several HR genes (e.g., BRCA1/2, FANCF), coincidence with MYC upregulation, indicating an adaptive transcriptional rewiring that preserves HR proficiency unless both factors are inhibited. ENCODE analyses revealed extensive co‑occupancy of FOXM1 and MYC at regulatory regions of DNA repair genes, positioning this pair as co-regulators of the HR program. Consistent with this model, (+)‑JQ1 reduced FOXM1 and MYC promoter occupancy at BRCA1 and RAD51, directly linking BET inhibition to impaired HR gene transcription and induced BRCAness. Moreover, gene expression profiling showed that transient FOXM1 knockdown acutely lowers multiple DNA repair transcripts, whereas stable silencing is followed by selective reductions (e.g., MRE11, NBS1) and MYC induction, reflecting time‑dependent compensation.

This study expands on earlier observations that FOXM1 contributes to PARP inhibitor resistance and that thiostrepton can restore sensitivity by targeting FOXM1^35^. We uncovered a time-dependent compensatory response following FOXM1 inhibition. Within 24 h of FOXM1 knockdown, several HR genes were transcriptionally downregulated. However, within 48–72 h of FOXM1 knockdown, MYC was upregulated, and expression of several HR genes was restored. It is important to note that previous reports also failed to see the downregulation of FOXM1 target genes, such as *BRCA2* and *XRCC3*, following the FOXM1 knockdown^58^, suggesting that adaptive rewiring of transcriptional programs may be restoring the expression of these target genes. Our results put a cautionary note on strategies to target the FOXM1 network alone to achieve BRCAness.

By uncovering the FOXM1–MYC compensatory circuit and demonstrating dual suppression via BRD4 inhibition or depletion, we provide a viable strategy to target the HR pathway to induce BRCAness. Our findings complement prior reports that BET inhibition synergizes with PARP blockade through G2/M checkpoint perturbation and CtIP/RAD51 axis disruption, while adding the FOXM1/MYC layer that coordinately regulates BRCA1/2 and RAD51 expression. Prior studies by Karakashev et al. have shown that JQ1 synergizes with olaparib by downregulating Wee1 and TOPBP1 and by impairing the G2-M checkpoint, leading to increased apoptosis^71^. Our results provide an upstream mechanism by which BRD4 inhibition contributes to olaparib sensitivity by downregulating FOXM1 and MYC pathways and by preventing adaptive transcriptional responses, leading to downregulation of HR genes, such as BRCA1, BRCA2, and RAD51. Sun et al. have shown that BET inhibitors and degraders reduce CtIP expression and disrupt recruitment of RAD51 to DNA damage sites and impair HR activities, leading to enhanced PARPi sensitivity^72^. Our results complement these findings by demonstrating that BRD4 depletion blunts adaptive transcriptional responses that are mediated by the FOXM1-MYC axis and attenuates RAD51, BRCA1, and BRCA2 expressions. While Yang et al.^73^ focused on MYC and its target HR genes, we provided additional upstream components involving FOXM1 and MYC as compensatory regulators of HR gene expression.

Clinically, BRD4 high expression associates with poorer overall survival in high‑grade serous ovarian cancer (HGSC), exceeding the prognostic value of FOXM1 or MYC alone and supporting BRD4 as a clinically actionable node. Amplicon analyses further implicate 19p13.12 focal copy‑number alterations that co‑occur with elevated BRD4 expression; patients with other CNAs without high BRD4 expression fare better, underscoring BRD4’s dominant role in shaping clinical behavior and treatment responses. We also showed that there is a significant co-occurrence of high BRD4 expression and high FOXM1 expression, suggesting BRD4 as an important regulator of FOXM1 expression. Finally, our analysis indicates that HR repair gene expression is higher in tumor samples with either high BRD4 or FOXM1 expression, thereby providing a rationale for co-targeting BRD4 and FOXM1 in ovarian cancer. Our findings show that (+)-JQ1 and ZBC260 consistently suppress FOXM1, indicating that BET inhibition can co-target BRD4 and FOXM1. Given its effect on FOXM1 and HR gene expression, BRD4 emerges as a key therapeutic target for HR‑proficient, BRD4‑driven tumors.

(+)-JQ1 has been shown to successfully inhibit MYC expression and the MYC pathway in preclinical studies^51^. Recent studies also indicate that the (+)-JQ1 treatment results in the downregulation of FOXM1, although the molecular mechanism is not well-characterized^56^. Our results indicate that (+)-JQ1 can be used to attenuate compensatory MYC expression induced by the FOXM1 knockdown. We also observed a consistent downregulation of both FOXM1 and MYC, as well as the downregulation of *BRCA1* and *RAD51* in at least two different cancer cells. Interestingly, MYC expression decreases in OVCA420 and OV90, but not in ES-2, suggesting MYC expression in ES-2 is not BRD4-dependent, likely mediated by NFATC1^74^ via a BRAF mutation^75^. In contrast, FOXM1 expression was consistently reduced following (+)-JQ1 treatment, indicating that FOXM1 is at least partially dependent on BRD4 activity in these cells.

We also tested the cytotoxic effect of (+)-JQ1 in breast cancer cells with acquired resistance to the PARP inhibitor rucaparib. The two resistant clonal cells were derived from the originally sensitive MDA-MB-436 breast cancer cells with a chronic exposure to rucaparib^68^. The main mechanism of resistance in these cells is the restoration of HR function by the stabilization of mutant BRCA1 (mtBRCA1) protein, which otherwise was quickly degraded in MDA-MB-436 cells. From the original study^68^, the resistance in these cells can be overcome by HSP90 inhibitor, which leads to HSP90 inhibition and destabilization of mtBRCA1, resulting in HR deficiency and restoration of PARP inhibitor sensitivity. Interestingly, we found that (+)-JQ1 treatment resulted in downregulation of the originally stabilized mtBRCA1 protein in these cells, and this effect might be a result of decreased transcription caused by inhibition of FOXM1 and MYC. However, further studies are needed to uncover how (+)-JQ1 treatment led to decreased mtBRCA1 in these resistant cells.

Even though (+)-JQ1 had shown effectiveness in both in vitro and in vivo studies, its potential use for clinical evaluation was reconsidered because of its short half-life in vivo^42^. Based on our observations that the combination of (+)-JQ1 and PARP inhibitors gives better therapeutic effects, it is important to test the efficacy of this combination in clinical trials, and clinical trials rely on the availability of an effective and safe BET inhibitor candidate. As a result, we tested the effects of a recently developed PROTAC BET protein degrader, ZBC260, which has been tested as a single agent for in vivo studies^69^, but not in combination with a PARP inhibitor. Our data showed that it is effective in lowering FOXM1, MYC, BRCA1, and RAD51 expressions. It also inhibited cell growth in a dose-dependent manner with IC_50_s in the nanomolar range, suggesting that it is more potent than (+)-JQ1. In the future, the combined effects of ZBC260 and PARP inhibitors should be tested in cells with primary or acquired resistance to PARP inhibitors, followed by the determination of the combined effects in patient-derived xenograft models. PARP inhibitors in combination with (+)-JQ1 have already been tested in several cancer cell line xenograft^71,73^ and patient-derived xenograft mouse models^76^. Additional studies should focus on determining the combined effect in patient-derived xenograft models derived from HR-proficient cancer to demonstrate the extent to which BET degraders can extend the clinical utility of PARP inhibitors in treating patients with HR-proficient cancers.

Collectively, we showed that BET inhibition or BRD4 depletion inhibits the FOXM1-MYC nexus in cancer cells, downregulates the overlapping downstream target HR genes to induce “BRCAness,” and sensitizes resistant cells to PARP inhibitors, suggesting that the combination of BET inhibitor/degrader and PARP inhibitor might be a new strategy to enhance or expand the therapeutic effects of PARP inhibitors.

## Methods

### Cell lines and cell culture

ES-2 cell was maintained in MCDB105 and M199 (1:1) (Sigma, USA) containing 5% FBS (Sigma). OV90 cells were maintained in MCDB105 and M199 (1:1) with 15% FBS. OVCA420 cells were cultured in DMEM (Sigma and Caisson Labs, USA) supplemented with 10% FBS. MDA-MB-436-derived rucaparib-resistant cells RR-1, RR-2 were generated by Dr. Neil Johnson laboratory at Fox Chase Cancer Center^68^ and were maintained in RPMI1640 (Sigma and Caisson Labs) supplemented with 10% FBS. All the media were supplemented with 100 units/mL penicillin and 100 µg/mL streptomycin.

Ovarian cancer cell lines ES-2, OV90, and OVCA420 harbor *TP53* mutations and are considered serous adenocarcinomas^75,77^. No *BRCA1* or *BRCA2* mutations are reported in these cell lines^75,77^. ES-2 was established from the patient who was reported to be refractory to chemotherapy^77^. OVCA420 cells have the highest IC50 to veliparib among 20 ovarian cancer cell lines tested^77^. In another study consisting of 39 ovarian cancer cell lines, OV90 and OVCA420 are considered resistant to rucaparib, while ES-2 is considered moderately resistant to rucaparib^67^. Therefore, they can be classified as having intrinsic resistance to PARP inhibitors. In contrast, MDA-MB-436 has a deleterious mutation in BRCA1^78^, and they are initially sensitive to the PARP inhibitor rucaparib^68^. However, continuous exposure to rucaparib produced clones with acquired resistance with over a hundred-fold resistance to rucaparib in colony formation assays^68^.

### Antibodies and compounds

Rabbit polyclonal anti-FOXM1 antibody (C-20, sc-502) and rabbit polyclonal anti-BRCA1 antibody (C-20, sc-642) were purchased from Santa Cruz Biotechnology (Santa Cruz, CA, USA). Mouse monoclonal anti-RAD51 antibody (5B3/6, GTX23638) was from GeneTex (Irvine, CA, USA). Rabbit monoclonal anti-MYC antibody (Y69) was purchased from Abcam (Cambridge, MA, USA). Mouse monoclonal anti-beta actin antibody (A1978) was from Sigma-Aldrich (St Louis, MO, USA). For secondary antibodies, horse anti-mouse IgG-HRP antibody (7076S) was purchased from Cell Signaling Technologies, and goat anti-rabbit IgG-HRP antibody (sc-2030) was obtained from Santa Cruz Biotechnology. olaparib (AZD2281, Ku-0059436) and rucaparib were purchased from Selleckchem (TX, USA). Stock solutions were made with DMSO at 50 mM and stored at −80 °C. ZBC260 was provided by Dr. Shaomeng Wang from the University of Michigan. The stock solution was made with DMSO in 10 mM and aliquoted to store at −80 °C. (+)-JQ1 was kindly provided by the Bradner lab at Dana-Farber Cancer Institute. The stock solution was made in DMSO at 50 mM aliquots and stored at −80 °C.

### Immunoblotting

Cells were washed at least twice with PBS at the end of treatments, if applicable, and then lysed with an appropriate volume of 1X electrophoresis sample buffer (Bio-Rad Laboratories, CA, USA) with 5% β-mercaptoethanol (Sigma-Aldrich). The cell lysates were heated to 95 °C for 5 min before using. Immunoblotting procedures were performed as previously described^39^. For apoptosis markers, cells were collected at the end of treatments, and total proteins were extracted using RIPA buffer (1% NP-40, 0.5% sodium deoxycholate, and 0.1% SDS in 1X PBS) containing protease/phosphatase inhibitor cocktail (Roche). BCA protein assay reagent kit (Pierce) was used to determine protein concentrations. Equal amounts of total proteins were loaded for SDS-PAGE and transferred to PVDF membranes (GE Healthcare).

### Chromatin immunoprecipitation (ChIP)-qPCR

Chromatin Immunoprecipitation was carried out as described before^79^. Briefly, after 20 μM olaparib treatment for 12 h or 24 h, cells were cross-linked with 1% formaldehyde (Electron Microscopy Sciences, USA) for 10 min and quenched by cross-linking with excess glycine. The chromatin was sonicated with a Bioruptor Twin (Diagenode) at maximum setting for 12 min (30 s on, 30 s off). The sonicated chromatin was incubated with 1.0 μg FOXM1 antibody (C-20, sc-502, Santa Cruz Technology, USA) at 4 °C for 2–4 h before purification with 100 μL Protein A/G magnetic beads (88803, Pierce). The beads were washed five times with LiCl wash buffer (100 mM Tris pH7.5, 500 mM LiCl, 1% NP-40, 1% sodium deoxycholate), one time with TE buffer (10 mM Tris-HCl pH7.5 and 0.1 mM Na2EDTA), and were then eluted with Elution Buffer (1% SDS and 0.1 M NaHCO3). After reverse-crosslinking, the DNA was purified with the QIAQuick PCR cleanup kit (QIAGEN) and used for qPCR, which was performed in a CFX384 Touch^TM^ Real-Time PCR Detection System (Bio-Rad) using RT^2^ SYBR Green qPCR Master Mix (QIAGEN). Primer sequences are listed in Table 1.

**Table 1:** Primer sequences

Amplified target	Forward	Reverse
*FOXM1*(total)	5ʹAGAATTGTCACCTGGAGCAG	5ʹTTCCTCTCAGTGCTGTTGATG
*FANCF*	5’GCATTTGGGTTGGAACTGAG	5’CTTCAAAATCTCCATCCTGCG
*BRCA1*	5’TAATGCTATGCAGAAAATCTTAGAG	5’TACTTTCTTGTAGGCTCCTTTTGG
*BRCA2*	5’TTCATGGAGCAGAACTGGTG	5’AGGAAAAGGTCTAGGGTCAGG
*BRIP1*	5’GCTTAGCCTTACTTTGTTCTGC	5’TTTCACTTACGCCCTCATCTG
*BRCC3*	5’CCTCATGTCACTATCGGGAAAG	5’GGATCTTGGTTACTGAGTCCAG
*NBS1*	5’AGACCAACTCCATCAGAAACTAC	5’AATGAGGGTGTAGCAGGTTG
*CsK1*	5GAATGGAGGAATCTTGGCGTT	5’TCTTTGGTTTCTTGGGTAGTGGG
*Skp2*	5’CTGGGTGTTCTGGATTCTCTG	5’GCTGGGTGATGGTCTCTG
*XRCC1*	5’CCCCTGAAGAGACCAAAGC	5’GTCTGTGTTCCTTCTGCTCTG
*RAD51D*	5’TGGCTCAGTTCTCGGCTTTC	5’AGACATACCTGAGTTTTGCCG
*RAD51C*	5’AATGGCCTAGCCCAGCAAAT	5’TTGCCAACCTTTGCTTTCGG
*RAD51B*	5’AAGAGCTGTGTGACCGTCTG	5’TCCCATAAGCCGTTTGCATCT
*MRE11*	5’TGGGTGAACTATCAAGATGGC	5’AAATGTCCAAGGCACAAAGTG
*TP53BP1*	5’GGAACAGAAGGAGAAAGAGAAGG	5’CAGAACCCCAAAACCCAAC
*18S rRNA*	5’GCCCGAAGCGTTTACTTTGA	5’TCCATTATTCCTAGCTGCGGTATC
*GAPDH*	5’GAAACTGTGGCGTGATGGC	5’CACCACTGACACGTTGGCAG
*CCNB1* FOXM1 BS	5’ CGCGATCGCCCTGGAAACGCA	5’CCCAGCAGAAACCAACAGCCGT
*BRCA1* FOXM1 BS	5’CAAGGTACAATCAGAGGATGGG	5’TCCTCTTCCGTCTCTTTCCT
*RAD51* FOXM1 BS	5’ACCAGGCAGAGAATCTTGTTC	5’TTCAAGTCTAACCCAGTGCAG
*RAD51* MYC BS	5’GCTCCATTTCCCACTTCTATCC	5’CTTTTGGCACTTCTGGTCG
*BRCA1* MYC BS	5’ CTTCCAGTTGCGGCTTATTG	5’ GGGATTGGGACCTCTTCTTAC

### Real-time quantitative PCR (RT-qPCR)

Seventy-two hours after siRNA transfection, total RNA from the cells was extracted with the Trizol reagent (15596-028, Invitrogen) according to the manufacturer’s protocol. The cDNA was synthesized using SuperScript II reverse transcriptase (180604014, Invitrogen) with 1 μg of total RNA in a 20 μL reaction. The resulting cDNA was diluted 1:20 in nuclease-free water, and 1 μL was used per qPCR reaction in triplicate. qPCR was carried out using Power SYBR Green PCR Master Mix (4367659, Thermo Fisher Scientific) on a CFX384 Real-Time PCR Detection System (Bio-Rad), including a non-template negative control. Amplification of *GAPDH* or *18S rRNA* was used to normalize the level of mRNA expression. The sequences of the primer pairs were listed in Table 1.

### RNAi and gene expression analysis

*FOXM1*-specific siRNAs and scrambled negative control siRNAs were synthesized by Integrated DNA Technologies (IDT, Coralville, IA, USA). 3.5 × 10^5^ cells/well were seeded in 6-well plates and incubated at 37 °C overnight. The next day, 20 nM of each siRNA was transfected into the cells with Oligofectamine Transfection Reagent (12252011, Invitrogen) according to the manufacturer’s instructions. Culture media was added 6–8 h after transfection without washing the cells. Forty-eight h after siRNA transfection, cells were trypsinized and seeded in 6-well plates and kept overnight. Approximately 72 h after siRNA transfection, cells were treated with vehicle, 1 μM or 5 μM ( + )-JQ1 for 24 h before the Western blot analysis. To check the downregulation of *FOXM1* expression, the transfected cells were collected to extract total RNA for qRT-PCR or proteins for the Western blot analysis at 72 h after transfection. The sequences of siRNAs used are listed below:

*FOXM1* siRNA#1: sense rGrUrGrCrCrArArCrCrGrCrUrArCrUrUrGrArCrArUrUrGGA, antisense rUrCrCrArArUrGrUrCrArArGrUrArGrCrGrGrUrUrGrGrCrArCrUrG;

*FOXM1* siRNA#2: sense rGrCrGrCrUrArUrUrArGrArUrGrUrUrUrCrUrCrUrGrArUAA, antisense rUrUrArUrCrArGrArGrArArArCrArUrCrUrArArUrArGrCrGrCrArC.

*MYC* siRNA#1: sense rArUrCrArUrUrGrArGrCrCrArArArUrCrUrUrArArArArAAA, antisense rUrUrUrUrUrUrUrArArGrArUrUrUrGrGrCrUrCrArArUrGrArUrArU;

*MYC* siRNA#2: sense rCrGrArCrGrArGrArCrCrUrUrCrArUrCrArArArArArCrATC, antisense rGrArUrGrUrUrUrUrUrGrArUrGrArArGrGrUrCrUrCrGrUrCrGrUrC.

GIPZ Lentiviral FOXM1 shRNA1 (Clone Id: V2LHS_283849; RHS4430-200200806) and shRNA5 (Clone Id: V3LHS_396939; RHS4430-200247386) were purchased from Dharmacon (Horizon, Rivvity, Inc.).

### Cytotoxicity assay using sulforhodamine B (SRB) and drug synergy studies

SRB assays were performed as previously described^80,81^ with some modifications shown below. For OVCA420, OV90, and ES-2 cells, 3000 cells/well were seeded in 96-well plates and treated with drugs after 12 h of seeding. The cells were incubated for another 3 days. Dose-response curves and IC_50_ for each drug were determined by GraphPad Prism (ver. 6) with four parameters. All curves were constrained with 100% at the top. Synergy was determined by calculating the combination index (CI) obtained from plate reading. CI was calculated based on dividing the expected additive effect by the observed effect. N represents the total number of CIs determined from various dose-response combinations that produced 20–80% effect from independent experiments as previously described^82^. Additional synergy calculations were generated using Combenefit software using three classical Synergy models (Loewe, Bliss, and HAS)^83^.

### Colony formation assay

For OVCA420, OV90, and ES-2, 1000 cells were seeded in 6-well plates. For MDA-MB-436 rucaparib-resistant cells (RR-1 and RR-2), 2000 or 3000 cells per well were seeded in 6-well plates. The cells were treated with drugs after 12 h of seeding and further incubated for another 3 days before changing to fresh media. The medium was changed every 3 days to allow colonies to form. At the end of the experiments, colonies were stained with SRB and imaged with the Molecular Imager ChemiDoc MP System (Bio-Rad). Stained SRB was later dissolved and measured with a plate reader. The analysis of the clonogenic assay was performed in GraphPad Prism (ver. 6).

### High-content image analysis

For PARP1 trapping analysis, cells were treated with indicated doses for 24 h and pre-extracted with 0.2% Triton-X 100 in PBS for 2 min on ice as previously described^70^. High-content image analysis of trapped PARP1 and pH2AX was performed using fluorescence images, taken with a 20X objective using ImageXpress Pico (Molecular Devices), with CellReporterXpress software (v2.9.4.19394) (Molecular Devices). For trapped PARP1 analysis, positive and total cell counts from CellReporterXpress were converted to percent positive cells, and graphing and statistical analysis were performed using GraphPad Prism. For the phospho-H2AX (pH2AX) analysis, integrated fluorescence intensities for DAPI and pH2AX for each cell were exported as a matrix and processed, analyzed, and plotted using R packages. The R script used to process the pH2AX data was provided in the Supplementary Information.

### Statistical analysis

All data were analyzed using GraphPad Prism (ver. 6). Results were expressed as means ± standard error of the mean (S.E.M.) unless otherwise indicated. Differences between treatment regimens were analyzed by one-way ANOVA or two-tailed Student’s *t* test. *p* ≤ 0.05 was considered to be statistically significant, *p = 0.05, **p < 0.01, ***p < 0.001, ****p < 0.0001.

### TCGA dataset from C-bioportal

High-grade serous ovarian cancer (TCGA, GDC) data were assessed through the cbioportal website^84^, and mRNA expression in tpm *Z*-scores was selected as a genomic profile. The top and bottom quartile of gene expression was defined as EXP > = 0.675 (top quartile, Q1) and EXP < = -0.675 (bottom quartile, Q4), respectively. Significance in overall survival between the two groups was calculated using the log-rank test. Additional analysis of genomics alterations significantly associated with either expression group (Q1 vs Q4) was retrieved from cbioportal within the Groups analysis. Statistical methods for significant association between groups and genomic alterations (either mutations or copy number alterations) are two-sided Fisher Exact test (*p*-value) and Benjamini–Hochberg (*q*-value) as previously described^84^.

### Caris life sciences dataset

DNA and RNA next-generation sequencing (NGS): NGS was performed on genomic DNA isolated from formalin-fixed paraffin-embedded (FFPE) tumor samples using the NextSeq or NovaSeq 6000 platforms (Illumina, Inc., San Diego, CA). For NextSeq sequenced tumors, a custom-designed SureSelect XT assay was used to enrich 592 whole-gene targets (Agilent Technologies, Santa Clara, CA). For NovaSeq sequenced tumors, more than 700 clinically relevant genes were sequenced at high coverage.

For RNA sequencing, Qiagen RNA FFPE tissue extraction kit was used for extraction, and the RNA quality and quantity were determined using the Agilent TapeStation. Biotinylated RNA baits were hybridized to the synthesized and purified cDNA targets, and the bait-target complexes were amplified in a post capture PCR reaction. The Illumina NovaSeq 6500 was used to sequence the whole transcriptome from patients to an average of 60 M reads. Raw data was demultiplexed by Illumina Dragen BioIT accelerator, trimmed, counted, PCR-duplicates removed, and aligned to human reference genome hg19 by STAR aligner. For transcription counting, transcripts per million values were generated using the Salmon expression pipeline.

Real-world overall survival: Real-world overall survival information was obtained from insurance claims data and calculated from the first treatment time to the last contact. Hazard ratio (HR) was calculated using the Cox proportional hazards model, and *P* values were calculated using the log-rank test.

Compliance statement: This retrospective study was conducted under Caris Life Sciences’ Research Data Banking protocol, which was reviewed and granted IRB exemption by the WCG IRB. The study adhered to the ethical guidelines of the Declaration of Helsinki, the Belmont Report, and the U.S. Common Rule.

## Supplementary information

Supplementary Information

## Data Availability

The Cancer Genome Atlas data were accessed via cbioportal. Caris Genomics data were accessed through the Caris Precision Oncology Alliance.

## Code availability

R script used in the data analysis was provided in the Supplementary Information. TCGA data analysis used the cbioportal built-in tools for group comparison. Caris data analysis used built-in tools available from the Caris CODEai portal.
