# Supplementary material for: BET inhibition disrupts the FOXM1-MYC axis to induce BRCAness and enhance PARP inhibitor response
Source: NPJ Precis Oncol. 2026 Mar 12;10:209. doi: 10.1038/s41698-026-01360-x (PMC13249861; doi:10.1038/s41698-026-01360-x)
Supplement: Supplementary file 1 — Supplementary Information [file 41698_2026_1360_MOESM1_ESM.pdf]

Table S1: *Genes with overlapped binding sites for MYC and FOXM1 that are associated with Disease Ontology for Hereditary Breast and Ovarian Cancer*

**ATM**	(+196)	**CSTF1**	(+9)	HRAS	(-1795)	**PALB2**	(-76)	**SSBP1**	(-375)
**ATR**	(-18)	CYP1B1	(-883)	JUNB	(-8718)	PIK3CA	(-618)	STAT1	(-6201), (-54)
AURKA	(-108)	ECT2	(+13)	**KAT5**	(-113)	**PLK1**	(-17)	TGFB1	(-254)
**BABAM1**	(-6341)	EPHB2	(-6301)	CCAR2	(+2)	**PTEN**	(-668), (+451)	**TOP2A**	(-80)
**BLM**	(+45)	**ERCC1**	(-4225), (-388)	KIF4A	(-42)	PTK2	(-1194)	**TOPBP1**	(-60)
**BRIP1**	(-36)	**ERCC5**	(+984)	**MDC1**	(-4960), (+268)	**RAD50**	(+76)	TSG101	(+276)
**EMSY**	(-2320)	ESR2	(-676)	**MLH1**	(+241)	**RAD51B**	(-2999)	UBE2I	(+382)
CASP10	(+160)	EZH2	(-322)	MYC	(-1974), (+198)	**RAD51D**	(-78)	UBE2L6	(-541)
CASP8	(+408)	**FAM175A**	(+509)	**NBN**	(+380)	**RAD52**	(+4)	**UBE2T**	(-96)
CCNT1	(+209)	**FANCD2**	(+4)	NCL	(+309)	RELA	(-150)	UBXN1	(+11)
CDC27	(-29)	FOXC1	(-5911)	NCOA3	(-103)	**RFC1**	(+20)	**XPC**	(-34)
CDK2	(+79)	GTF2I	(-270)	NME1	(-45)	RNASEL	(-6598)	XPO1	(-131)
CDKN1A	(+70)	**H2AFX**	(-6458), (-189)	NPM1	(+885)	**SMC1A**	(+274)	**XRCC3**	(-3393)
**CHEK1**	(-511)	HDAC1	(-191)	NR1H3	(-9138)	SP1	(-8843)	ZNF350	(-3)
COL1A1	(+928)	HMMR	(-110)	NUMB	(-307)				

GREAT version 3.0.0 (Species assembly: hg19). Association rule: Basal+extension; 10000 bp upstream, 1000 bp downstream, 0 bp max extension, curated regulatory domains included. DNA repair genes are highlighted in bold. Values in parenthesis are distance in nucleotide base for overlapping binding sites for FOXM1 and MYC relative to transcription start site (TSS). Negative values represent upstream from TSS. Positive values represent downstream from TSS.

A	B	Neither	A Not B	B Not A	Both	Log2 Odds Ratio	p-Value	q-Value	Tendency
BRD4: Top25%	FOXM1: Top25%	241	46	58	34	1.619	<0.001	<0.001	Co-occurrence
BRD4: Top25%	MYC: Top25%	229	55	70	25	0.572	0.191	0.286	Co-occurrence
FOXM1: Top25%	MYC: Top25%	216	68	71	24	0.103	0.784	0.784	Co-occurrence

Table S2. *Significant co-occurrence between high BRD4 and high FOXM1 expressions in HGSC from TCGA dataset.*

	Group 1	Group 2
CCNE1 AMPLIFICATION (N = 2089)	CCNE1 Amplified (N = 210)	CCNE1 Not Amplified (N = 1879)
High BRD4 VS. Low BRD4 (N = 2988)	BRD4-top25%-stdRx (N = 1481)	BRD4-bottom25%-stdRx (N = 1507)
FOXM1-top25% (N = 2988)	FOXM1-top25% (N = 760)	NOT FOXM1-top25% (N = 2228)
RB1 MUTATION (N = 2129)	RB1 Mutated (N = 57)	RB1 Not Mutated (N = 2072)
ETHNICITY (N = 2751)	Not Hispanic or Latino (N = 2322)	Unknown, Hispanic or Latino (N = 429)
MYC-top25% (N = 2988)	MYC-top25% (N = 720)	NOT MYC-top25% (N = 2268)
RACE (N = 2751)	White (N = 2019)	Asian or Pacific Islander, Unknown, Black or African American, Other (N = 732)

	Hazard Ratio	CI - Lower 95%	CI - Upper 95%	p
CCNE1 AMPLIFICATION	1.1814674807	0.964885361	1.44666451	0.1065295648
High BRD4 VS. Low BRD4	1.1347369292	1.0005824319	1.2868783793	**0.0489491532**
FOXM1-top25%	1.0501593758	0.9223704605	1.1956526818	0.4597245674
RB1 MUTATION	1.0233747359	0.7164704035	1.4617433532	0.8989229874
ETHNICITY	1.0021175499	0.8724621884	1.1510408097	0.9761280962
MYC-top25%	0.9532479303	0.836564665	1.0862060695	0.4723161418
RACE	0.8836903902	0.7826085333	0.9978279977	**0.046037757**

Table S3. *Prognostic factors included in the multivariate analysis for the overall survival in the Caris dataset.*

A	B	Neither	A Not B	B Not A	Both	Log2 Odds Ratio	p-Value	q-Value	Tendency
NOTCH3: AMP	CC2D1A: AMP	307	5	9	44	>3	<0.001	<0.001	Co-occurrence
BRD4: Top25%	NOTCH3: AMP	272	44	16	33	>3	<0.001	<0.001	Co-occurrence
BRD4: Top25%	CC2D1A: AMP	266	46	22	31	>3	<0.001	<0.001	Co-occurrence

Table S4: *Co-occurrence analysis of NOTCH3, CC2D1A, and BRD4.*

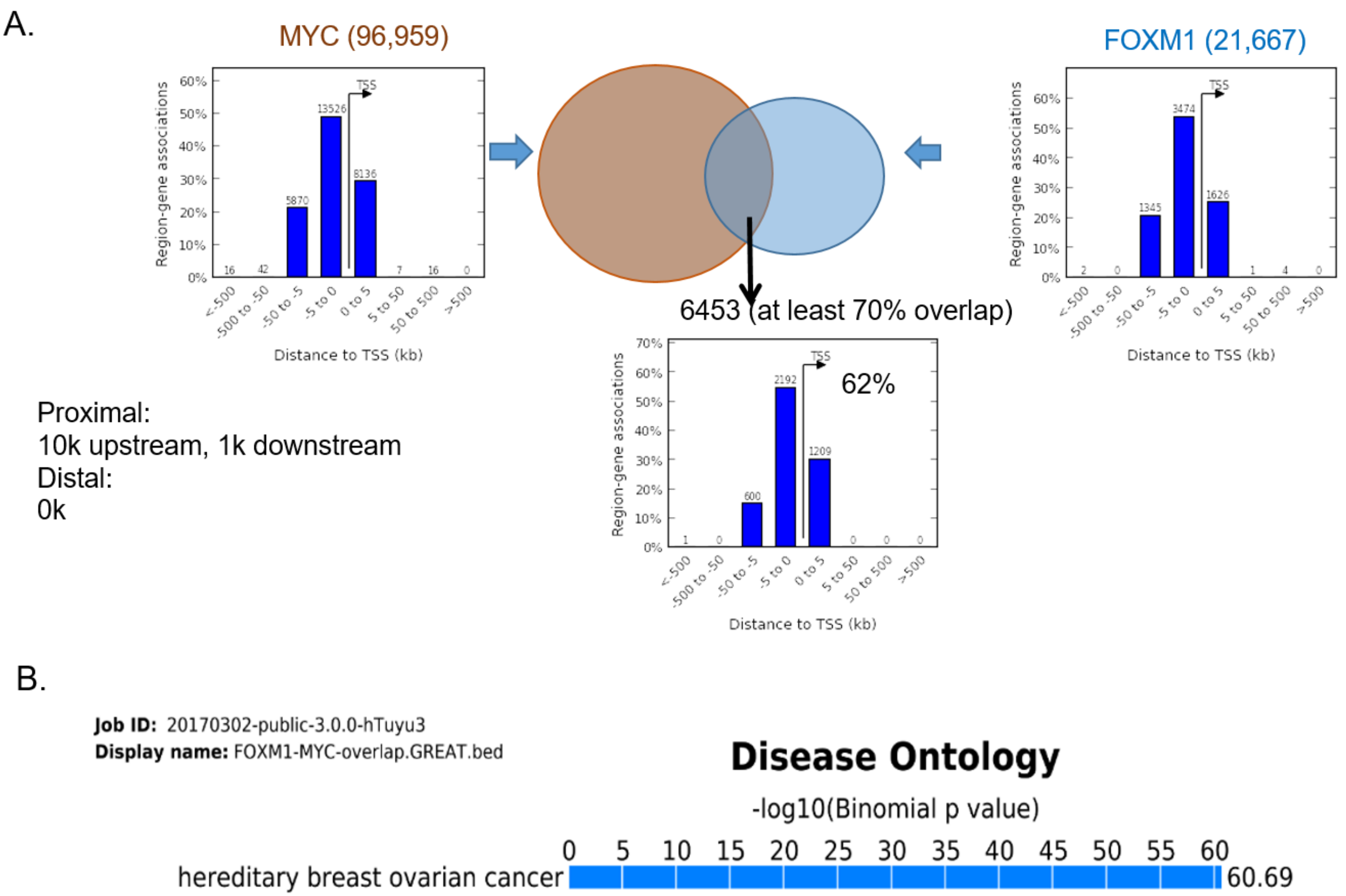

Figure S1: *FOXM1 and MYC co-occupy regulatory regions of DNA repair genes.* **(A)** Genome-wide ENCODE ChIP-seq analysis identifies 6,453 regions with ≥70% overlap between FOXM1 and MYC binding sites, with 62% positioned within proximal regulatory regions (-10 kb/+1 kb from TSS), implicating a potential co-regulation. **(B)** Disease ontology enrichment identifies "hereditary breast and ovarian cancer", linking co-occupied sites to relevant disease phenotypes. Annotation of genes in this disease ontology reveals enrichment of DNA repair genes, underscoring a possible transcriptional nexus between FOXM1 and MYC in regulating DNA repair pathway genes.

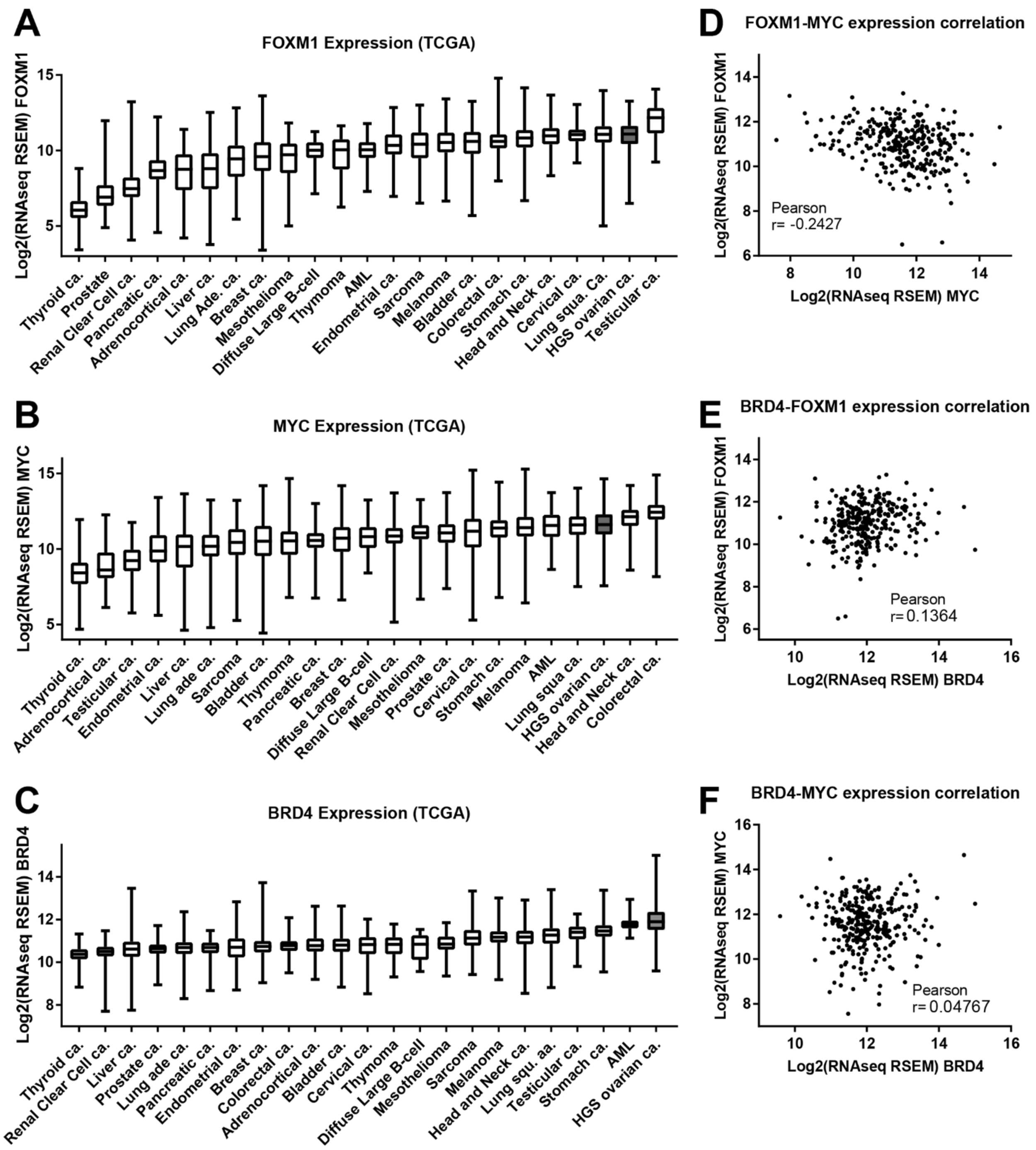

Figure S2: *Ovarian Carcinomas exhibit high FOXM1, MYC, and BRD4 mRNA expression with BRD4 positively correlated to FOXM1 and MYC*. **(A-C)** TCGA analyses show *FOXM1* (second highest), *MYC* (third highest), and *BRD4* (highest) median expression in ovarian cancer relative to other tumor types. **(D-F)** *FOXM1* and *MYC* are negatively correlated; BRD4 correlates positively with FOXM1, and more weakly with MYC, supporting BRD4-dependent regulation of FOXM1 and MYC transcription programs.

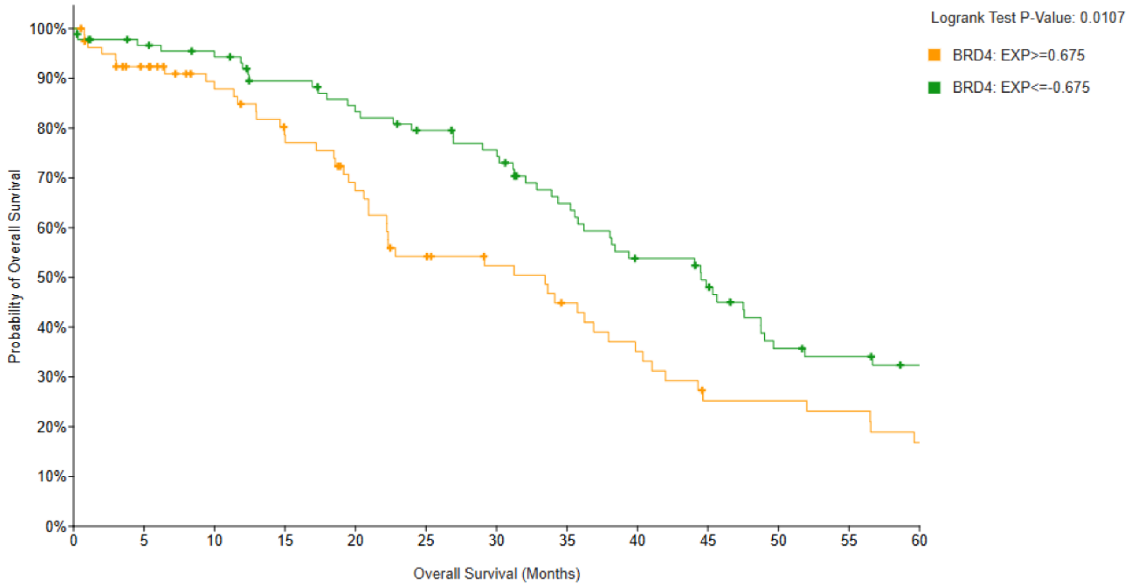

Number at risk (n)													
BRD4: EXP>=0.675	80	68	58	50	41	32	28	23	18	12	12	11	8
BRD4: EXP<=-0.675	90	84	80	73	67	62	58	47	38	33	23	21	18

Survival plot summary

	Number of Cases, Total	Number of Events	Median Months Overall (95% CI)
BRD4: EXP>=0.675	**80**	**52**	**33.44 (22.21 - 40.37)**
BRD4: EXP<=-0.675	**90**	**57**	**44.51 (36.20 - 49.64)**

Figure S3A. *High BRD4 expression is associated with poor overall survival in HGSC.* TCGA analysis shows BRD4-high patients (top quartile; mRNA Z-scores ≥ 0.675) have significantly shorter overall survival compared to those in the bottom quartile (mRNA Z-score ≤ -0.675). The log-rank test was used to test the null hypothesis that there is no difference in survival between top quartile and bottom quartile of BRD4 expression. P-value = 0.0107 (Log-rank test).

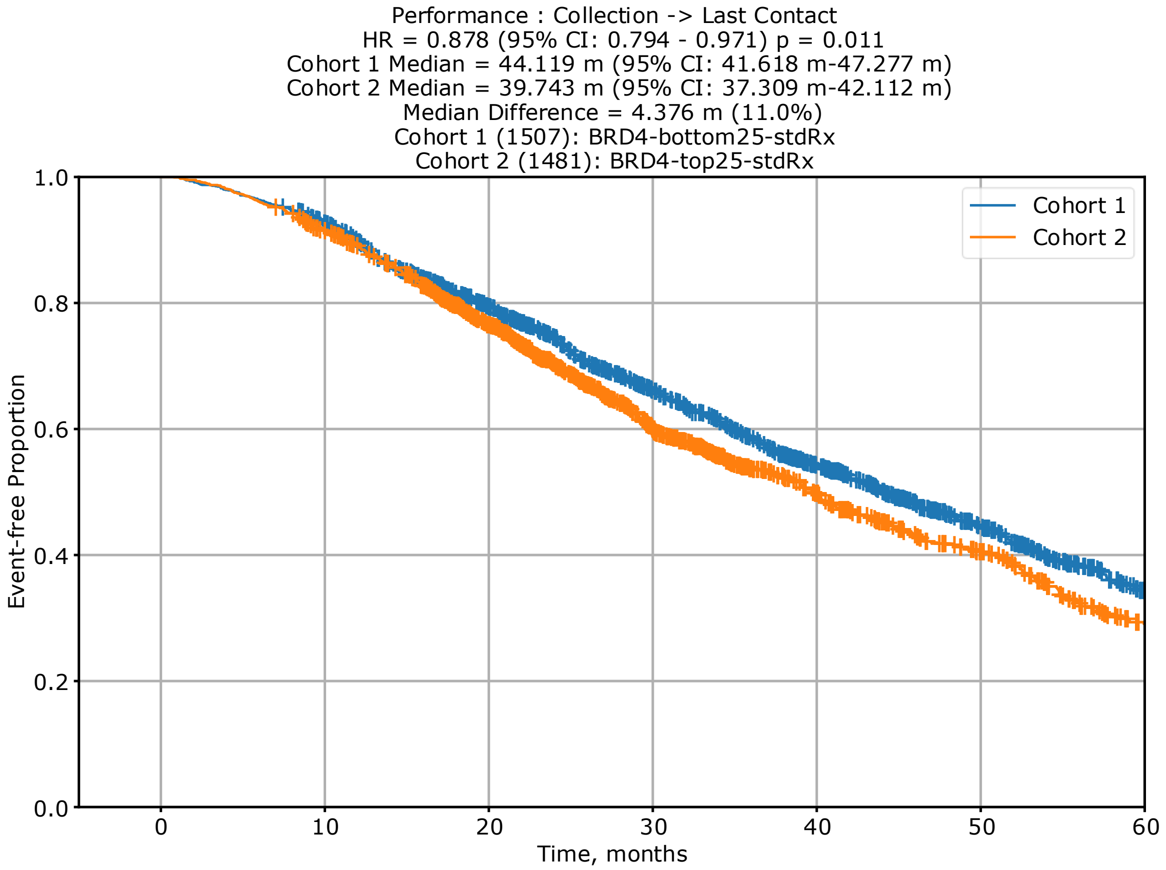

Cohort 1	1507	1381	1075	755	525	312
Cohort 2	1481	1341	988	585	332	214

Figure S3B. *High BRD4 expression is associated with poor overall survival in HGSC.* Caris cohort confirms this association in HGSC under standard chemotherapy. The log-rank test was used to test the null hypothesis that there is no difference in survival between top quartile and bottom quartile of BRD4 expression. Cohort 1: BRD4 expression at bottom quartile. Cohort 2: BRD4 expression at top quartile. P-value = 0.011 (Log-rank test).

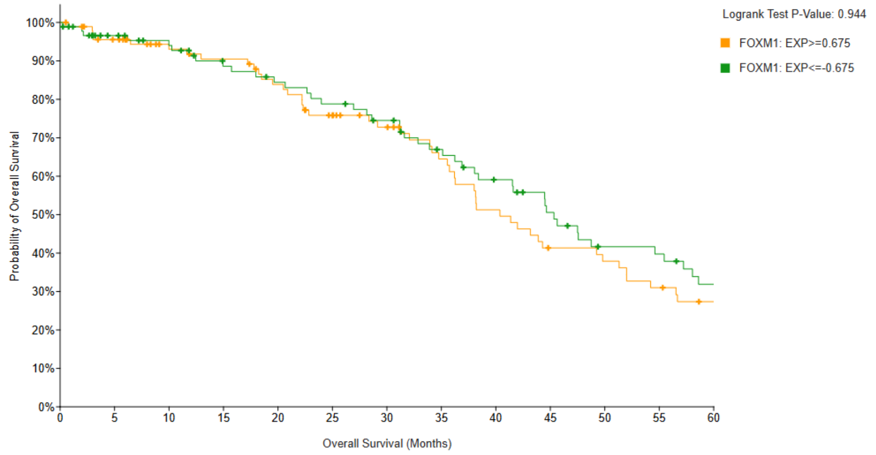

Number at risk (n)													
FOXM1: EXP>=0.675	92	83	73	70	63	54	47	39	31	24	22	18	14
FOXM1: EXP<=-0.675	89	78	72	64	60	56	51	43	36	29	22	21	16

Survival plot summary

	Number of Cases, Total	Number of Events	Median Months Overall (95% CI)
FOXM1: EXP>=0.675	**92**	**55**	**40.37 (36.20 - 52.00)**
FOXM1: EXP<=-0.675	**89**	**54**	**45.34 (38.40 - 58.05)**

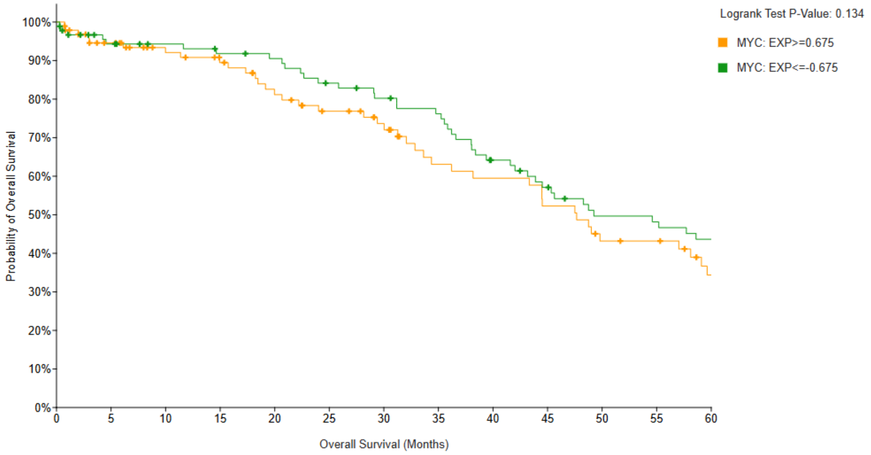

Number at risk (n)													
MYC: EXP>=0.675	94	82	72	67	58	51	45	35	33	29	23	22	15
MYC: EXP<=-0.675	90	80	76	73	71	65	61	57	46	40	33	32	29

Survival plot summary

	Number of Cases, Total	Number of Events	Median Months Overall (95% CI)
MYC: EXP>=0.675	**94**	**48**	**47.67 (38.17 - 59.63)**
MYC: EXP<=-0.675	**90**	**56**	**49.24 (43.17 - 77.04)**

Figure S3C-D: *Overall survival analysis of patients with high or low FOXM1 or MYC expression in the tumors from TCGA dataset.*

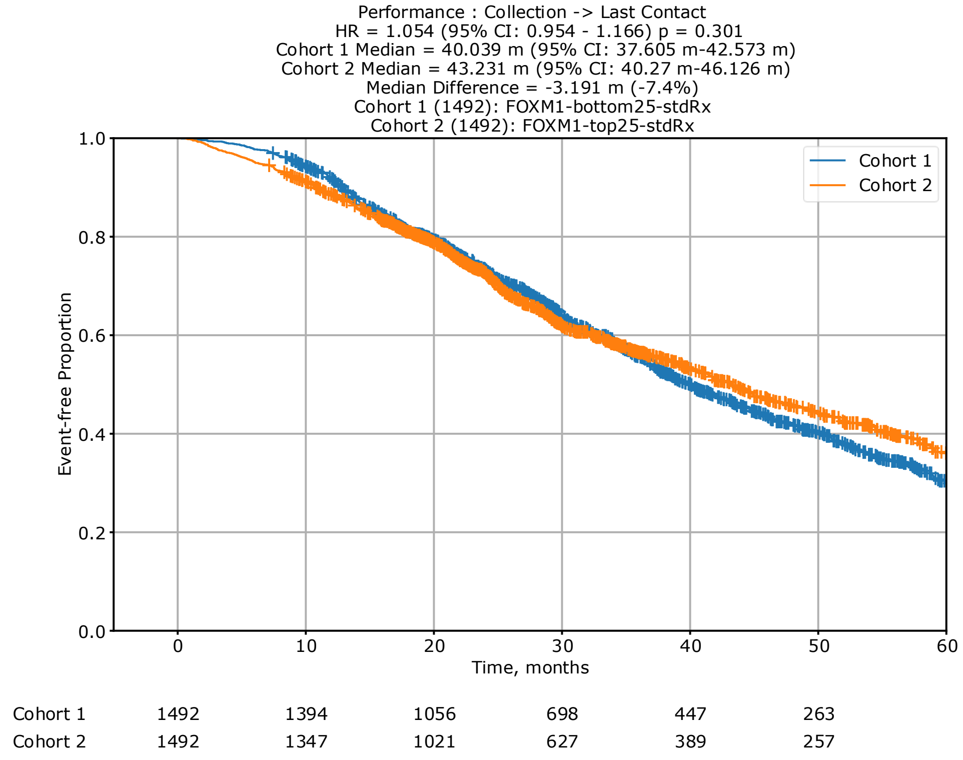

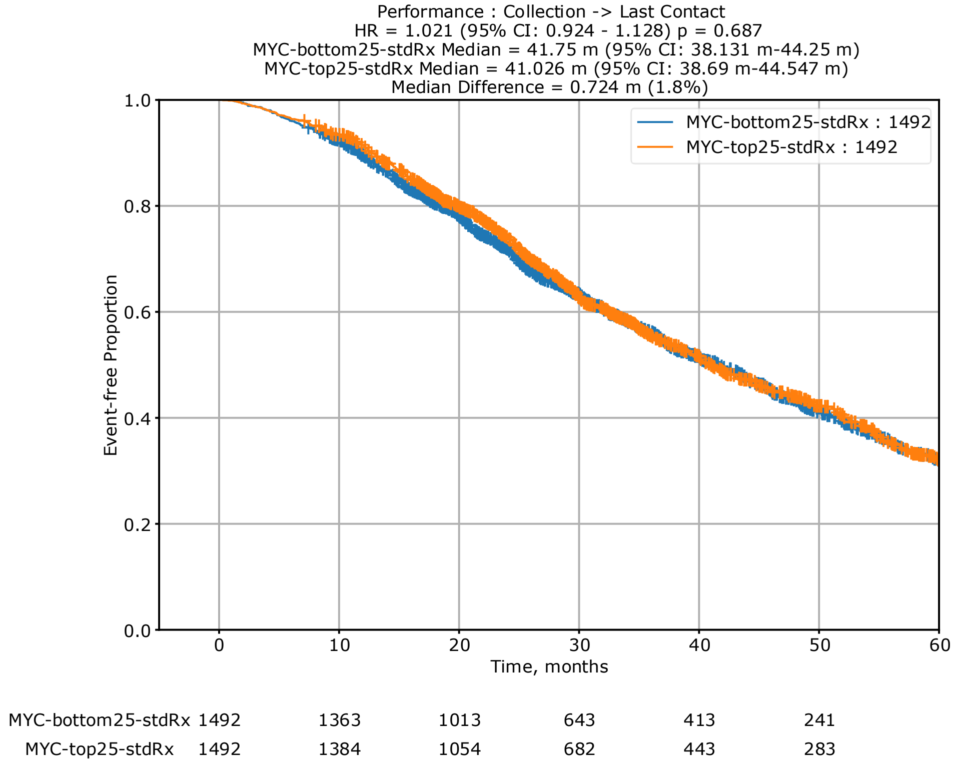

Figure S3E-F: *Overall survival analysis of patients with high or low FOXM1 or MYC expressions in the tumors from the Caris dataset.* Patients with high-grade serous ovarian cancer who are treated with standard chemotherapy (stdRx) consisting of carboplatin and paclitaxel are included in this analysis.

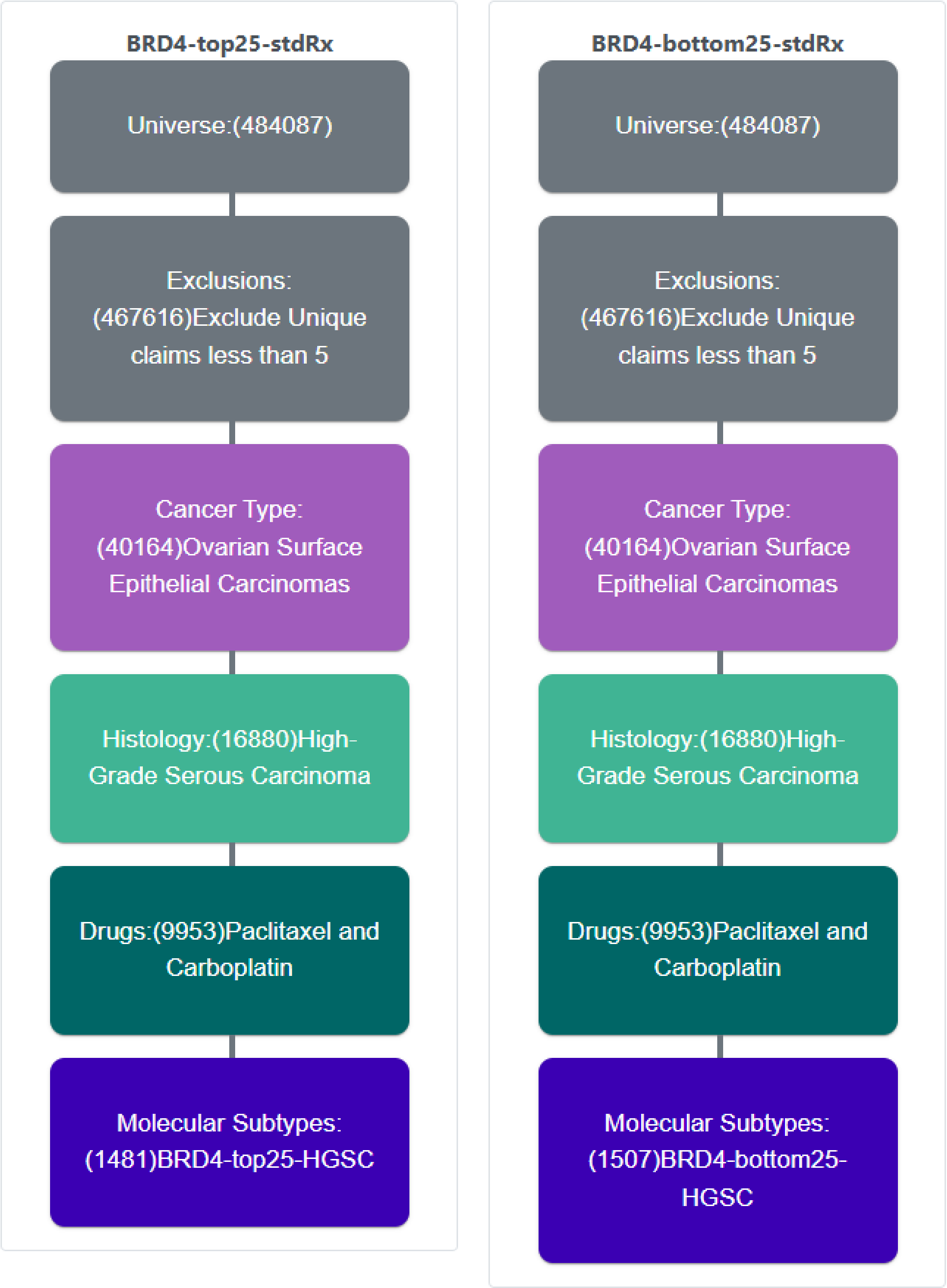

Figure S3G: *Cohort description for patients with high vs low BRD4 mRNA expressions in the tumor samples from the Caris dataset.*

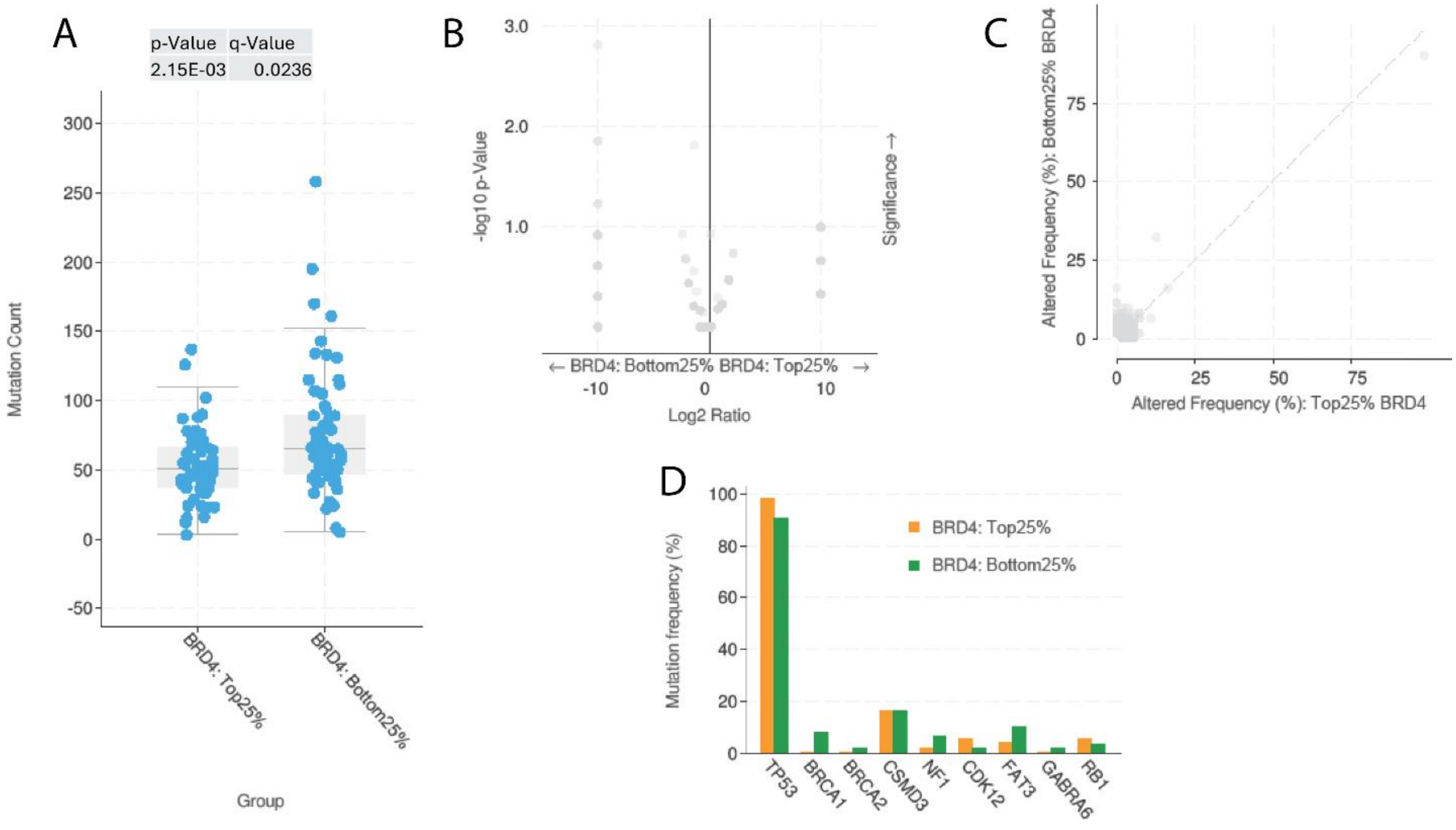

Figure S4A-D. *Mutation profiles associated with high vs. low BRD4 expression in TCGA HGSC tumors*
Figure S4A–D show the mutation landscape in high-grade serous ovarian cancer (HGSC) tumors stratified by BRD4 mRNA expression levels. Tumor samples were divided into high BRD4 (top 25%) and low BRD4 (bottom 25%) expression groups. These panels depict (A) total mutation counts per tumor, (B–D) the distribution and frequency of mutated genes enriched in either high- or low-BRD4–expressing tumors. This analysis highlights genetic alterations that significantly differ between BRD4 expression strata, providing insight into mutation patterns associated with BRD4-high tumors.

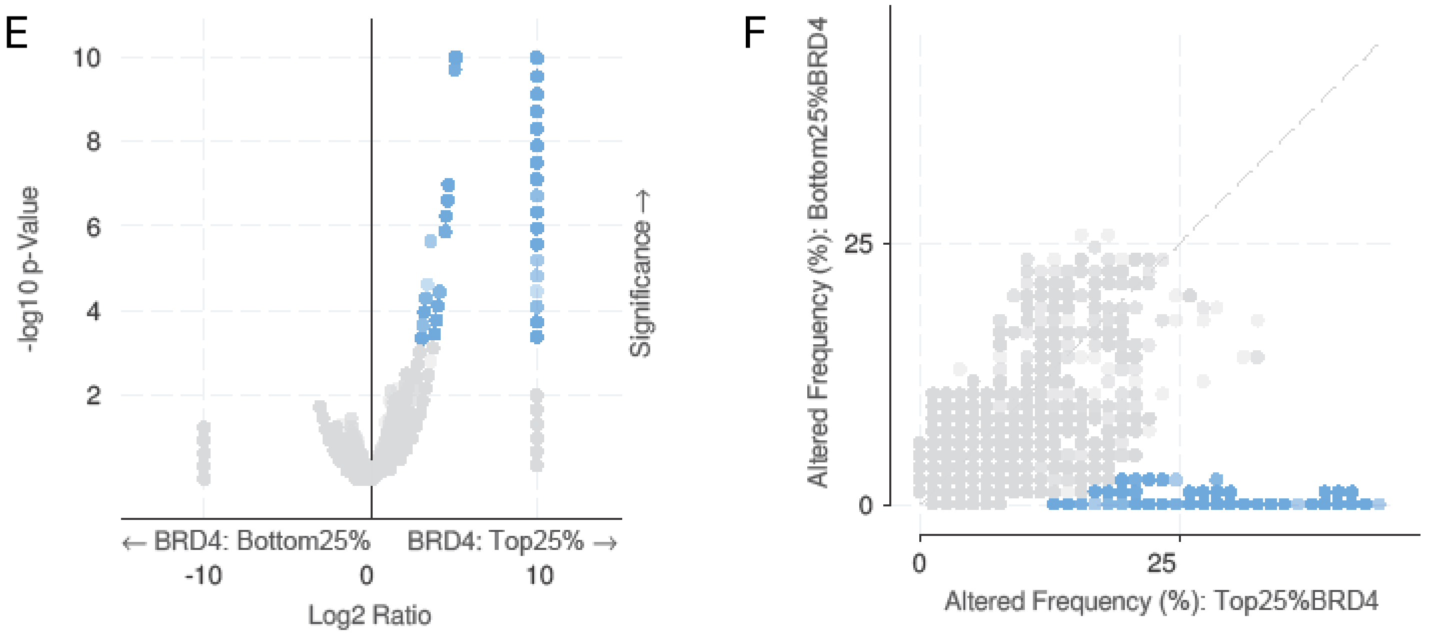

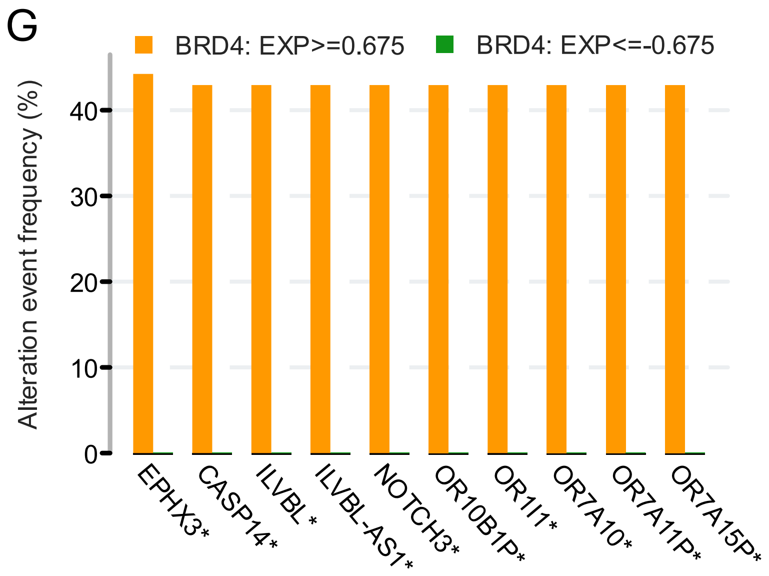

Figure S4E-G. *Copy number alterations significantly co-occurring with high BRD4 mRNA expression.* **(E)** Volcano plot indicating significant CNAs with high occurrence in samples with high BRD4 expression. **(F)** Significant CNAs with higher frequency of occurrence in samples with high BRD4 expression. **(G)** Frequency of alterations in neighboring genes in high BRD4 samples vs low BRD4 samples.

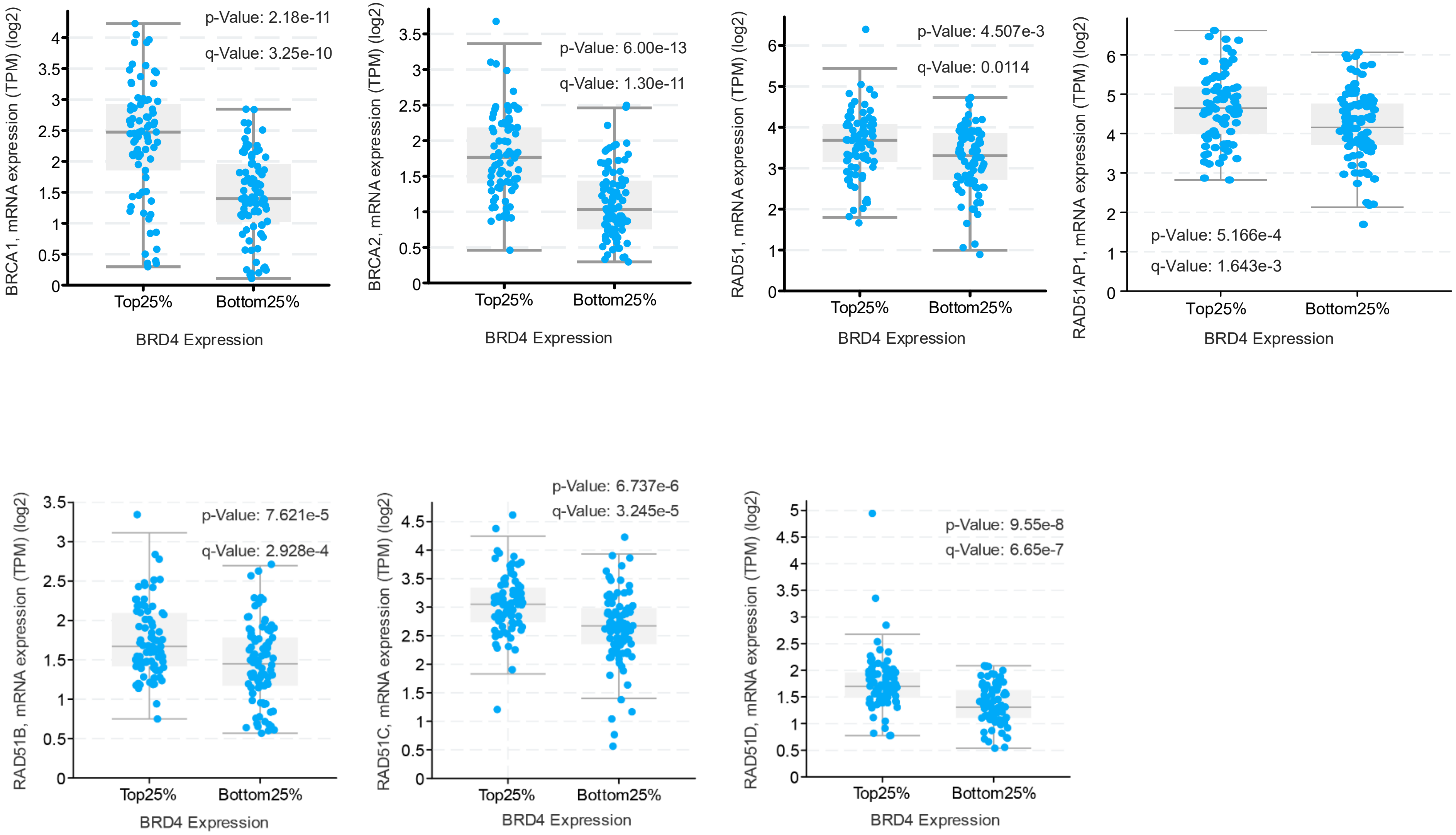

Figure S5A. *Expression of HR genes in tumors with either high (top25%) or low (bottom25%) BRD4 expression in TCGA dataset.*

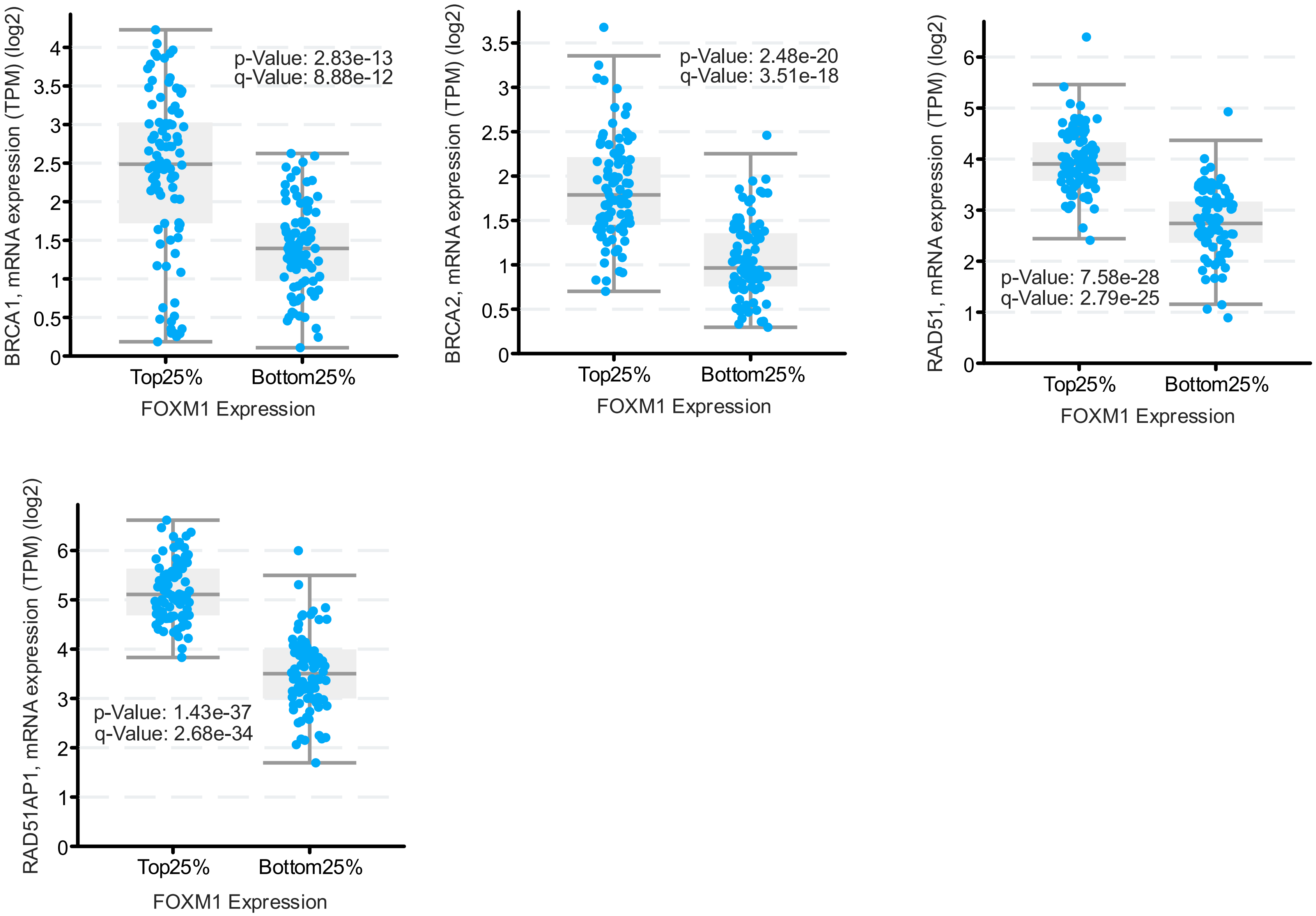

Figure S5B. *Expression of select genes in HR pathways in tumors samples with either high or low FOXM1 expression in TCGA dataset.*

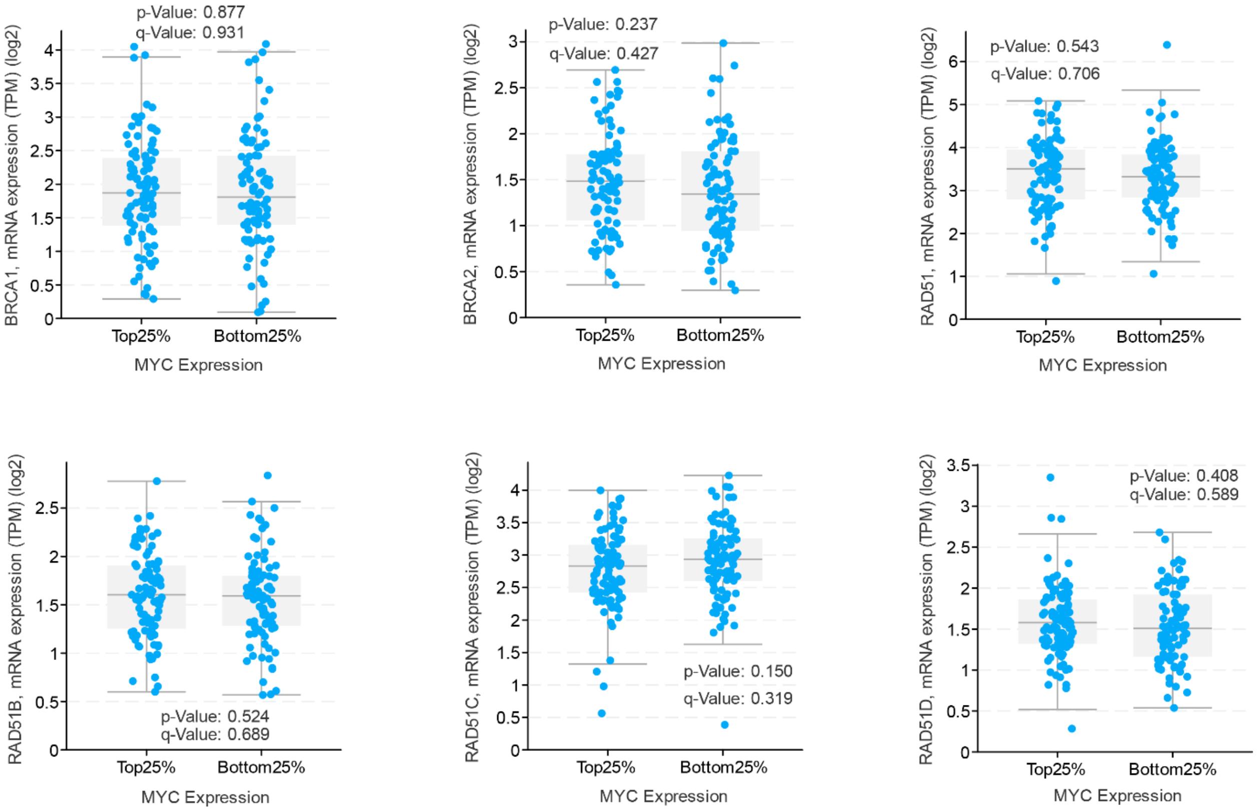

Figure S5C. *Expression of select genes in HR pathways in tumors samples with either high or low MYC expression in TCGA dataset.*

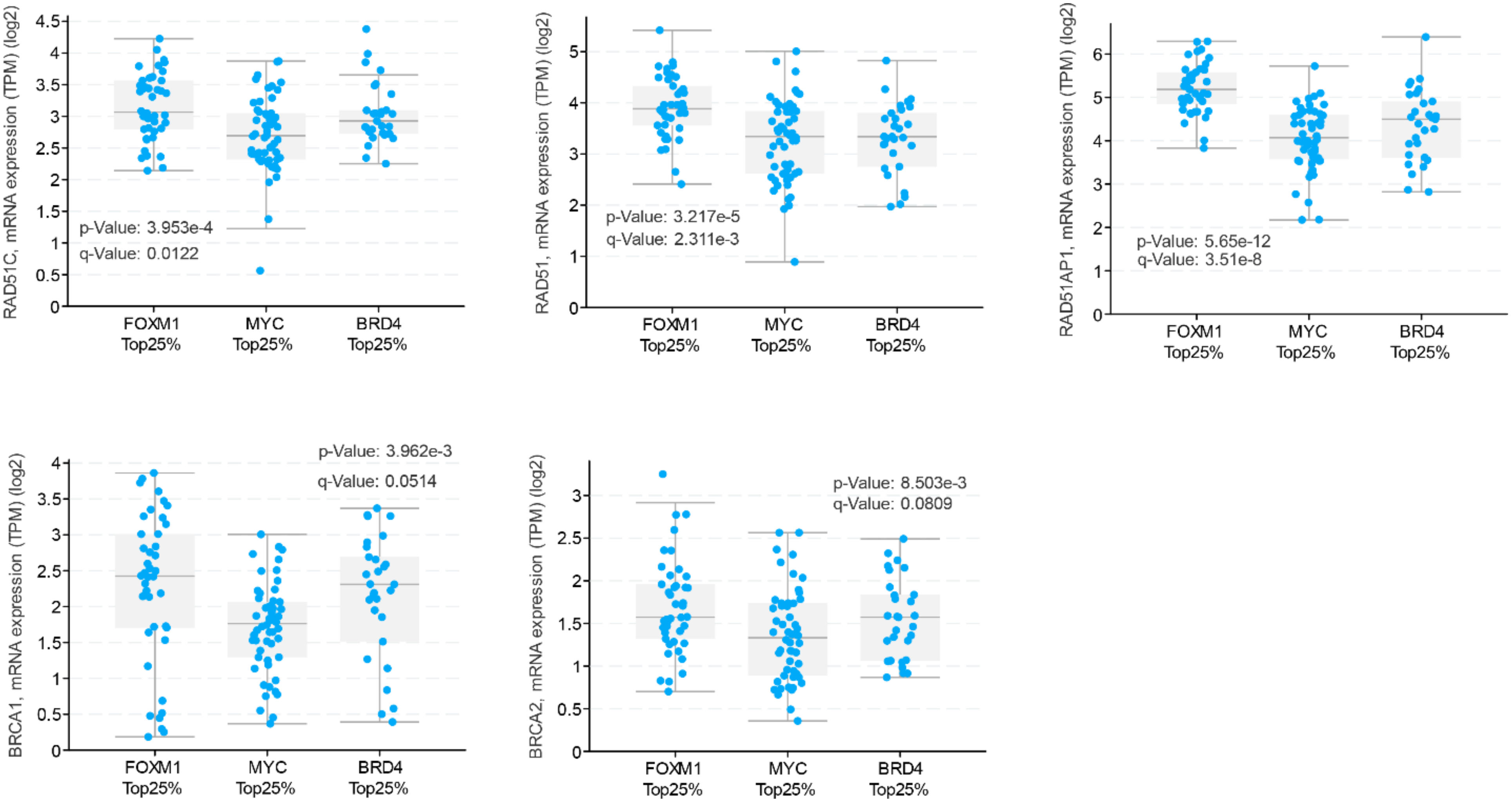

Figure S5D. *Expression of selected genes in HR pathways in tumor samples with either high FOXM1, MYC, or BRD4 expression in TCGA dataset. Tumors with overlapping expressions of high FOXM1, MYC, or BRD4 were excluded.*

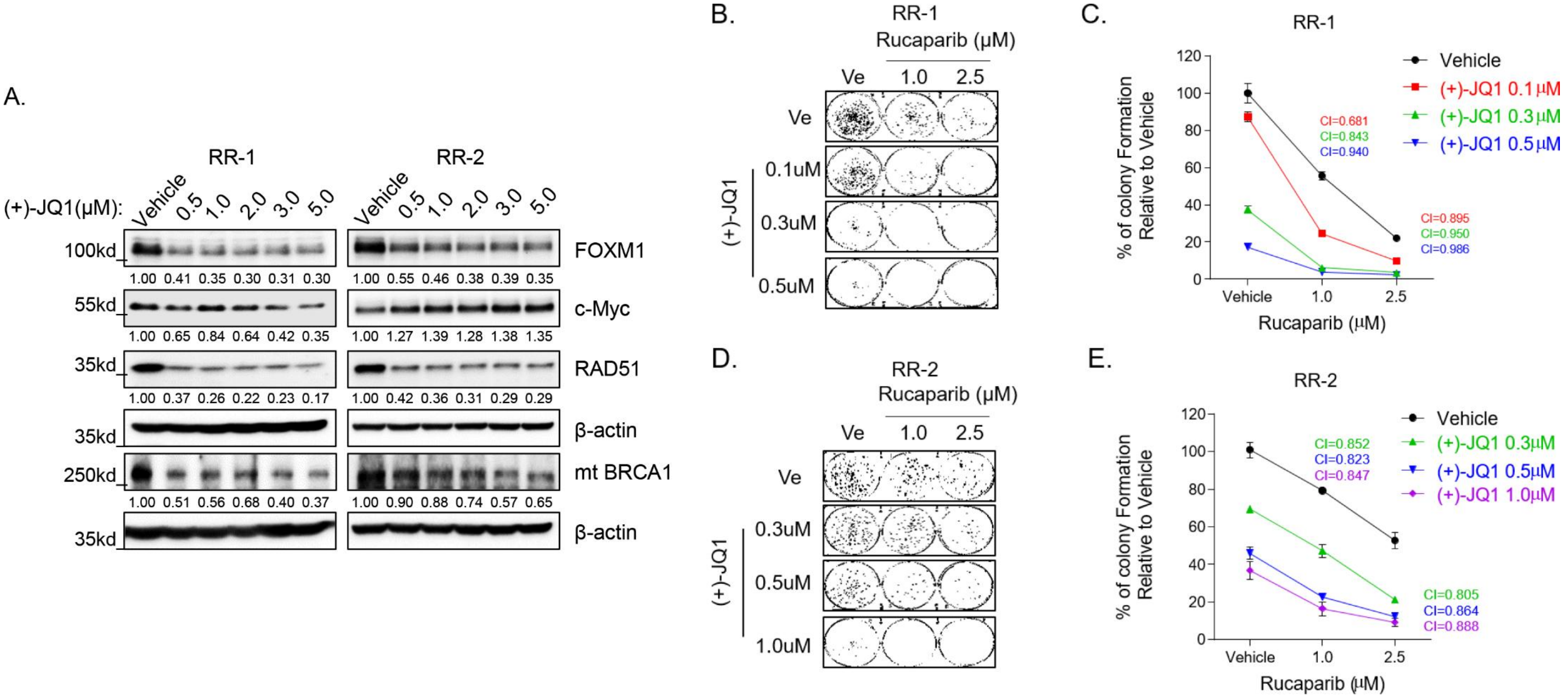

Figure S6. *BET inhibition with (+)-JQ1 restores PARPi sensitivity in rucaparib-resistant breast cancer cells by suppressing FOXM1/MYC and destabilizing mutant BRCA1.* **(A)** Western blot analysis shows (+)-JQ1 treatment decreases FOXM1 and RAD51 in both resistant clones (RR-1 and RR-2), while MYC downregulation occurs only in RR-1. Notably, mutant BRCA1 (mtBRCA1), which mediates acquired resistance by stabilizing its expression through HSP90, is markedly reduced following (+)-JQ1 exposure, suggesting transcriptional suppression of compensatory HR drivers. **(B–E)** Colony formation assays demonstrate dose-dependent growth inhibition by (+)-JQ1 and complete ablation of colonies when combined with sub-lethal rucaparib doses, indicating functional HR re-impairment and moderate synergy. These findings highlight BET inhibition as a strategy to overcome acquired PARPi resistance by targeting FOXM1–MYC signaling and destabilizing mtBRCA1. Data shown as mean ± S.E.M. is indicated in the graph.

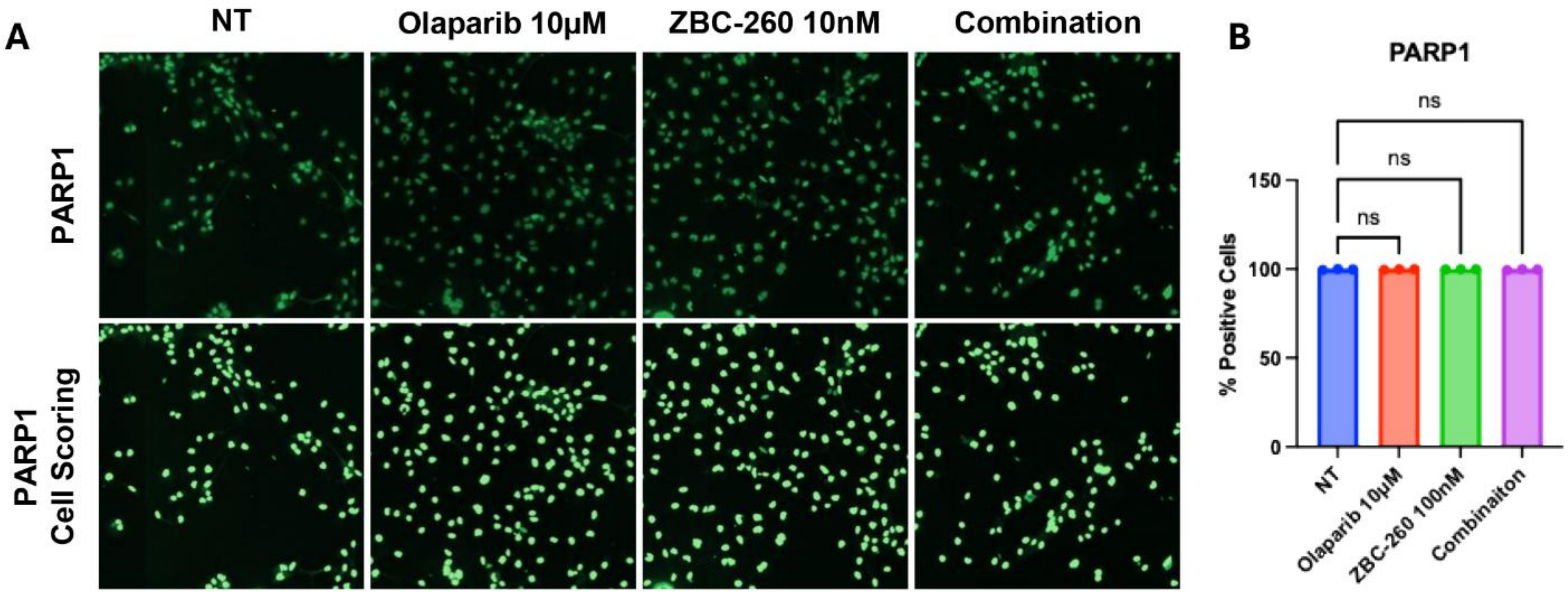

Figure S7. *Immunofluorescence of PARP1 and pH2AX in OVCA420 cells treated with Olaparib and ZBC260 as single agents and in combination.* **(A)** Representative images of immunofluorescent staining against PARP1 without pre-extraction. **(B)** Cell scoring for PARP1 positive (green) and negative (red) cells as determined by high-content image analysis with the CellReporterXpress from Molecular Devices (v2.9.4.19394). All data shown are as mean (SEM). Statistical analysis was performed using an ordinary one-way ANOVA with multiple comparisons to the NT (non-treated) group. ns = non-significant

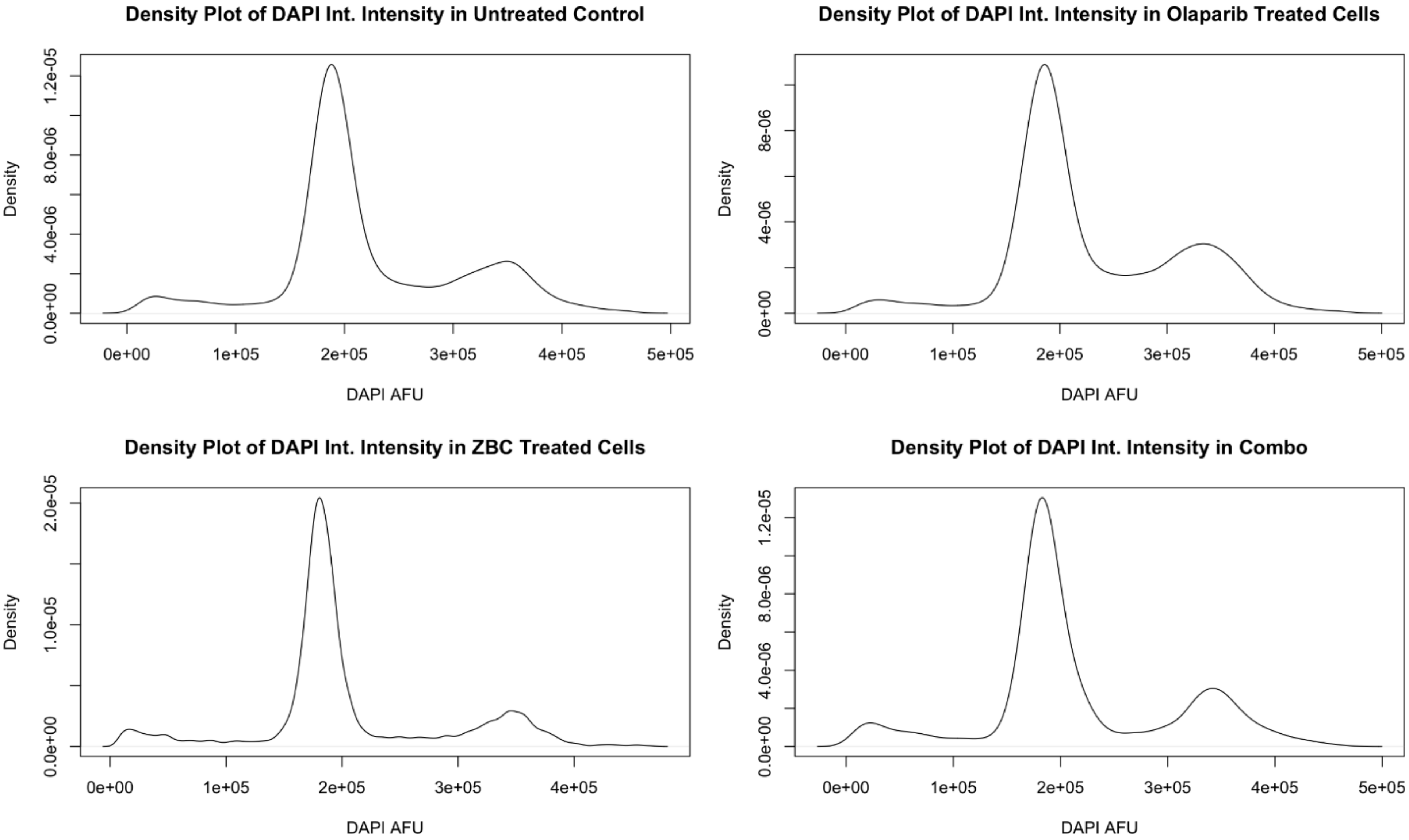

Figure S8. *High-content image analysis of DAPI nuclear staining intensity produces cell cycle profiles similar to flow cytometry.*

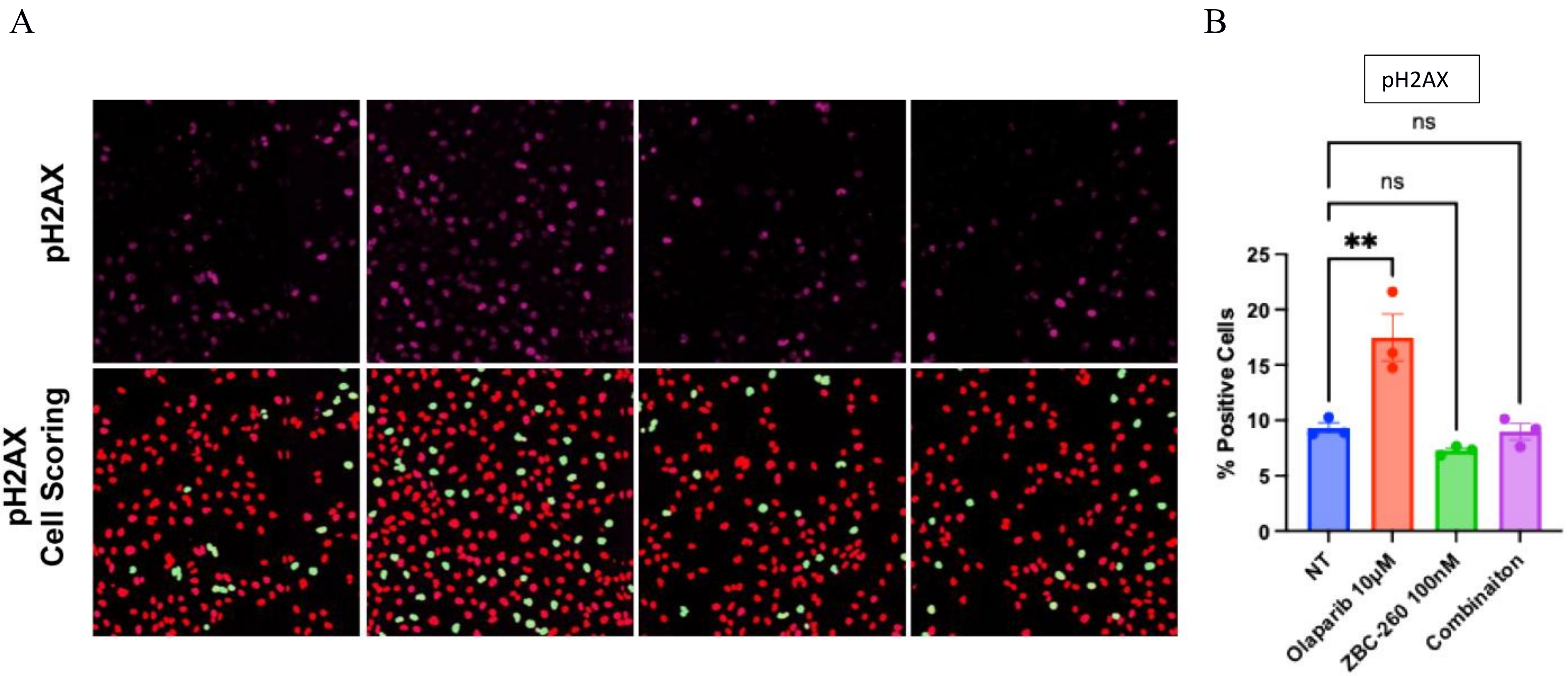

Figure S9. *Immunofluorescence analysis of pH2AX in OVCA420 cells following treatment.* **(A)** Representative images of immunofluorescent staining against pH2AX. **(B)** Cell scoring for pH2AX positive (green) and negative (red) cells as determined by high-content image analysis with the CellReporterXpress from Molecular Devices (v2.9.4.19394). Summary data of PARP1 staining includes analysis of >990 cells/replicate. Summary data of pH2AX staining includes analysis of >1200 cells/replicate. All data shown are as mean (SEM). Statistical analysis was performed using an ordinary one-way ANOVA with multiple comparisons to the NT (non-treated) group. ns = non-significant, **p<0.005.

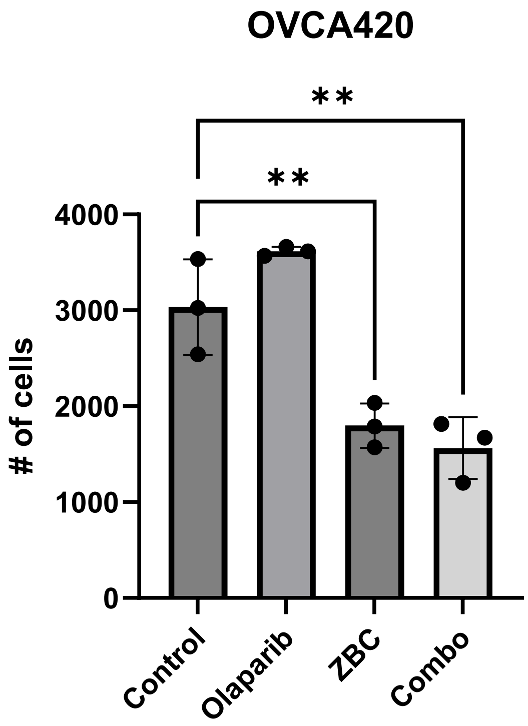

Figure S10. *High-content image analysis of remaining cells after treatment*. **p<0.005. Statistical analysis was performed using an ordinary one-way ANOVA with multiple comparisons to the control group.

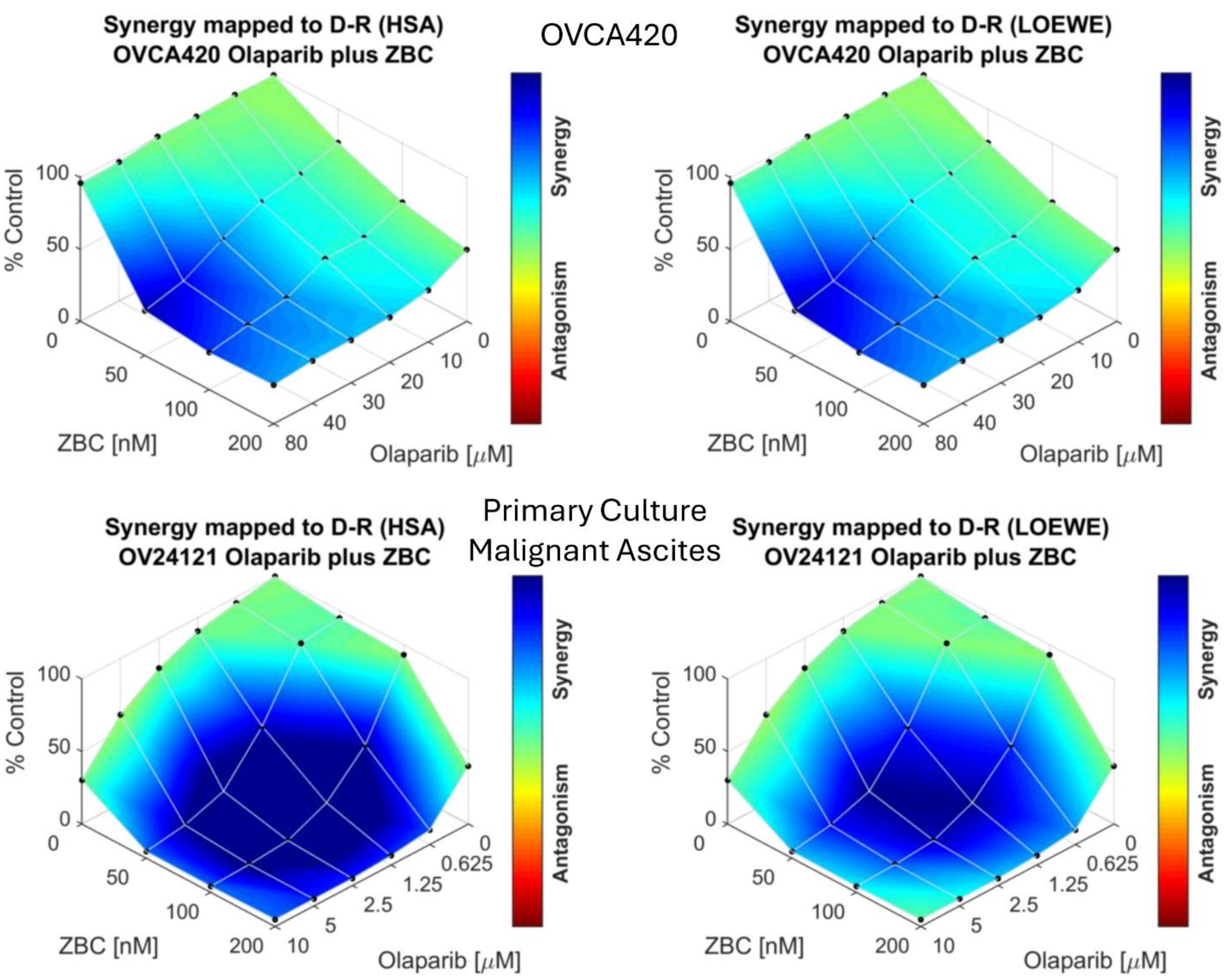

Figure S11. *Synergies between ZBC260 and olaparib in OVCA420 and OV24121 using HSA and LOEWE models with Combenefit software.*

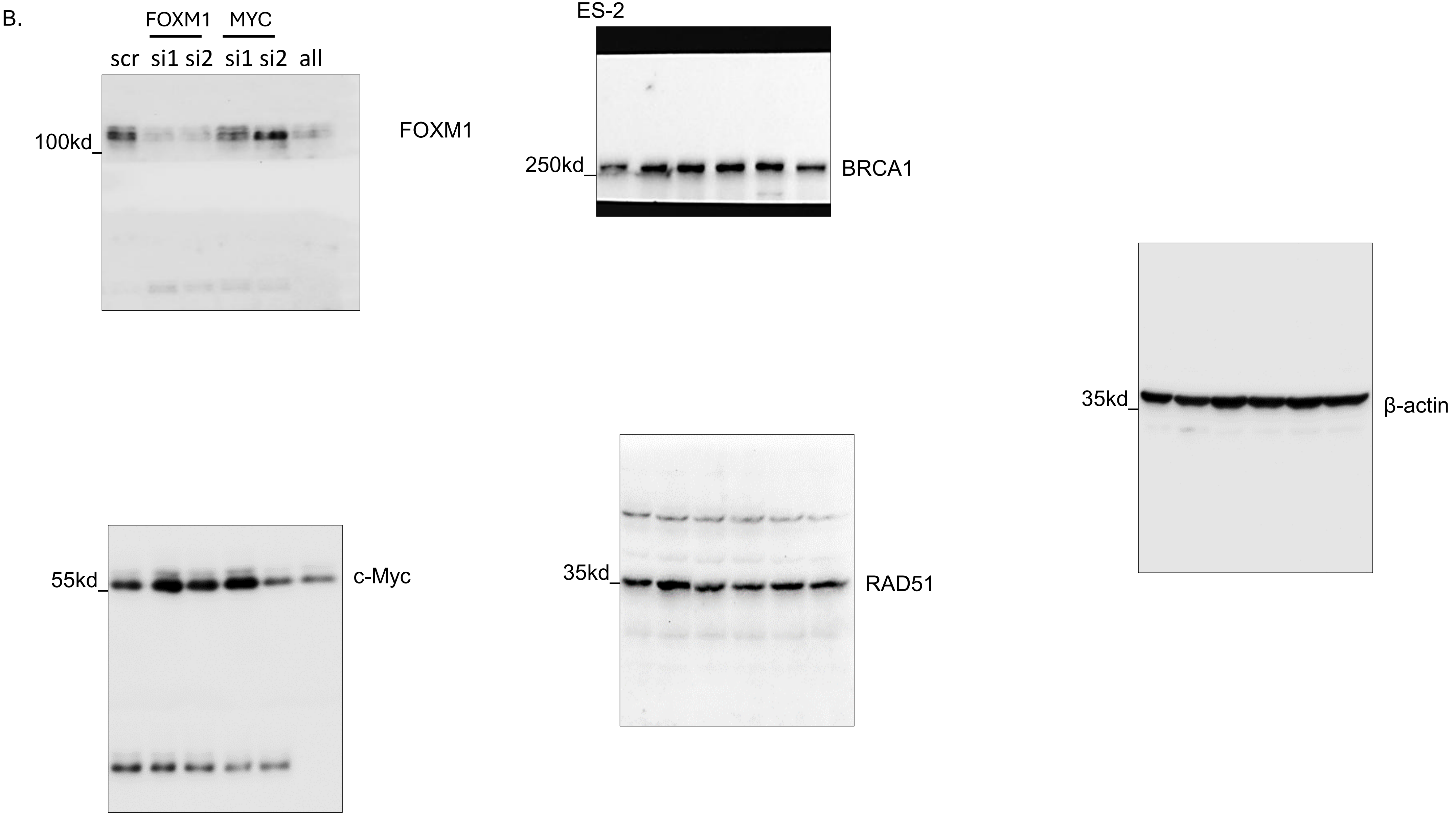
B.
FOXM1
MYC
scr
si1
si2
si1
si2
all
100kd
FOXM1
ES-2
250kd
BRCA1
35kd
β-actin
55kd
c-Myc
35kd
RAD51

Figure 1

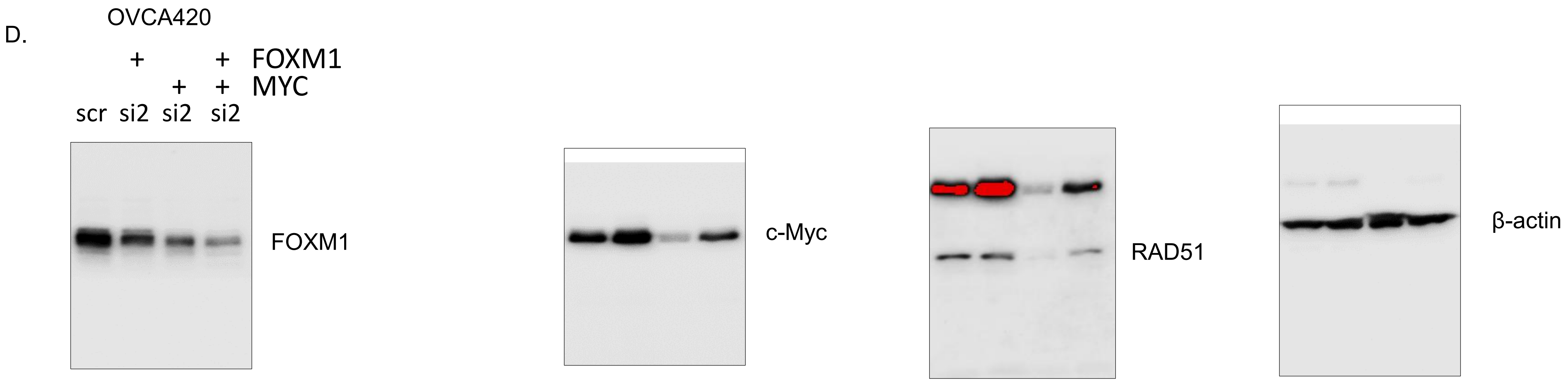

Figure 1

A

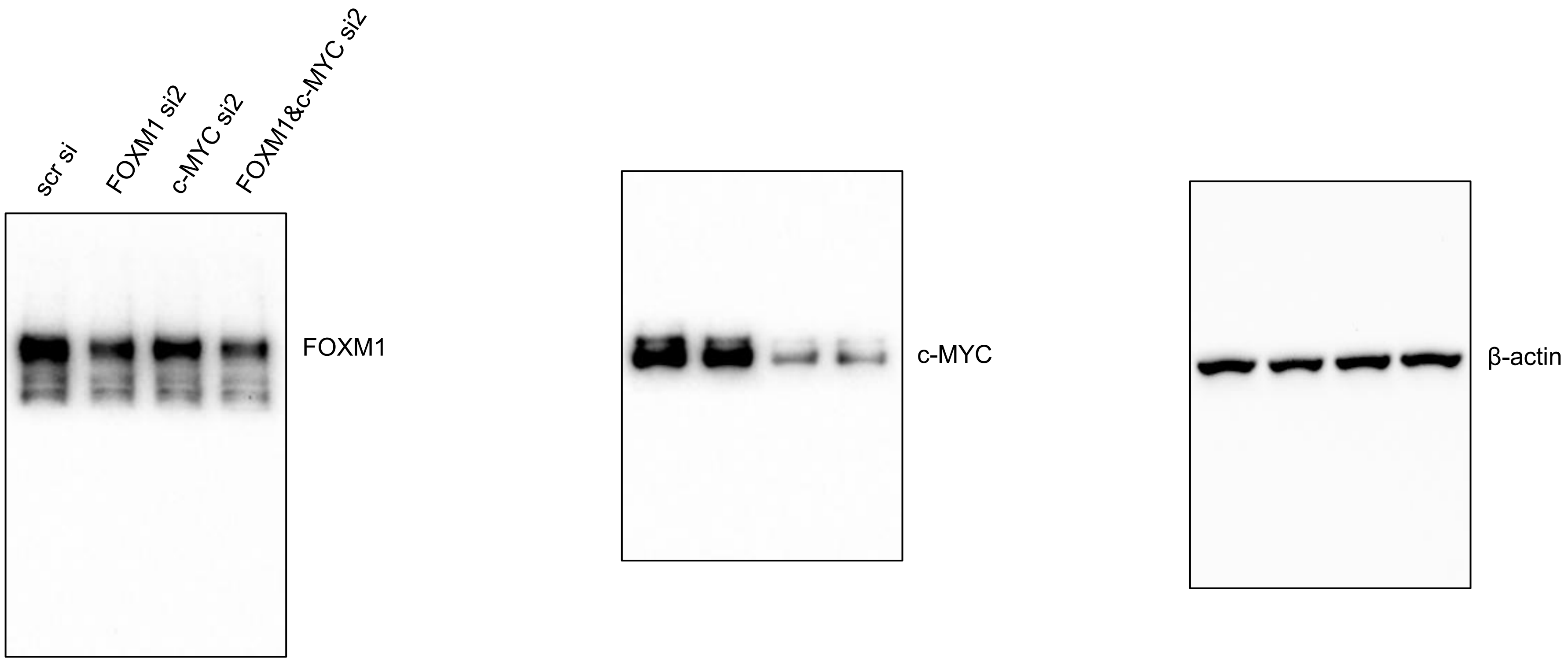

Figure 2

D

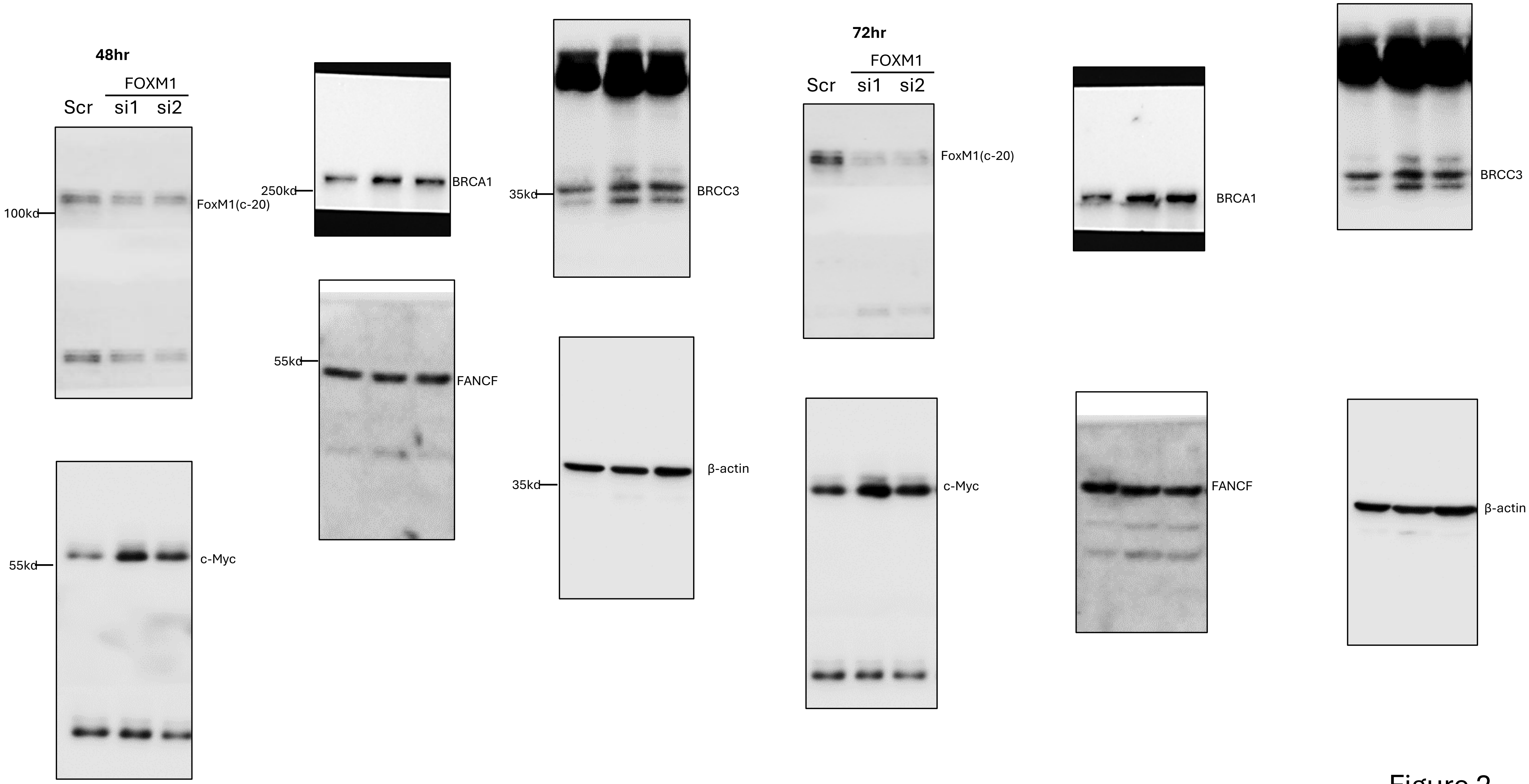
48hr
FOXM1
Scr
si1
si2
100kd
FoxM1(c-20)
55kd
c-Myc
250kd
BRCA1
55kd
FANCF
35kd
BRCC3
35kd
β-actin
72hr
FOXM1
Scr
si1
si2
FoxM1(c-20)
c-Myc
BRCA1
FANCF
BRCC3
β-actin

Figure 2

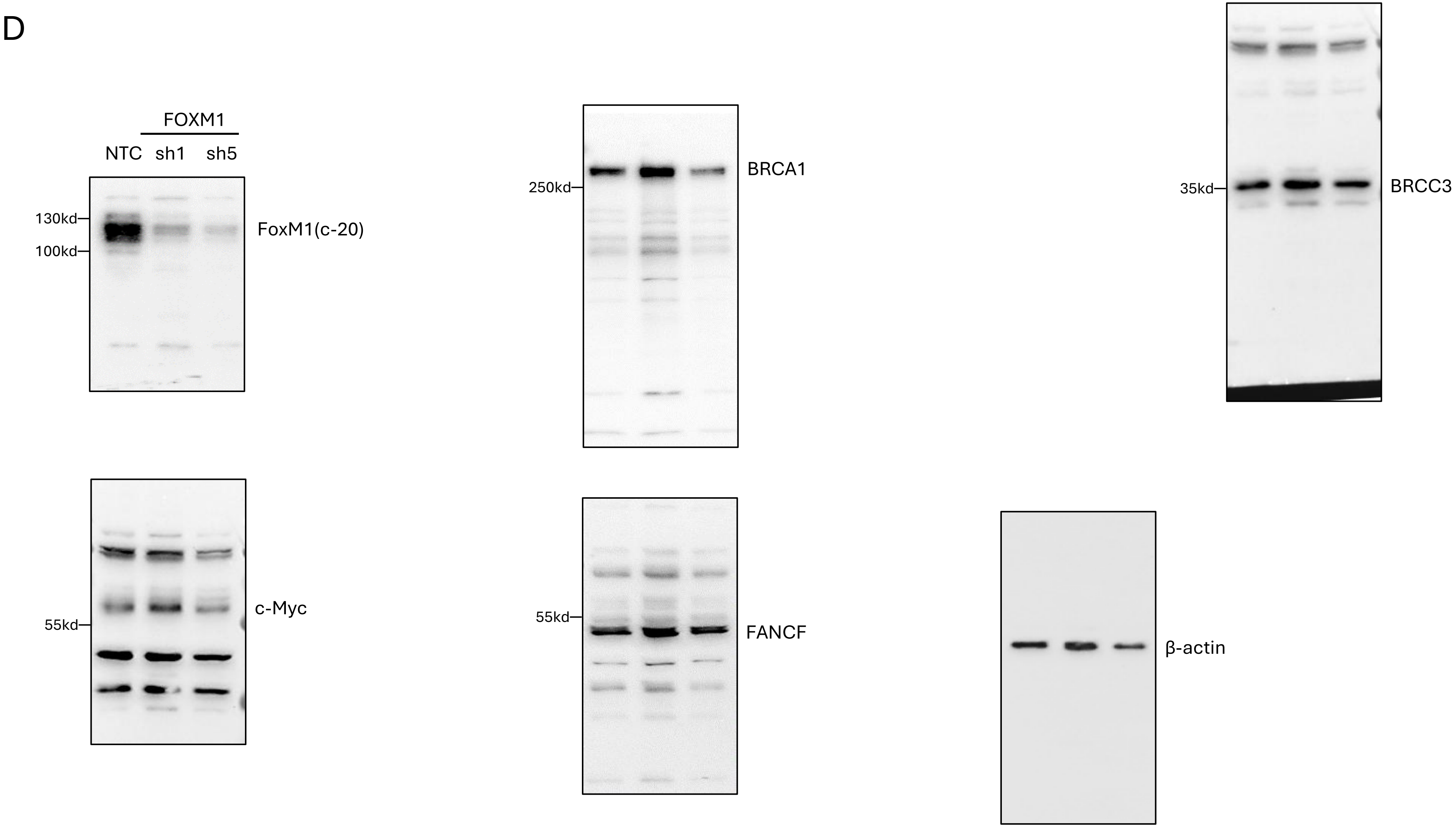
D
FOXM1
NTC sh1 sh5
130kd
100kd
FoxM1(c-20)
250kd
BRCA1
35kd
BRCC3
55kd
c-Myc
55kd
FANCF
β-actin

Figure 2

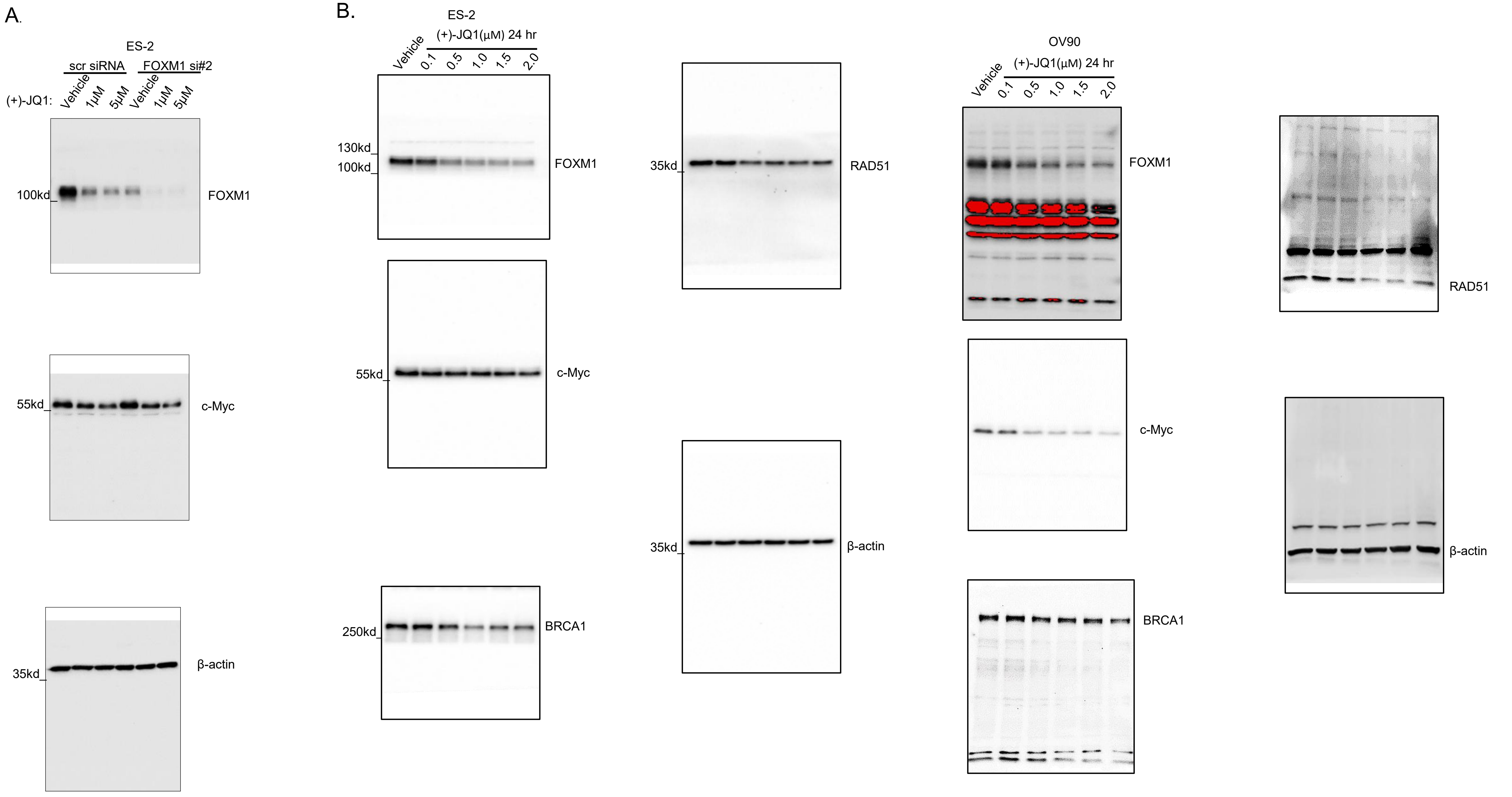
A.
ES-2
scr siRNA
FOXM1 si#2
(+)-JQ1:
Vehicle
1μM
5μM
Vehicle
1μM
5μM
100kd
FOXM1
55kd
c-Myc
35kd
β-actin
B.
ES-2
(+)-JQ1(μM) 24 hr
Vehicle
0.1
0.5
1.0
1.5
2.0
130kd
100kd
FOXM1
55kd
c-Myc
250kd
BRCA1
35kd
RAD51
35kd
β-actin
OV90
(+)-JQ1(μM) 24 hr
Vehicle
0.1
0.5
1.0
1.5
2.0
FOXM1
c-Myc
BRCA1
RAD51
β-actin

Figure 5

B.

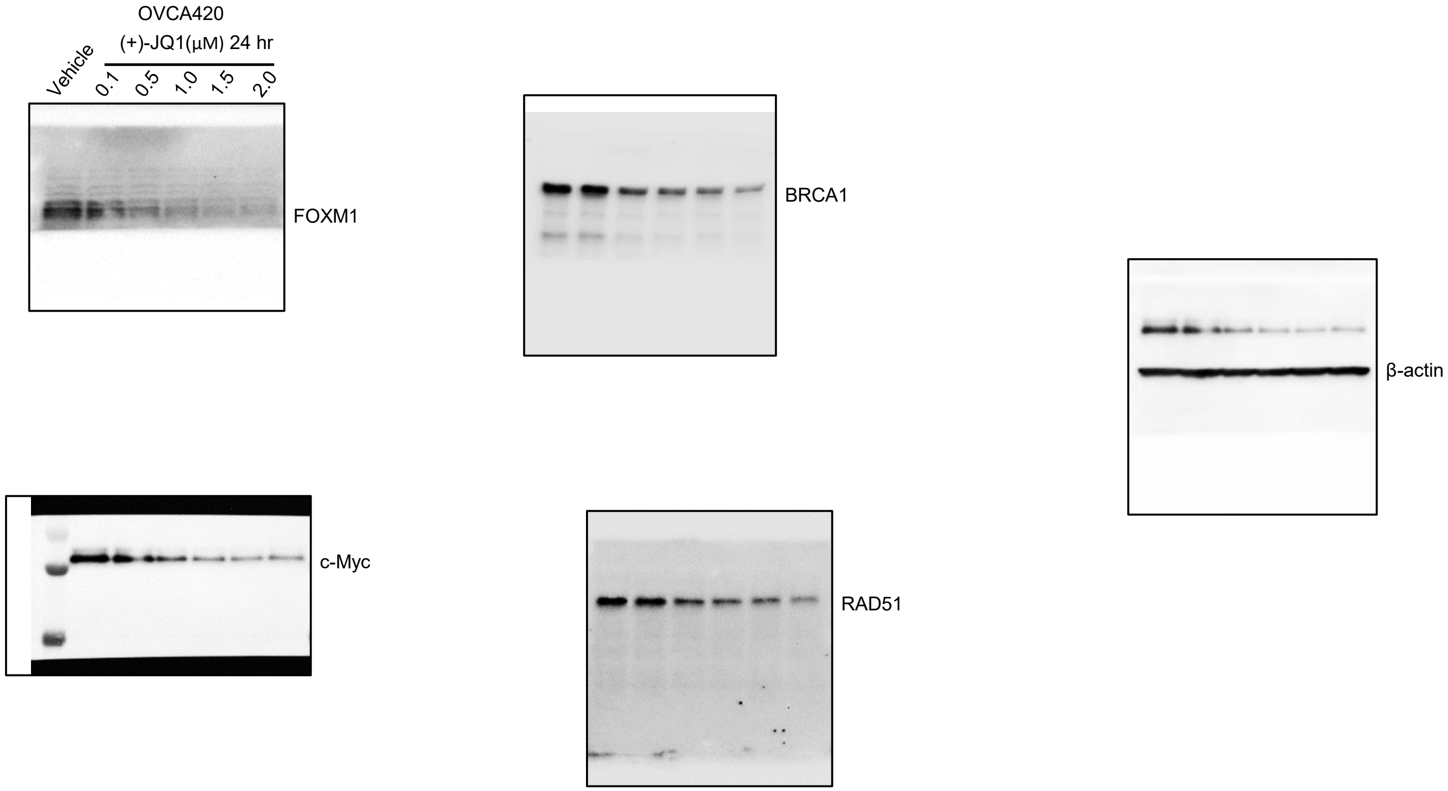

OVCA420
(+)-JQ1(µM) 24 hr
Vehicle
0.1
0.5
1.0
1.5
2.0
FOXM1
BRCA1
β-actin
c-Myc
RAD51

Figure 5

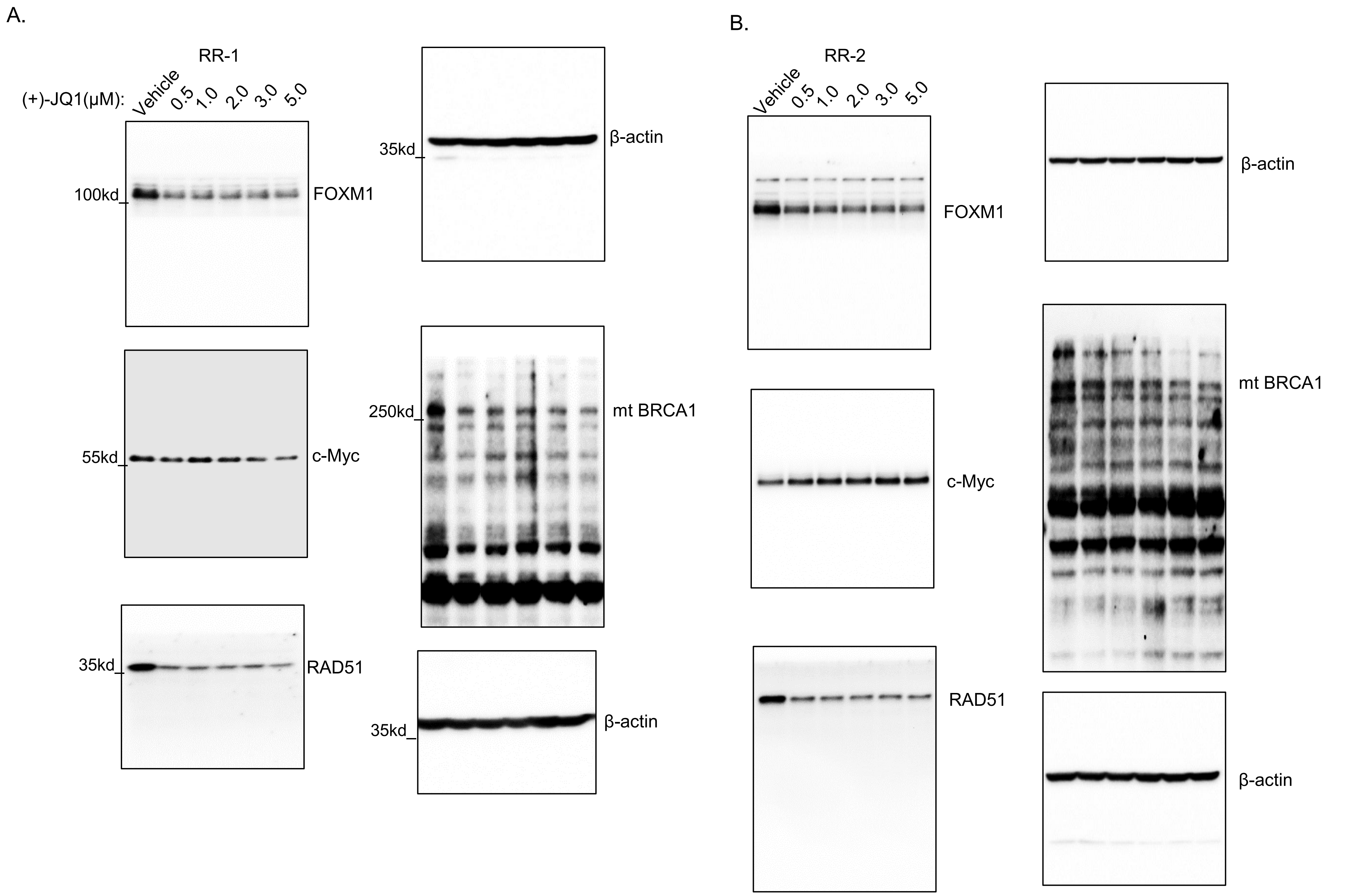
A.
RR-1
(+)-JQ1(μM):
Vehicle
0.5
1.0
2.0
3.0
5.0
100kd
FOXM1
55kd
c-Myc
35kd
RAD51
35kd
β-actin
250kd
mt BRCA1
35kd
β-actin
B.
RR-2
Vehicle
0.5
1.0
2.0
3.0
5.0
FOXM1
c-Myc
RAD51
β-actin
mt BRCA1
β-actin

Figure S6

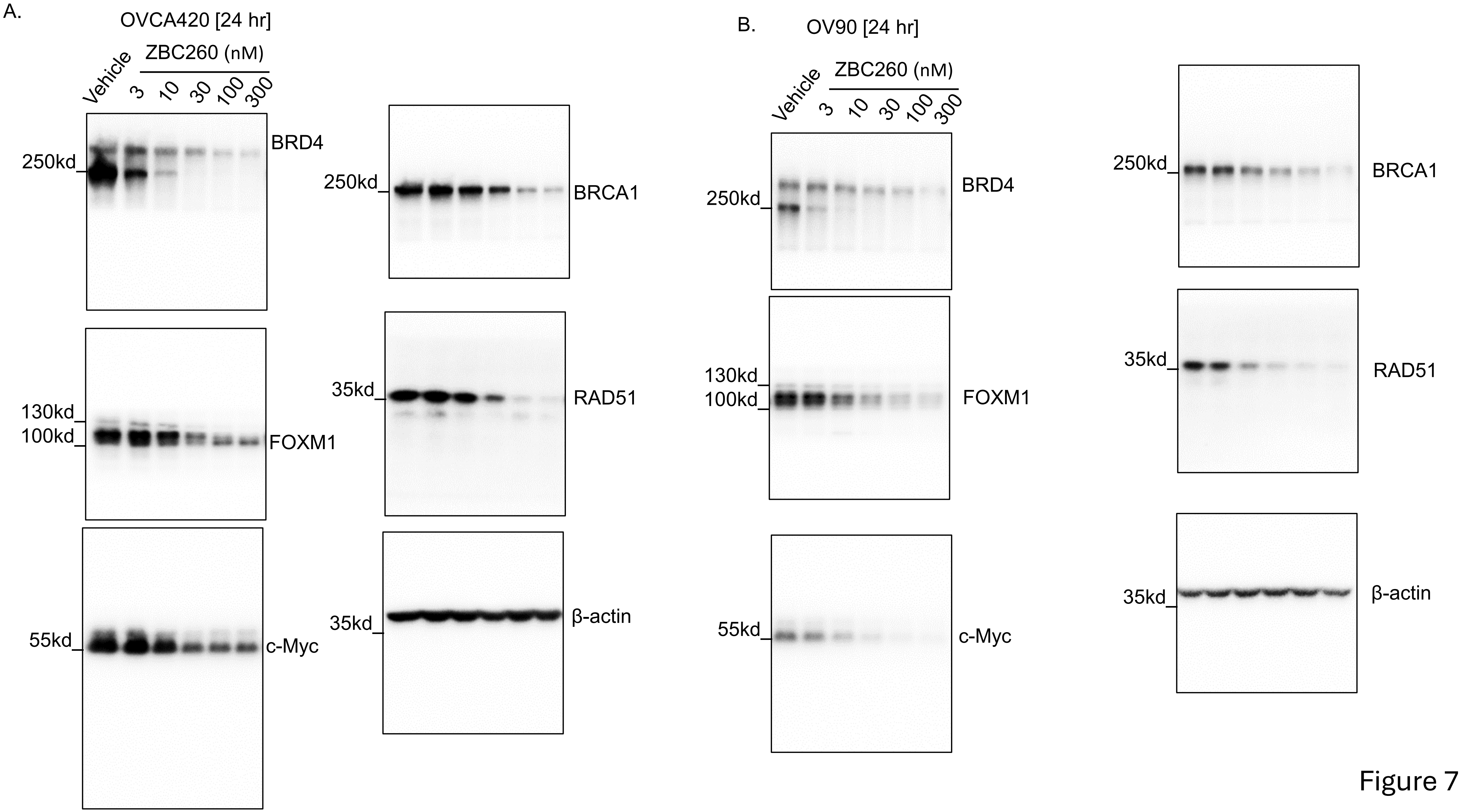
A.
OVCA420 [24 hr]
ZBC260 (nM)
Vehicle
3
10
30
100
300
250kd
BRD4
130kd
100kd
FOXM1
55kd
c-Myc
250kd
BRCA1
35kd
RAD51
35kd
β-actin
B.
OV90 [24 hr]
ZBC260 (nM)
Vehicle
3
10
30
100
300
250kd
BRD4
130kd
100kd
FOXM1
55kd
c-Myc
250kd
BRCA1
35kd
RAD51
35kd
β-actin

Figure 7

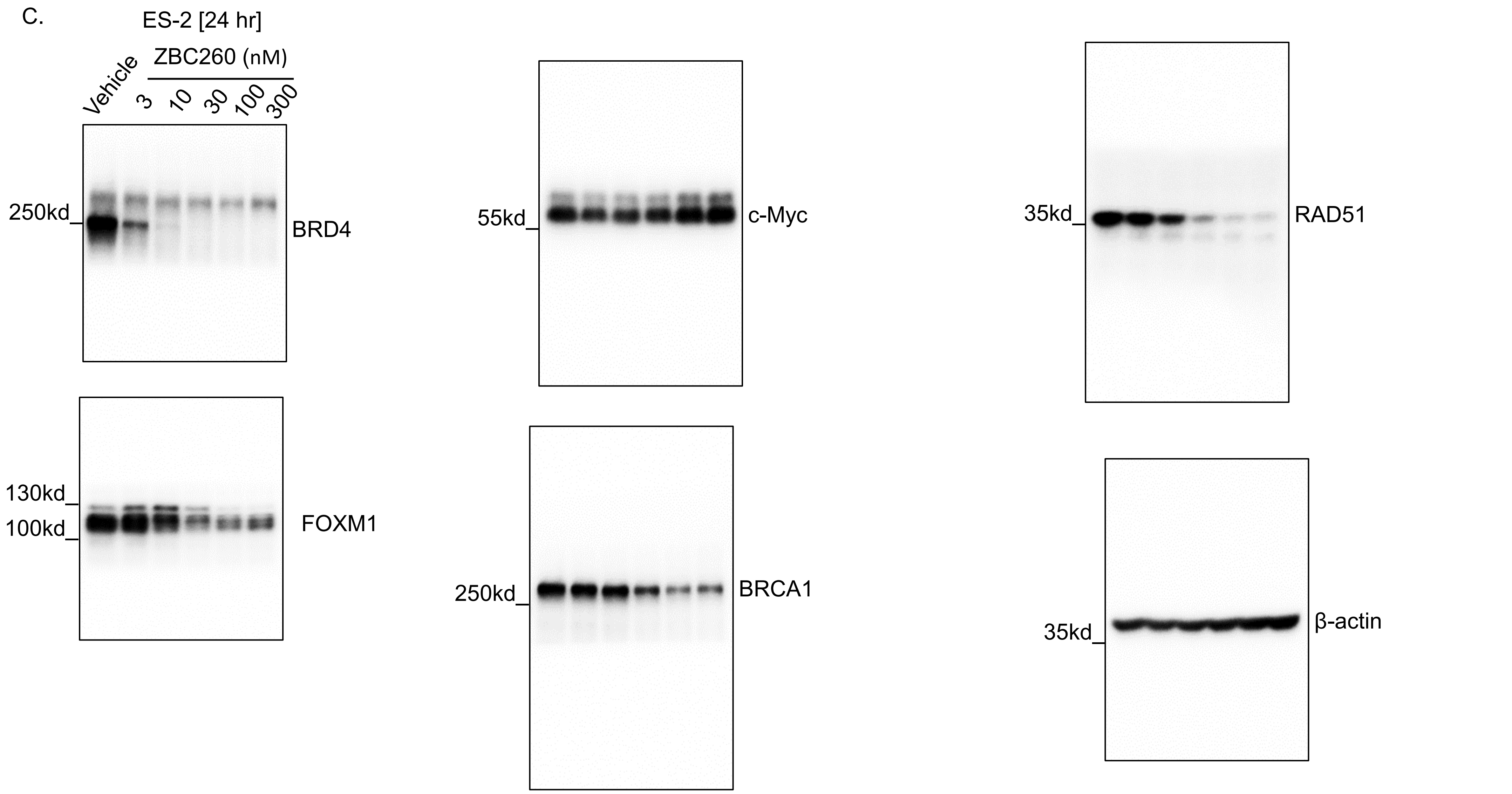
C.
ES-2 [24 hr]
ZBC260 (nM)
Vehicle
3
10
30
100
300
250kd
BRD4
130kd
100kd
FOXM1
55kd
c-Myc
250kd
BRCA1
35kd
RAD51
35kd
β-actin

Figure 7

Scatter plot from High-content imaging

Code

Hide

```
# Load necessary libraries
library(dplyr)
```

```
Attaching package: ‘dplyr’

The following objects are masked from ‘package:stats’:

    filter, lag

The following objects are masked from ‘package:base’:

    intersect, setdiff, setequal, union
```

Hide

```
library(ggplot2)
```

```
Need help getting started? Try the R Graphics Cookbook: https://r-graphics.org
```

Hide

```
df <- read.csv("~/Downloads/Olap_Zbc_pH2AX_2_24_25_T1_ExperimentCellData.csv")
```

Hide

```
dim(df)
```

```
[1] 30825    18
```

Hide

```
head(df)
```

	T.Id <int>	Condition <chr>	Well.Name <chr>	Positive <int>	Nuclear.Area <dbl>	Cell.Area <dbl>
1	1	NT	C1	0	165.2067	165.2067
2	1	NT	C1	0	123.7860	123.7860
3	1	NT	C1	0	144.2583	144.2583
4	1	NT	C1	0	198.0576	198.0576

	T.Id <int>	Condition <chr>	Well.Name <chr>	Positive <int>	Nuclear.Area <dbl>	Cell.Area <dbl>
5	1	NT	C1	0	252.8091	252.8091
6	1	NT	C1	1	301.3713	301.3713

6 rows | 1-7 of 18 columns

Hide

```
unique(df$Condition)
```

```
[1] "NT"    "Olap"  "ZBC"   "Combo"
```

This step remove outliers (staining artifacts)

Hide

```
Q1 <- quantile(df$Wavelength.1.Integrated.Nuclear.Intensity, 0.25)
Q3 <- quantile(df$Wavelength.1.Integrated.Nuclear.Intensity, 0.75)
IQR <- Q3 - Q1
lower_bound <- Q1 - 1.5 * IQR
upper_bound <- Q3 + 1.5 * IQR
df_clean <- df[df$Wavelength.1.Integrated.Nuclear.Intensity >= lower_bound & df$Wavelength.1.Int
egrated.Nuclear.Intensity <= upper_bound, ]
```

Hide

```
plot(density(df_clean$Wavelength.1.Integrated.Nuclear.Intensity), main="Density Plot of DAPI In
t. Intensity of All Filtered Cells", xlab="DAPI AFU")
```

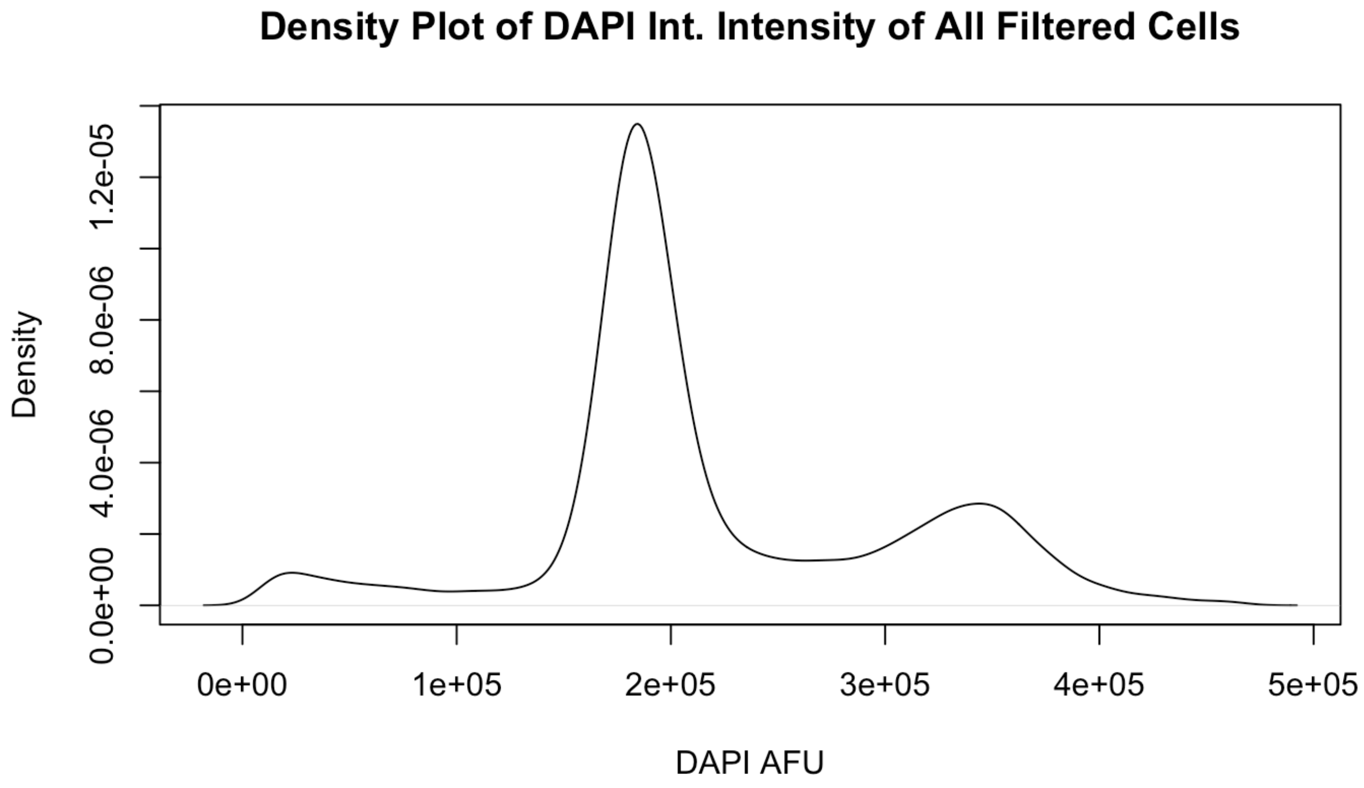

Hide

```
df_nt <- df_clean[df_clean$Condition == "NT", ]
plot(density(df_nt$Wavelength.1.Integrated.Nuclear.Intensity), main="Density Plot of DAPI Int. I
ntensity in Untreated Control", xlab="DAPI AFU")
```

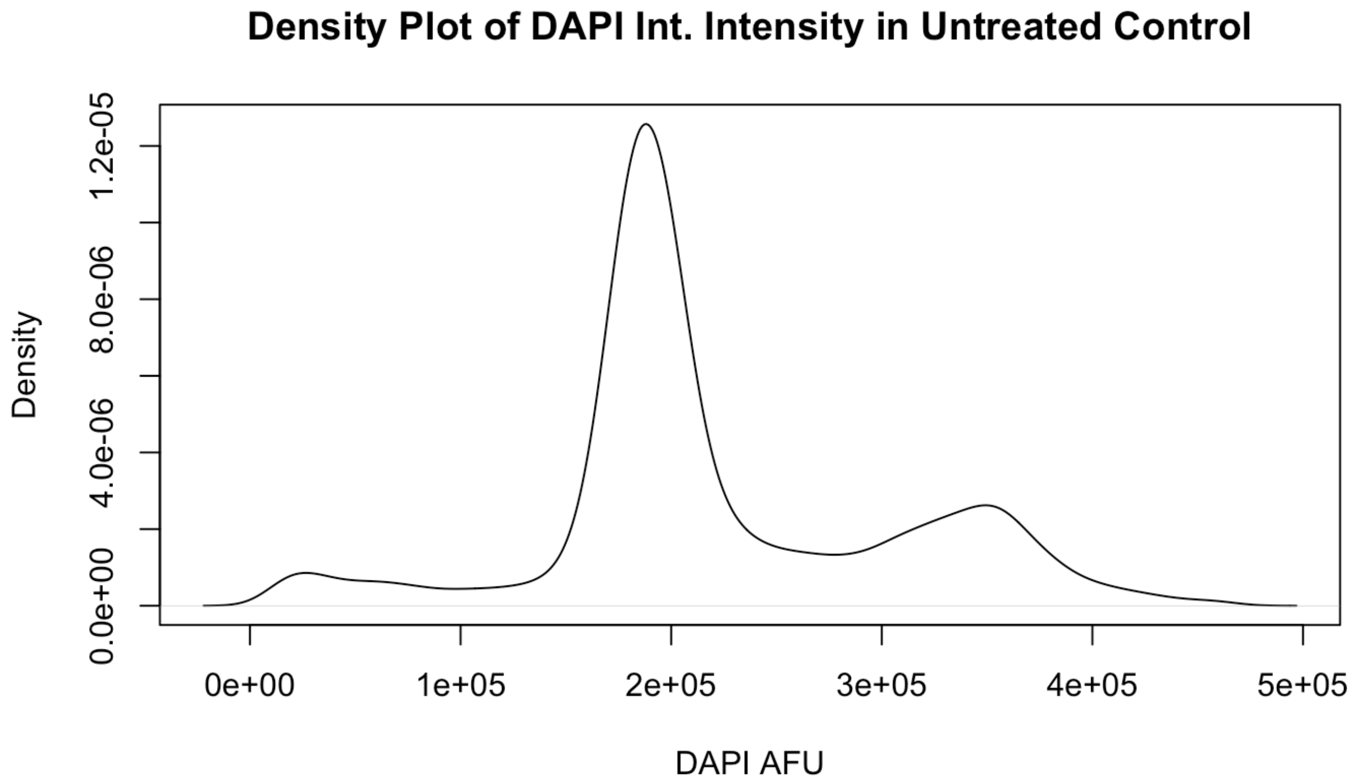

Hide

```
df_olap <- df_clean[df_clean$Condition == "Olap", ]
plot(density(df_olap$Wavelength.1.Integrated.Nuclear.Intensity), main="Density Plot of DAPI Int.
Intensity in Olaparib Treated Cells", xlab="DAPI AFU")
```

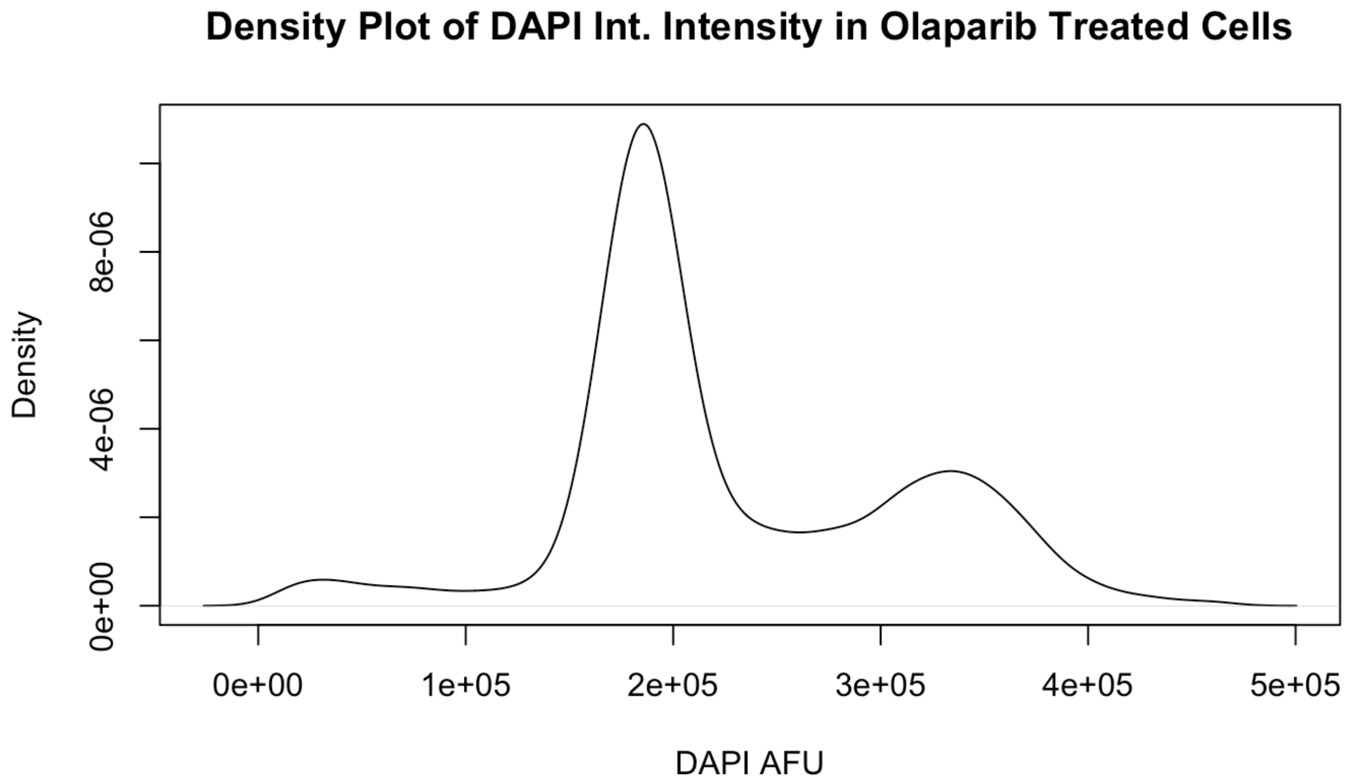

Hide

```
df_zbc <- df_clean[df_clean$Condition == "ZBC", ]
plot(density(df_zbc$Wavelength.1.Integrated.Nuclear.Intensity), main="Density Plot of DAPI Int.
Intensity in ZBC Treated Cells", xlab="DAPI AFU")
```

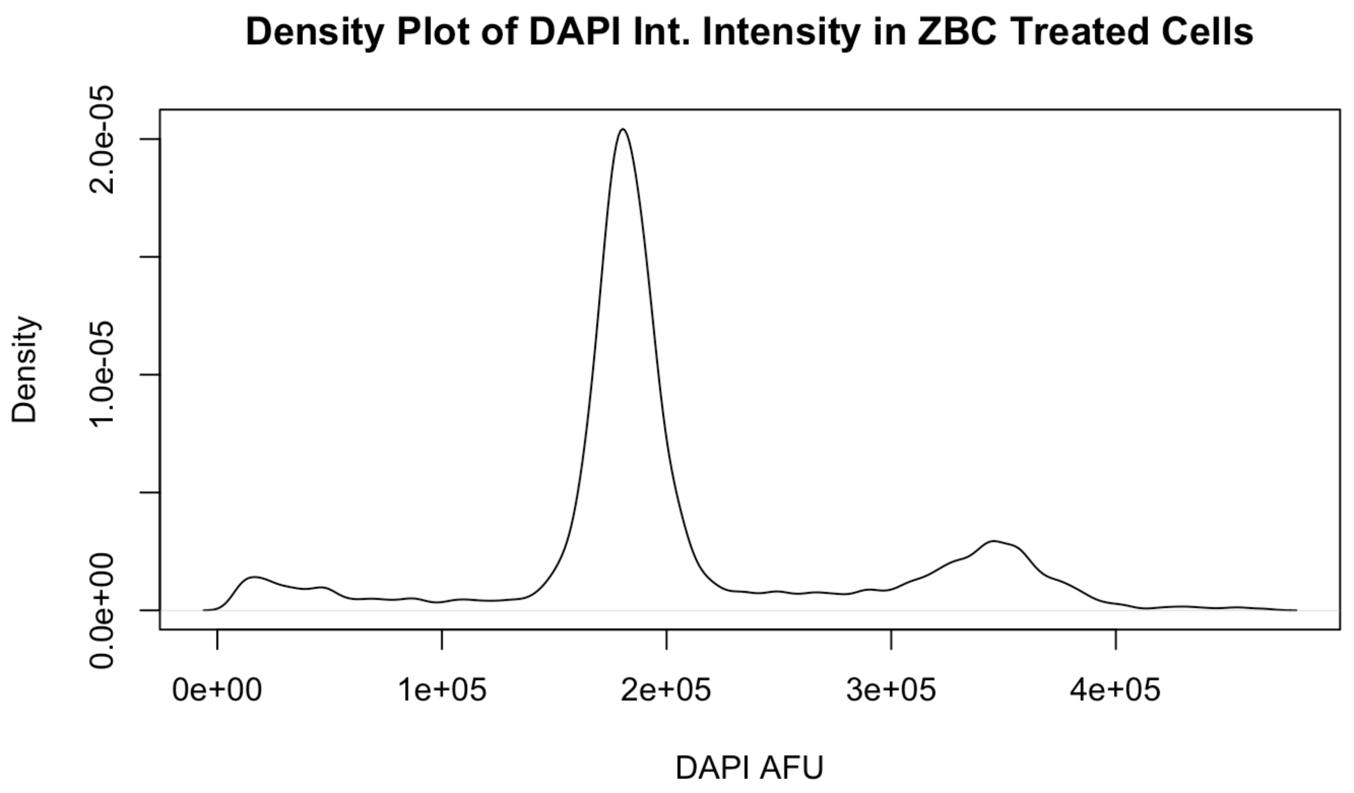

Hide

```
df_combo <- df_clean[df_clean$Condition == "Combo", ]
plot(density(df_combo$Wavelength.1.Integrated.Nuclear.Intensity), main="Density Plot of DAPI In
t. Intensity in Combo", xlab="DAPI AFU")
```

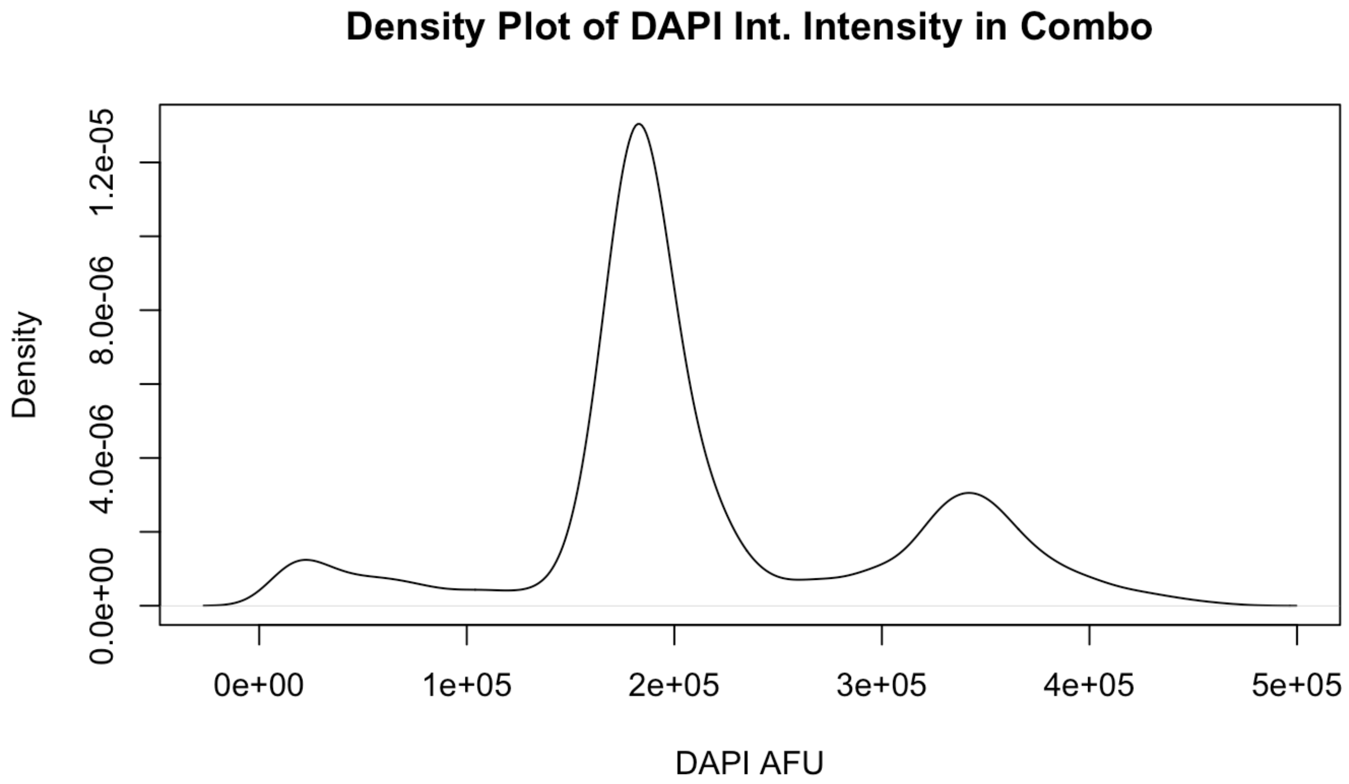
Density Plot of DAPI Int. Intensity in Combo
Density
0.0e+00
4.0e-06
8.0e-06
1.2e-05
0e+00
1e+05
2e+05
3e+05
4e+05
5e+05
DAPI AFU

Hide

```
# Define the function
generate_scatter_plot <- function(data, condition_name) {
  # Subset the dataframe for the specific condition
  subset_data <- data %>%
    filter(Condition == condition_name)

  # Check if the subset is not empty
  if (nrow(subset_data) == 0) {
    stop("No data found for the specified condition.")
  }

# Perform a scatter plot
plot <- ggplot(subset_data, aes(x = Wavelength.1.Integrated.Nuclear.Intensity, y = Wavelength.2.
Integrated.Nuclear.Intensity)) +
    geom_point(aes(color = Wavelength.2.Integrated.Nuclear.Intensity >= 0.5e5)) +
    scale_color_manual(values = c("FALSE" = "black", "TRUE" = "red"),
                       labels = c("FALSE" = "pH2AX(-)", "TRUE" = "pH2AX(+)")) +
    scale_y_log10() +
    labs(title = paste("Scatterplot of DAPI vs pH2AX for", condition_name),
         x = "DAPI",
         y = "pH2AX",
         color = "pH2AX Status") +
    coord_cartesian(ylim = c(NA, 5e5))

# Print the plot
print(plot)
}
```

Hide

```
generate_scatter_plot(df_clean, "NT")
```

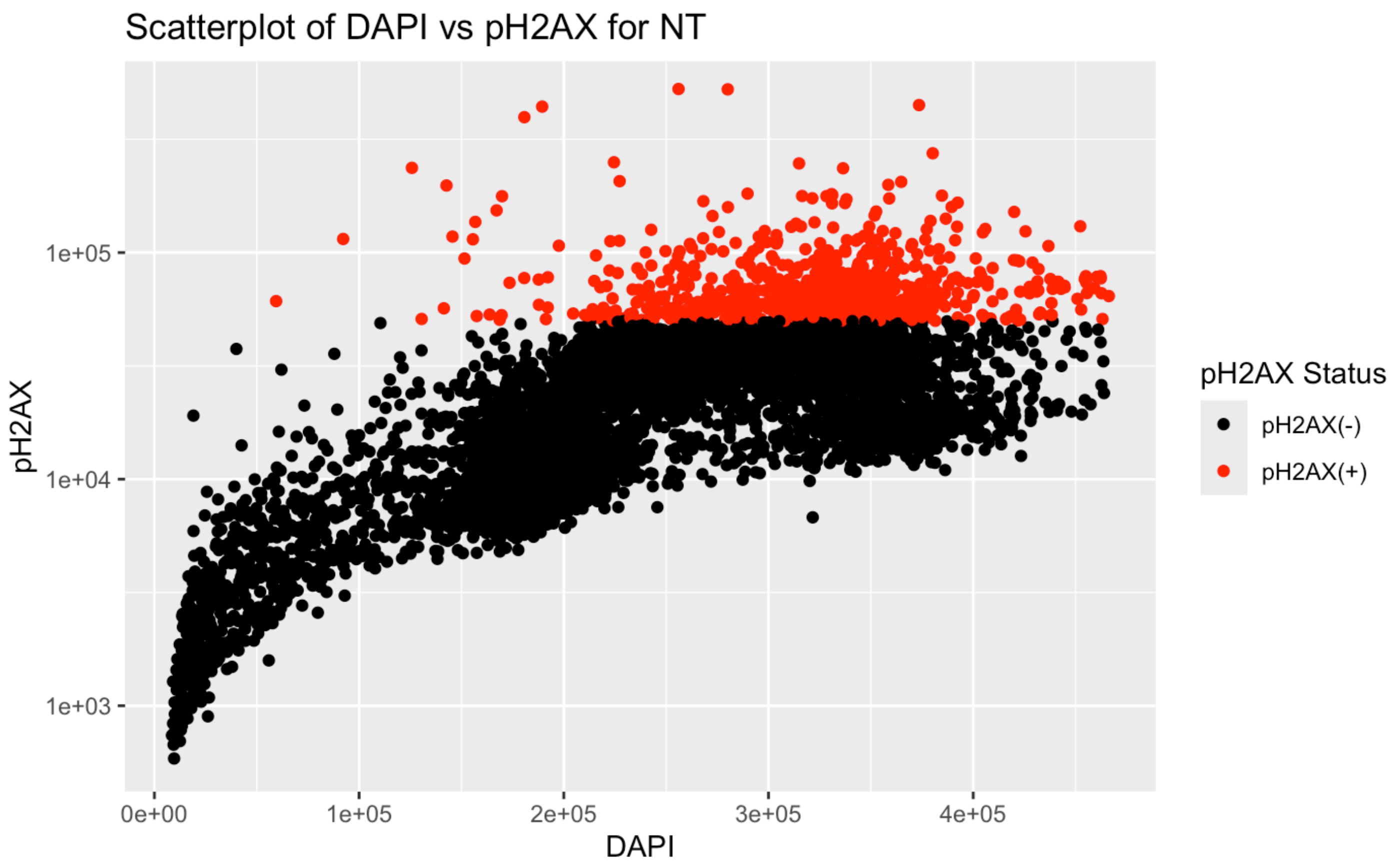

Hide

```
subset_data <- df_clean %>%
    filter(Condition == "NT")
count <- subset_data %>%
  filter(
    `Wavelength.2.Integrated.Nuclear.Intensity` >= 0.5e5,
    (`Wavelength.1.Integrated.Nuclear.Intensity` >= 2.3e5 & `Wavelength.1.Integrated.Nuclear.Int
ensity` <= 3.1e5)
  ) %>%
  nrow()

print(count)
```

```
[1] 231
```

To check how many cells are pH2AX positive in each cell cycle phases (G1, S, G2M):

Hide

```
subset_NT <- df_clean %>%
    filter(Condition == "NT")
results <- subset_NT %>%
  filter(`Wavelength.2.Integrated.Nuclear.Intensity` >= 0.5e5) %>%
  summarise(
    G1 = sum(`Wavelength.1.Integrated.Nuclear.Intensity` < 2.3e5),
    S = sum(`Wavelength.1.Integrated.Nuclear.Intensity` >= 2.3e5 &
                 `Wavelength.1.Integrated.Nuclear.Intensity` <= 3.1e5),
    G2M = sum(`Wavelength.1.Integrated.Nuclear.Intensity` > 3.1e5)
  )

# Print results
cat("Table: pH2AX-positive cells by cell cycle phase in Untreated Group\n\n")
```

```
Table: pH2AX-positive cells by cell cycle phase in Untreated Group
```

Hide

```
print(results)
```

G1 <int>	S <int>	G2M <int>
52	231	485

1 row

To check how many cells are in each cell cycle phases (G1, S, G2M):

Hide

```
subset_NT <- df_clean %>%
    filter(Condition == "NT")
results <- subset_NT %>%
  summarise(
    G1 = sum(`Wavelength.1.Integrated.Nuclear.Intensity` < 2.3e5),
    S = sum(`Wavelength.1.Integrated.Nuclear.Intensity` >= 2.3e5 &
                 `Wavelength.1.Integrated.Nuclear.Intensity` <= 3.1e5),
    G2M = sum(`Wavelength.1.Integrated.Nuclear.Intensity` > 3.1e5)
  )

# Print results
cat("Table: Total cells by cell cycle phase in Untreated Group\n\n")
```

```
Table: Total cells by cell cycle phase in Untreated Group
```

Hide

```
print(results)
```

G1	S	G2M
<int>	<int>	<int>
6219	1091	1792

1 row

Hide

```
generate_scatter_plot(df_clean, "Olap")
```

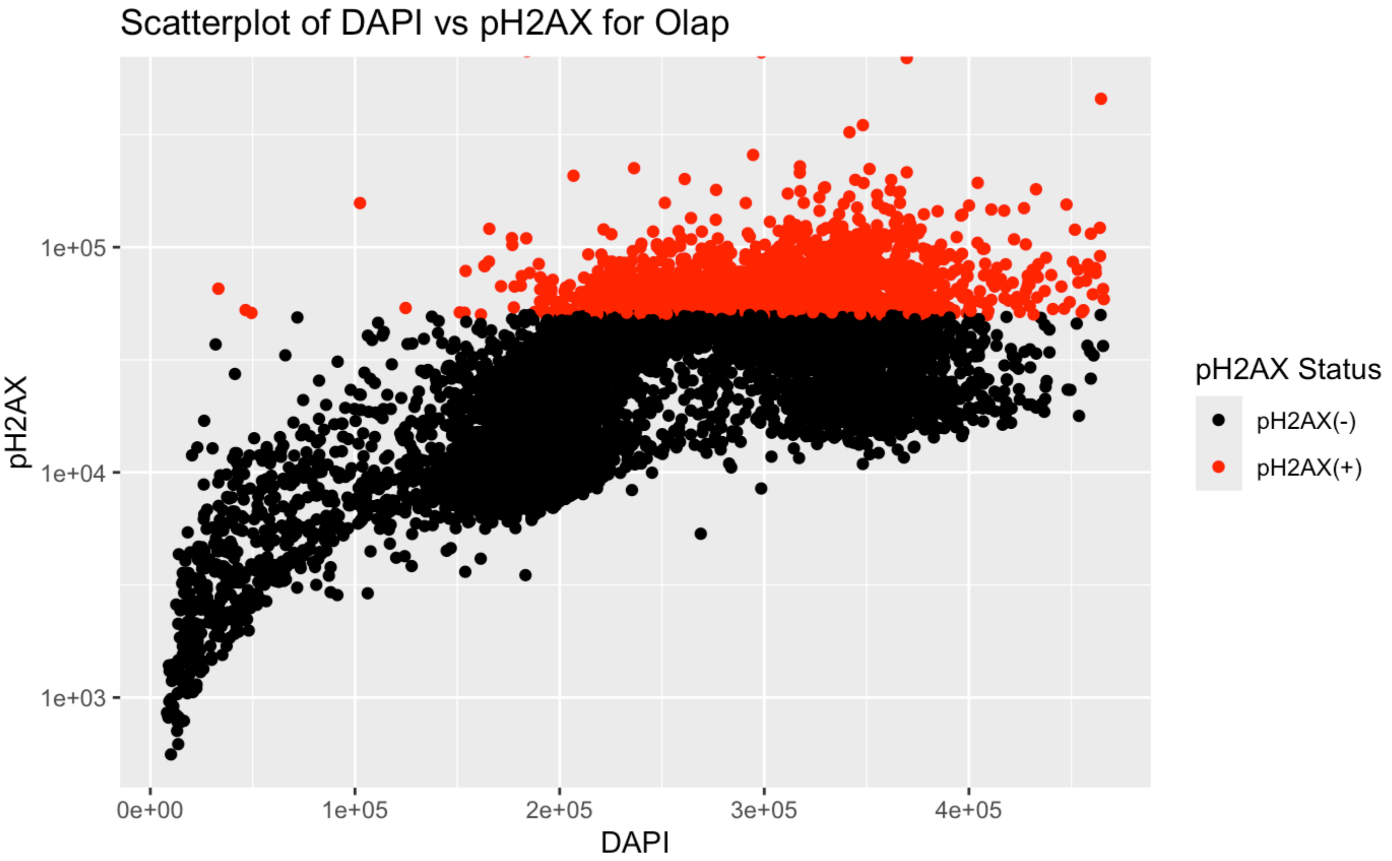

Hide

```
subset_data <- df_clean %>%
    filter(Condition == "Olap")
count <- subset_data %>%
  filter(
    `Wavelength.2.Integrated.Nuclear.Intensity` >= 0.5e5,
    (`Wavelength.1.Integrated.Nuclear.Intensity` >= 2.3e5 & `Wavelength.1.Integrated.Nuclear.Int
ensity` <= 3.1e5)
  ) %>%
  nrow()

print(count)
```

```
[1] 743
```

Hide

```
subset_Olap <- df_clean %>%
    filter(Condition == "Olap")
results <- subset_Olap %>%
  filter(`Wavelength.2.Integrated.Nuclear.Intensity` >= 0.5e5) %>%
  summarise(
    G1 = sum(`Wavelength.1.Integrated.Nuclear.Intensity` < 2.3e5),
    S = sum(`Wavelength.1.Integrated.Nuclear.Intensity` >= 2.3e5 &
                 `Wavelength.1.Integrated.Nuclear.Intensity` <= 3.1e5),
    G2M = sum(`Wavelength.1.Integrated.Nuclear.Intensity` > 3.1e5)
  )

# Print results
print(results)
```

G1 <int>	S <int>	G2M <int>
135	743	1002

1 row

To check how many cells are in each cell cycle phases (G1, S, G2M):

Hide

```
subset_Olap <- df_clean %>%
    filter(Condition == "Olap")
results <- subset_Olap %>%
  summarise(
    G1 = sum(`Wavelength.1.Integrated.Nuclear.Intensity` < 2.3e5),
    S = sum(`Wavelength.1.Integrated.Nuclear.Intensity` >= 2.3e5 &
                 `Wavelength.1.Integrated.Nuclear.Intensity` <= 3.1e5),
    G2M = sum(`Wavelength.1.Integrated.Nuclear.Intensity` > 3.1e5)
  )

# Print results
cat("Table: Total cells by cell cycle phase in Olaparib-treated Group\n\n")
```

```
Table: Total cells by cell cycle phase in Olaparib-treated Group
```

Hide

```
print(results)
```

G1 <int>	S <int>	G2M <int>
6863	1589	2390

1 row

Hide

```
generate_scatter_plot(df_clean, "ZBC")
```

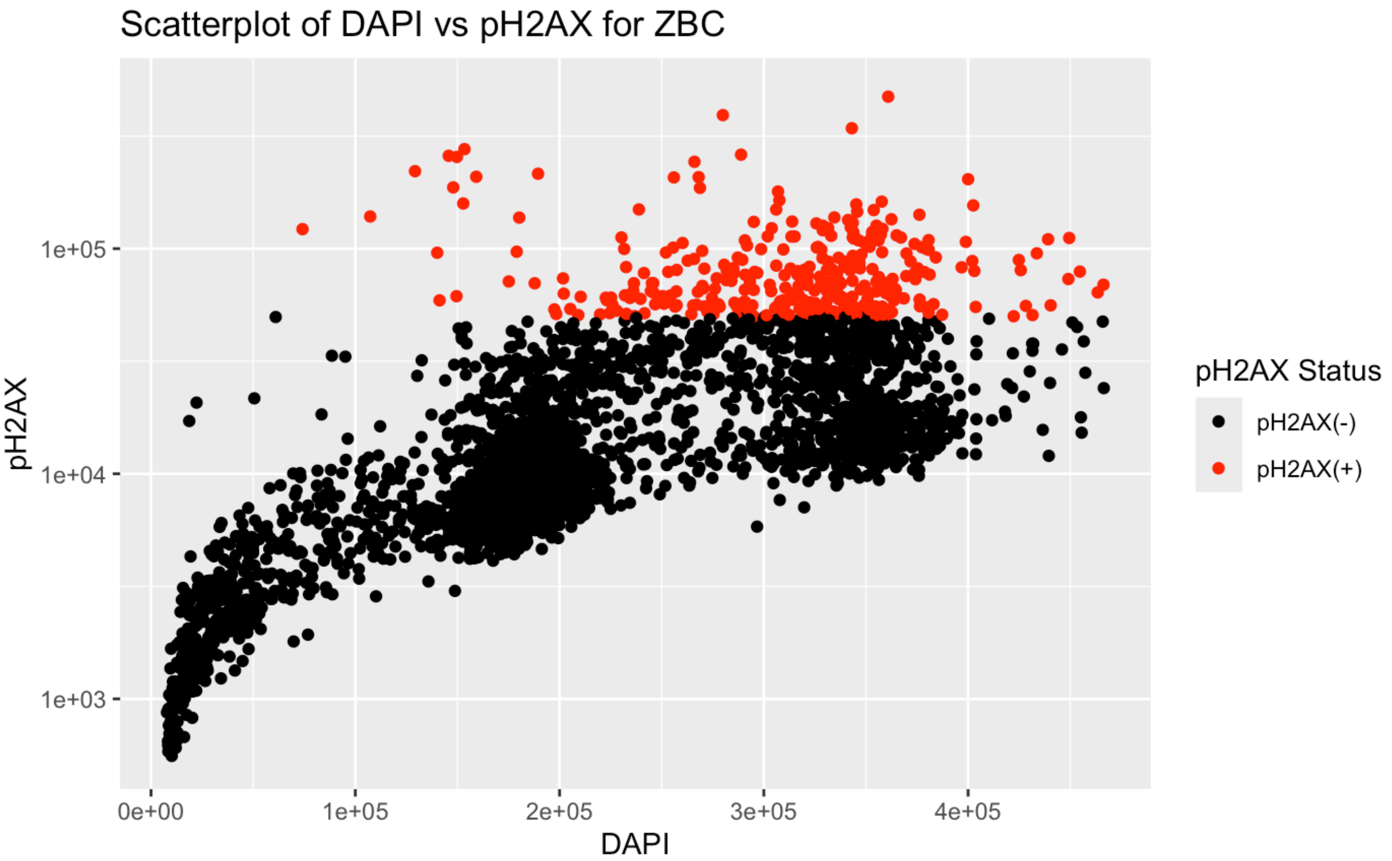

Hide

```
subset_data <- df_clean %>%
    filter(Condition == "ZBC")
count <- subset_data %>%
  filter(
    `Wavelength.2.Integrated.Nuclear.Intensity` >= 0.5e5,
    (`Wavelength.1.Integrated.Nuclear.Intensity` >= 2.3e5 & `Wavelength.1.Integrated.Nuclear.Int
ensity` <= 3.1e5)
  ) %>%
  nrow()

print(count)
```

```
[1] 104
```

Hide

```
subset_ZBC <- df_clean %>%
    filter(Condition == "ZBC")
results <- subset_ZBC %>%
  filter(`Wavelength.2.Integrated.Nuclear.Intensity` >= 0.5e5) %>%
  summarise(
    G1 = sum(`Wavelength.1.Integrated.Nuclear.Intensity` < 2.3e5),
    S = sum(`Wavelength.1.Integrated.Nuclear.Intensity` >= 2.3e5 &
                 `Wavelength.1.Integrated.Nuclear.Intensity` <= 3.1e5),
    G2M = sum(`Wavelength.1.Integrated.Nuclear.Intensity` > 3.1e5)
  )

# Print results
print(results)
```

G1 <int>	S <int>	G2M <int>
31	104	192

1 row

To check how many cells are in each cell cycle phases (G1, S, G2M):

Hide

```
subset_ZBC <- df_clean %>%
    filter(Condition == "ZBC")
results <- subset_ZBC %>%
  summarise(
    G1 = sum(`Wavelength.1.Integrated.Nuclear.Intensity` < 2.3e5),
    S = sum(`Wavelength.1.Integrated.Nuclear.Intensity` >= 2.3e5 &
                 `Wavelength.1.Integrated.Nuclear.Intensity` <= 3.1e5),
    G2M = sum(`Wavelength.1.Integrated.Nuclear.Intensity` > 3.1e5)
  )

# Print results
cat("Table: Total cells by cell cycle phase in ZBC-treated Group\n\n")
```

```
Table: Total cells by cell cycle phase in ZBC-treated Group
```

Hide

```
print(results)
```

G1 <int>	S <int>	G2M <int>
4158	354	882

1 row

Hide

```
generate_scatter_plot(df_clean, "Combo")
```

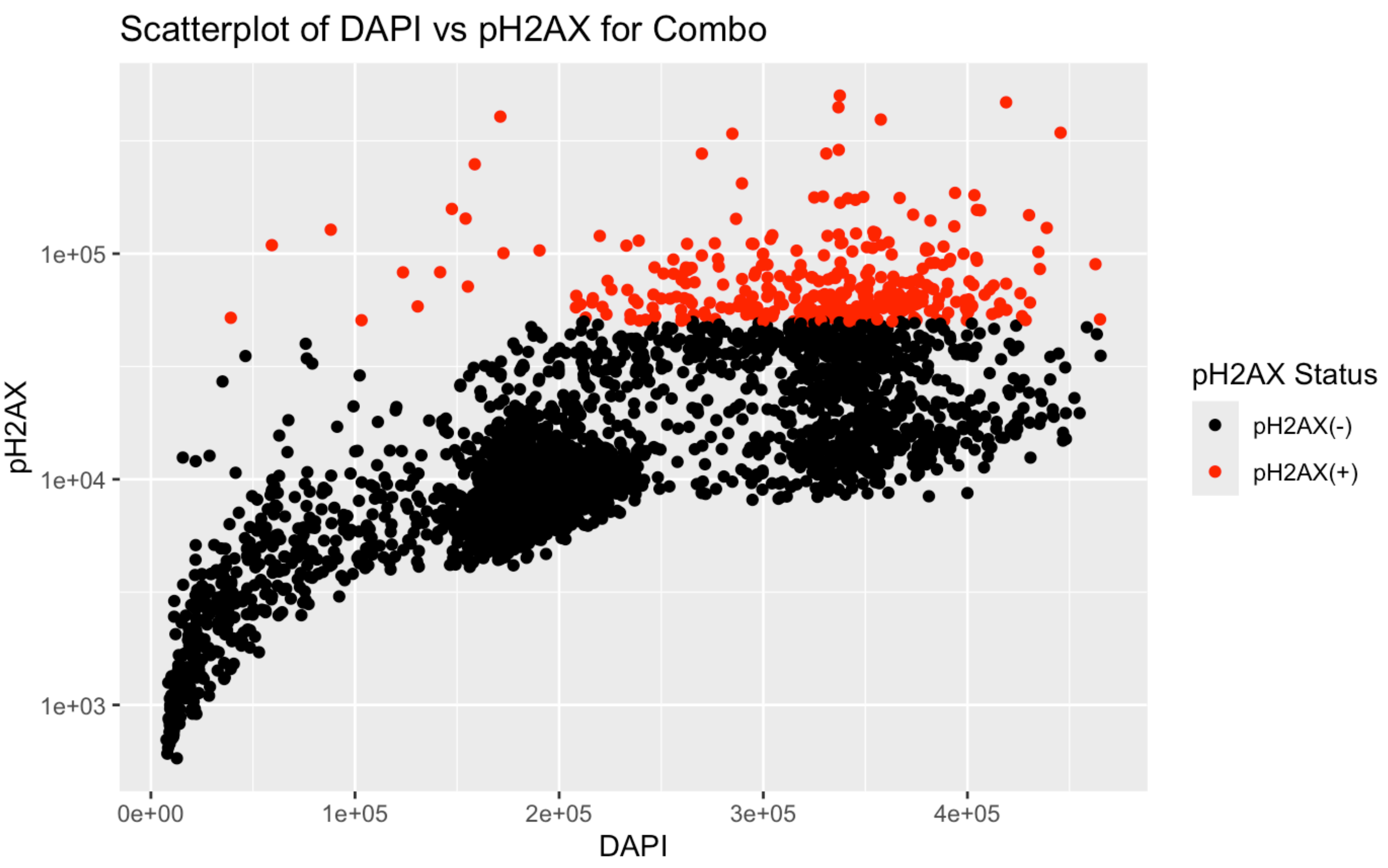

Hide

```
subset_data <- df_clean %>%
    filter(Condition == "Combo")
count <- subset_data %>%
  filter(
    `Wavelength.2.Integrated.Nuclear.Intensity` >= 0.5e5,
    (`Wavelength.1.Integrated.Nuclear.Intensity` >= 2.3e5 & `Wavelength.1.Integrated.Nuclear.Int
ensity` <= 3.1e5)
  ) %>%
  nrow()

print(count)
```

```
[1] 85
```

Hide

```
subset_Combo <- df_clean %>%
    filter(Condition == "Combo")
results <- subset_Combo %>%
  filter(`Wavelength.2.Integrated.Nuclear.Intensity` >= 0.5e5) %>%
  summarise(
    G1 = sum(`Wavelength.1.Integrated.Nuclear.Intensity` < 2.3e5),
    S = sum(`Wavelength.1.Integrated.Nuclear.Intensity` >= 2.3e5 &
             `Wavelength.1.Integrated.Nuclear.Intensity` <= 3.1e5),
    G2M = sum(`Wavelength.1.Integrated.Nuclear.Intensity` > 3.1e5)
  )

# Print results
print(results)
```

G1 <int>	S <int>	G2M <int>
25	85	237

1 row

To check how many cells are in each cell cycle phases (G1, S, G2M):

Hide

```
subset_Combo <- df_clean %>%
    filter(Condition == "Combo")
results <- subset_Combo %>%
  summarise(
    G1 = sum(`Wavelength.1.Integrated.Nuclear.Intensity` < 2.3e5),
    S = sum(`Wavelength.1.Integrated.Nuclear.Intensity` >= 2.3e5 &
             `Wavelength.1.Integrated.Nuclear.Intensity` <= 3.1e5),
    G2M = sum(`Wavelength.1.Integrated.Nuclear.Intensity` > 3.1e5)
  )

# Print results
cat("Table: Total cells by cell cycle phase in Combo-treated Group\n\n")
```

```
Table: Total cells by cell cycle phase in Combo-treated Group
```

Hide

```
print(results)
```

G1 <int>	S <int>	G2M <int>
3375	314	998

1 row
